# Toxigenic *Clostridium perfringens* Isolated from At-Risk Paediatric Inflammatory Bowel Disease Patients

**DOI:** 10.1093/ecco-jcc/jjae016

**Published:** 2024-01-24

**Authors:** James Kuo, Jasmina Uzunovic, Amanda Jacobson, Michelle Dourado, Sarah Gierke, Manohary Rajendram, Daniela Keilberg, Jordan Mar, Emily Stekol, Joanna Curry, Sofia Verstraete, Jessica Lund, Yuxin Liang, Fiona B Tamburini, Natalie S Omattage, Matthieu Masureel, Steven T Rutherford, David H Hackos, Man-Wah Tan, Allyson L Byrd, Mary E Keir, Elizabeth Skippington, Kelly M Storek

**Affiliations:** Department of Infectious Diseases and Host-Microbe Interactions, Genentech Inc., South San Francisco, CA, USA; Department of Bioinformatics, Genentech Inc., South San Francisco, CA, USA; Department of Immunology Discovery, Genentech Inc., South San Francisco, CA, USA; Department of Neuroscience, Genentech Inc., South San Francisco, CA, USA; Department of Pathology, Genentech Inc., South San Francisco, CA, USA; Department of Infectious Diseases and Host-Microbe Interactions, Genentech Inc., South San Francisco, CA, USA; Department of Infectious Diseases and Host-Microbe Interactions, Genentech Inc., South San Francisco, CA, USA; Department of Human Pathobiology and OMNI Reverse Translation, Genentech Inc., South San Francisco, CA, USA; Department of Pediatrics, University of California San Francisco Benioff Children’s Hospital, San Francisco, CA, 94158, USA; Department of Pediatrics, University of California San Francisco Benioff Children’s Hospital, San Francisco, CA, 94158, USA; Department of Pediatrics, University of California San Francisco Benioff Children’s Hospital, San Francisco, CA, 94158, USA; Department of Microchemistry, Proteomics & Lipidomics, Genentech Inc., South San Francisco, CA, USA; Department of Microchemistry, Proteomics & Lipidomics, Genentech Inc., South San Francisco, CA, USA; Department of Human Pathobiology and OMNI Reverse Translation, Genentech Inc., South San Francisco, CA, USA; Department of Infectious Diseases and Host-Microbe Interactions, Genentech Inc., South San Francisco, CA, USA; Department of Structural Biology, Genentech Inc., South San Francisco, CA, USA; Department of Infectious Diseases and Host-Microbe Interactions, Genentech Inc., South San Francisco, CA, USA; Department of Neuroscience, Genentech Inc., South San Francisco, CA, USA; Department of Infectious Diseases and Host-Microbe Interactions, Genentech Inc., South San Francisco, CA, USA; Department of Cancer Immunology, Genentech Inc., South San Francisco, CA, USA; Department of Human Pathobiology and OMNI Reverse Translation, Genentech Inc., South San Francisco, CA, USA; Department of Infectious Diseases and Host-Microbe Interactions, Genentech Inc., South San Francisco, CA, USA; Department of Bioinformatics, Genentech Inc., South San Francisco, CA, USA; Department of Infectious Diseases and Host-Microbe Interactions, Genentech Inc., South San Francisco, CA, USA

**Keywords:** Microbiome, mucosa, biopsy, *Clostridioides difficile*

## Abstract

**Background and Aims:**

This study aimed to identify microbial drivers of inflammatory bowel disease [IBD], by investigating mucosal-associated bacteria and their detrimental products in IBD patients.

**Methods:**

We directly cultured bacterial communities from mucosal biopsies from paediatric gastrointestinal patients and examined for pathogenicity-associated traits. Upon identifying *Clostridium perfringens* as toxigenic bacteria present in mucosal biopsies, we isolated strains and further characterized toxicity and prevalence.

**Results:**

Mucosal biopsy microbial composition differed from corresponding stool samples. *C. perfringens* was present in eight of nine patients’ mucosal biopsies, correlating with haemolytic activity, but was not present in all corresponding stool samples. Large IBD datasets showed higher *C. perfringens* prevalence in stool samples of IBD adults [18.7–27.1%] versus healthy controls [5.1%]. *In vitro*, *C. perfringens* supernatants were toxic to cell types beneath the intestinal epithelial barrier, including endothelial cells, neuroblasts, and neutrophils, while the impact on epithelial cells was less pronounced, suggesting *C. perfringens* may be particularly damaging when barrier integrity is compromised. Further characterization using purified toxins and genetic insertion mutants confirmed perfringolysin O [PFO] toxin was sufficient for toxicity. Toxin RNA signatures were found in the original patient biopsies by PCR, suggesting intestinal production. *C. perfringens* supernatants also induced activation of neuroblast and dorsal root ganglion neurons *in vitro*, suggesting *C. perfringens* in inflamed mucosal tissue may directly contribute to abdominal pain, a frequent IBD symptom.

**Conclusions:**

Gastrointestinal carriage of certain toxigenic *C. perfringens* may have an important pathogenic impact on IBD patients. These findings support routine monitoring of *C. perfringens* and PFO toxins and potential treatment in patients.

## 1. Introduction

Inflammatory bowel disease [IBD] is multifactorial with evidence linking genetic susceptibility, intestinal microbiota, and environmental factors as important contributors to pathogenesis of the disease.^1,2^ The intestinal microbiota has long been suspected to play a key role in IBD, with dysbiosis of mucosal-associated bacteria compared to healthy controls.^3,4^ While no specific single causative agent or mechanism is known, a number of bacterial species are linked to IBD and/or worsening of disease outcomes, including *Clostridioides difficile*, adherent-invasive *Escherichia coli*, *Mycobacterium avium* subspecies *paratuberculosis*, and *Fusobacterium nucleatum*.^3^

Symptoms of IBD, including blood in the stool and abdominal pain,^5^ may be related to host–microbial interactions. Blood in stool is an important biomarker of disease severity, and the faecal occult blood test is standard clinical practice.^6^ Red blood cells [RBCs] contain iron, an essential and typically limited microbial nutrient, at high concentrations.^7^ In an environment where nutrients are limited and competition is high, bacterial strains with haemolytic activity may have a competitive advantage in a diseased bleeding gut. Many pathogens, including *Staphylococcus aureus*, *Streptococcus pyogenes*, *Clostridium perfringens*, and certain *Bacillus*, encode haemolysins, which are important virulence factors.^8^ Additionally, the gut is densely innervated with sensory neurons, which regulate pain, a hallmark in many inflammatory conditions,^9^ including IBD.^5^ In response to toxins or harmful stimuli, nociceptor neurons—specialized spinal peripheral sensory neurons—mediate pain and other aversive behaviours.^10^ Recently, bacterial pore-forming toxins from *Staphylococcus aureus* and *Streptococcus pyogenes* were shown to trigger pain in mice through direct activation of spinal TRPV1+ nociceptor neurons.^11,12^

*C. perfringens* are Gram-positive, spore-forming, toxigenic anaerobic bacteria that commonly cause food poisoning and gastrointestinal illness.^13^ Though *C. perfringens* is well established in causing a variety of illnesses in both humans and animals, including acute food poisoning, gas gangrene soft tissue infections,^14^ and necrotizing enterocolitis in preterm infants^15^ [and chickens and horses^14^], only a few studies have associated this bacterial species with IBD.^16–19^*C. perfringens* has been associated with higher prevalence in paediatric IBD patients,^16^ poorer treatment outcomes in patients requiring a colectomy,^16–18^ and increased likelihood of developing pouchitis, a common ulcerative colitis [UC] surgery complication.^19^ Taken together, these studies demonstrate a correlation between presence of *C. perfringens* and unfavourable outcomes in individuals with IBD.

With the goal of identifying and isolating detrimental bacterial strains in IBD patients for further examination, we obtained mucosal biopsies and stool samples from a group of at-risk [undergoing a diagnostic colonoscopy] and early-stage [less than 10 years from diagnosis] paediatric IBD patients. Paediatric patients with IBD often present with more extensive and clinically severe disease than adult patients with IBD.^20^ We sequenced the samples, cultured their bacterial communities, and tested for haemolytic activity, finding *C. perfringens* present in all five haemolytic communities. Five unique *C. perfringens* strains were isolated from five patients and characterized for genome content, toxicity profile, and immune response. In addition to lysing RBCs, *C. perfringens* supernatants were toxic to a variety of cell types found in intestinal tissue, including endothelial cells, neutrophils, and neuroblasts, while colonic epithelial cells were less affected. Our work suggests *C. perfringens* may be a key member in many microbial communities that exacerbate IBD symptoms by causing cytotoxicity and abdominal pain through neuronal activation.

## 2. Materials and Methods

For further details, see Supplementary Information. Ethics approval: this research was reviewed and approved by the UCSF Institutional Review Board [IRB]. All participants were provided written informed consent.

### 2.1. Study design and patient sample culturing

A prospective study of patients aged 6 years and older undergoing oesophagogastroduodenoscopy [OGD] and colonoscopy as part of clinical care at UCSF Benioff Children’s Hospital Mission Bay was conducted. Patients were prepared for a standard endoscopy with MiraLAX and Dulcolax. During the procedure, two mucosal biopsies were obtained according to standard clinical practice from each of the duodenum, ileum, and colon and placed in Anaerobic Tissue Transport Media [Anaerobe Systems AS-919]. When present, biopsies were obtained from diseased areas of the colon with each location noted in Supplementary Table 1. Pre-biopsy colonic phosphate buffer wash (stored in BIOME-Preserve Anaerobic Microbiome Collection tubes [Anaerobe Systems]), and stool (stored in OMNIgene-GUT OMR-200 tubes [DNA Genotek]) within 1–3 days before the procedure were also collected. Samples were transported within 4 h of the procedure and processed—culturing the biopsies and freezing the remaining samples. Biopsies were transferred to 1–2 mL phosphate-buffered saline [PBS] in a gentleMACS M-tube [Miltenyi Biotec], sealed tightly, removed from the anaerobic chamber, and homogenized using a gentleMACS Dissociator [Miltenyi Biotec]. The tubes were then brought back into the anaerobic chamber and processed for culturing. Bacterial culturing was with 1 mL media in 2-mL 96-well Polypropylene Deep Well Plates [Corning 3960], adding ~50 µL of homogenized biopsy slurry. Plates were sealed with AeraSeal films [Excel Scientific BS-25] and incubated at 37°C anaerobically. After 2–3 days, the plates were passaged by transferring 10 µL to 1 mL of fresh media, then grown for an additional 2–3 days. After the second growth passage, cultures were additionally processed as supernatant plates for *in vitro* assays.

### 2.2. *C. perfringens* culturing and genetics

We selected cultures with highest *C. perfringens* relative abundances for colony plating on tryptose sulphite cycloserine [TSC] plates and used conditions to select for spores, including aerobic ethanol treatment^21^, to obtain bacterial strain isolates [Supplementary Table 10]. Insertional mutants were generated using the Group II intron system, or ‘ClosTron’, developed previously,^22,23^ containing a 309-bp variable intron targeting region [Supplementary Table 12]. Polymerase chain reaction [PCR] was performed using Q5 Hot Start Master Mix [New England BioLabs, M0494], Gibson assemblies using NEBuilder HiFi DNA Assembly mix [New England BioLabs, E2621], and plasmid cloning was in *Escherichia coli* TOP10 chemically competent cells [Invitrogen]. Plasmids were transformed by electroporation into *E. coli* CA434 for conjugation. Overnight cultures of the donor *E. coli* and recipient *C. perfringens* were mixed in a 2:1 ratio, spun down, and spotted on a tryptic soy broth [TSB] plate for 24–48 h conjugation at 37°C anaerobically.

### 2.3. DNA extraction and 16S_V4 sequencing, data processing, and analysis

Microbial DNA was extracted from ~200 µL of culture using DNeasy QIACubeHT Powersoil Pro [Qiagen] for Patients 1–3 or DNeasy 96 PowerSoil Pro [Qiagen] for Patients 4–9. Biopsies were directly extracted using the single-tube QIAamp PowerFecal Pro DNA kit [Qiagen]. Extracted DNA was used for 16S_V4 PCR using barcoded reverse primers.^24^ PCR was done with Takara Ex Taq DNA Polymerase, Hot Start version [RR006B], and Roche BSA [#10711454001] as previously described.^25^ 16S libraries were pooled and sequenced following Illumina’s metagenomics workflow. QIIME v2019.7 was used to process the 16S-V4 rRNA gene sequence data as previously described.^26,27^ Taxonomy was assigned based on the V4 region of the Genome Taxonomy Database 16S rRNA gene sequence database [r202].^28,29^ β-Diversity measures were calculated in QIIME v2019.7. All statistical analyses were conducted in the R statistical environment [R Core Team, 2020.]. Bray–Curtis distance matrices were visualized via non-metric multidimensional scaling [NMDS] and permutational multivariate analysis of variance [PERMANOVA] was used to determine relationships between metadata [e.g. sample location] and bacterial microbiota composition.

### 2.4. Whole-genome sequencing

For Oxford Nanopore use, genomic DNA was sequenced either individually on MinION flow cells or multiplexed on PromethION flow cells. For library preparation, the Ligation Sequencing Kit [Oxford Nanopore Technologies, SQK-LSK109] was used with an input of 1 µg of genomic DNA. Sample multiplexing was performed using 500 ng of end-repaired DNA with the Native Barcoding Expansion Kit [Oxford Nanopore Technologies, EXP-NBD104]. For Illumina library preparation, the Nextera DNA Flex kit [Illumina] was used with an input of 100 ng of genomic DNA. The resulting libraries were multiplexed and sequenced on NovaSeq [Illumina] to generate 5 million paired-end 75-bp reads for each sample.

### 2.5. Red blood cell lysis assays

Whole blood from healthy subjects was collected via Genentech’s internal donor programme, Samples for Science, following the approved IRB protocol [20080040] study specifications. Bacterial supernatants, accounting for 10% of the final solution, were mixed with 2% whole blood in PBS to a final volume of 200 µL in a 96-well plate. Plates were incubated at 37°C for the specified amount of time, and for 4 h for single point readings. At each time point, 50 µL of supernatant was removed and mixed with 50 µL PBS. The 100% lysis control was 0.5% Triton X-100. Absorbance at 450 nm was subtracted from that at 650 nm.

### 2.6. Toxin purification, protein gels, Western blots

Toxins were expressed with 6xHis and SUMO cleavage tags in *E. coli* grown in Terrific Broth. Cells were resuspended in lysis buffer [50 mM Tris pH 7.5, 300 mM NaCl, 15% glycerol, 5 mM imidazole, 5 mM MgCl_2_, 1 mM TCEP [Tris(2-carboxyethyl)phosphine]] with Roche cOmplete Ultra EDTA-free protease inhibitor [5892970001] and sonicated. Lysates were incubated with AmMag Ni resin magnetic beads [Genscript L00776], with 20 mM imidazole for washes and 250 mM imidazole for elution. Proteins were further purified by size exclusion chromatography at 4°C with a Superdex 200 Increase 10/300 GL column [GE Healthcare] and 20 mM HEPES pH 8.0 with 150 mM NaCl on an AKTA pure [Cytiva]. Protein SDS-PAGE was run with 10% Bis-Tris gels [Invitrogen] with MOPS running buffer. Western blots were run using Invitrogen’s iBlot 2 system: nitrocellulose membrane with primary anti-PFO [perfringolysin O] polyclonal antibody [Invitrogen PA5-117550, 3 mg/mL, rabbit IgG] at 1:2500 and secondary anti-rabbit [Li-Cor IRDye 800CW, 1 mg/mL] at 1:5000. Commercial recombinant PFO protein [ATCC BTX-100] was used for comparison.

### 2.7. PCR and sequencing analysis of biopsy RNA

Biopsy slurries in PBS were extracted using the AllPrep PowerViral DNA/RNA kit [Qiagen] with an added on-column DNase I [Qiagen] step before the final wash and elution. RNA was further treated with DNase using the TURBO DNA-free kit [Invitrogen AM1907] to ensure genomic DNA removal prior to cDNA preparation by iScript Reverse Transcription [Bio-Rad]. Both final cDNA and pre-reverse transcription RNA were used as templates to run PCR analysis with Q5 Hot Start mix: 8-µL reactions with 0.8 µL template and 500 nM of each primer [Supplementary Table 11]. The PCR program was thermocycling: 98°C for 2 min, 40 cycles [98°C for 10 s, 58°C for 30 s, 72°C for 30 s], 72°C for 10 min, then hold at 4°C. The first PCR was followed by a second using 0.8 µL of the first PCR as template. Reactions were visualized on 4% agarose E-gels [Invitrogen], and positive hits were Sanger sequenced for verification.

### 2.8. CellTiter-Glo cell viability assays to estimate toxicity

Mammalian cells were cultured using standard techniques in respective growth media in T75 or T150 flasks. Cell lines [T84, HT29, Caco2, Neuro-2a] were grown from in-house stocks originally passaged from ATCC. T84 cells were grown in RPMI 1640 with 10% heat-inactivated fetal bovine serum [FBS, R&D Systems], 2 mM Glutamax and Pen-Strep. HT29, Caco-2, and Neuro-2a cells were grown in DMEM with 10% FBS, 2 mM GlutaMAX and 1× Pen-Strep [Gibco]. Human umbilical vein endothelial cells [HUVECs; PromoCell] were grown in Endothelial Cell Growth Media [PromoCell]. Cells were seeded at ~10 000 cells per well in white clear-bottomed tissue culture treated 96-well plates [Corning 3610] and grown for an additional 1–3 days depending on the cell type. For human colonic organoid-derived monolayers, organoids were derived from deceased adult Donor Network West donors. Organoids were grown in expansion media modified from previous protocols,^30^ dissociated to single cells and plated on Collagen IV [Sigma C5533] coated plates. Medium was swapped with low serum [0.25% FBS] or serum-free [for HUVECs] media, and incubated with test compounds at 4–10% overnight [16–24 h]. Cell viability was then measured using a CellTiter-Glo Luminescent Cell Viability Assay [Promega] according to kit instructions, with luminescence read out on a SpectraMax M5 plate reader at 500 ms integration time. Roche’s Cytotoxicity Detection Kit [LDH] was used according to kit instructions.

Human neutrophils were extracted from whole blood using the EasySep Direct Human Neutrophil Isolation Kit [StemCell Technologies]. Human peripheral blood mononuclear cells [PBMCs] were purchased from STEMCELL Technologies. PBMCs were incubated with compounds and the resulting supernatants submitted for Luminex cytokine analysis using the Bio-Rad 23-plus cytokine panel. Values below the detection range were set as zero.

For YO-PRO toxicity studies, cells were incubated at the same conditions as for CellTiter-Glo assays but with YO-PRO-1 dye [Invitrogen Y3603] at 1 µM, imaged every 20–30 min at 20× on an Incucyte S3 in phase contrast and green fluorescence channels [300 ms exposure, 441–481 nm excitation, 503–544 nm emission].

### 2.9. Neuron-activation assays

Neuro-2a cells were incubated in Dulbecco’s modified Eagle medium [DMEM] with 0.25% FBS [as used in toxicity assays] with YO-PRO-1 dye and test compounds added, and imaged every 5 min on an Incucyte S3 in phase contrast and green fluorescence channels. Mouse dorsal root ganglia [DRGs] were isolated from mice and processed as previously described.^31^ Mouse DRGs were incubated in DMEM with 10% FBS and pre-incubated with YO-PRO-1 dye for 20+ minutes before *C. perfringens* supernatant addition. Cells were then imaged every 5 min for 4 h in phase contrast and FITC channels with a Plan Apo 20× [NA 0.75, Nikon] objective on a Nikon TI-E perfect focus inverted microscope equipped with a Neo scMOS camera [Andor, Oxford Instruments], 37°C/5% CO_2_ environmental chamber [Okolab], and a SOLA V-NIR LED light source [Lumencor], all run by NIS Elements software [Nikon]. For purified toxin experiments, cells were imaged every 3 min for 2.5 h in phase contrast and 488-nm laser channels with a similar Nikon microscope setup equipped with a spinning disc confocal CSU-X1 [Andor, Oxford Instruments], ILE laser launch [Spectral Applied Research], and Prime 95B sCMOS camera [Teledyne Photometrics].

### 2.10. Prevalence estimate of *C. perfringens*

Using our previously described bioinformatics pipeline^32^ that performs sensitive taxonomy classification to a standardized database of bacterial and archaeal genomes, prevalence estimates of *C. perfringens* in Figure 3G were made on two independent healthy cohorts,^32,33^ four independent UC cohorts, and three independent CD cohorts [Supplementary Methods].

### 2.11. Data availability

Microbiome sequence data are available from The European Genome-phenome Archive [EGA, https://ega-archive.org/] under Study ID EGAS00001007538. The five *C. perfringens* bacterial genomes are available from NCBI under BioProject ID PRJNA1043401 with BioSample accessions SAMN38338936–40. The prevalence estimates of *C. perfringens* from healthy populations were calculated from taxonomy profiles derived from sources in the public domain [listed in Supplementary Table 6] and made available via the curatedMetagenomicData R package [v3.9.1],^37^ as well as metagenomic data from Zeevi *et al*.^33^ [European Nucleotide Archive, accession PRJEB11532] and Byrd *et al*.^32^ [European Genome-Phenome Archive, accession EGAS00001004437]. The abundance profiles of *C. perfringens* from IBD patients will be made available upon reasonable request.

## 3. Results

### 3.1. Paediatric gastroenterology patients’ biopsy cultures have haemolytic activity

**Figure 1.**
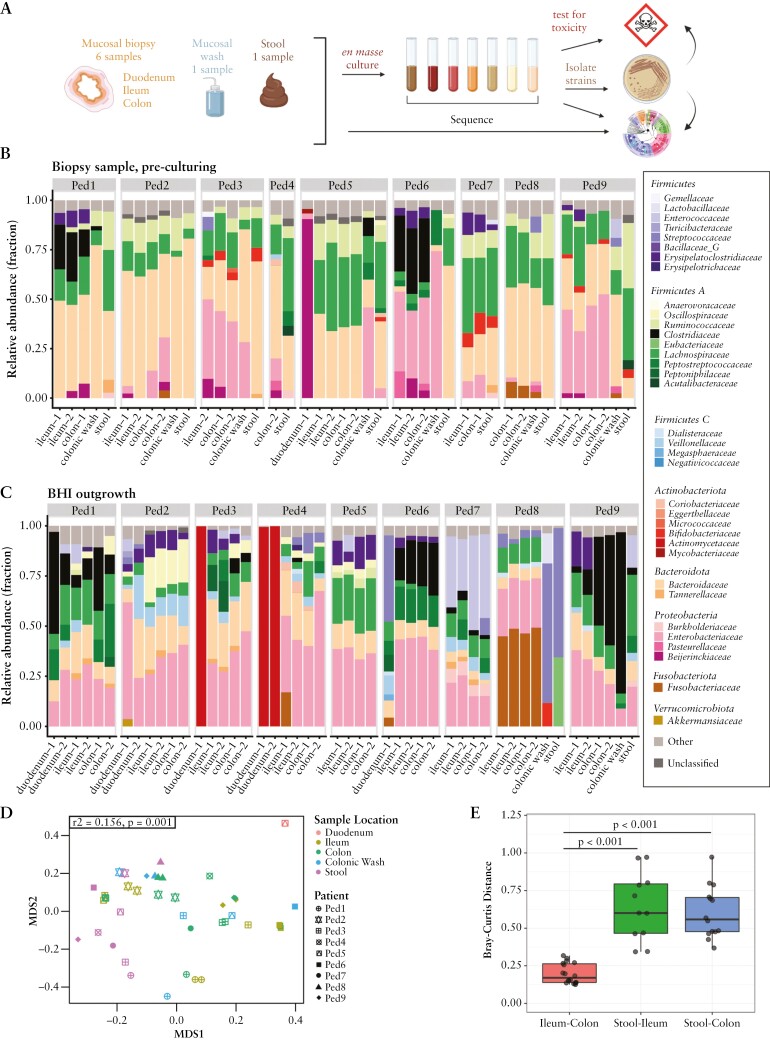
Sample collection, workflow, and sequencing analysis from paediatric gastroenterology patients. [A] Sample workflow diagram. Patient samples sequenced [B] directly as biopsies pre-culturing and [C] after the second passage outgrowth in BHI media. Samples were sequenced using 16S V4 sequencing and classified at the family taxonomic rank. Minimum 10 000 reads filtering cutoff per sample. [D] Beta diversity analysis of the microbiome from outgrowth communities for each patient sample: NMDS ordination plot of Bray–Curtis distances. Colour represents sample location, shape represents patient. PERMANOVA was performed to test the impact of sample location on Bray–Curtis distance, stratified by patient [*r*^2^ = 0.156, *p* = 0.001]. [E] Boxplot of pairwise Bray–Curtis distances between intrapatient ileum, colon, and stool outgrowth communities. The *p*-values were determined by Wilcoxon rank sum test.

Nine patients from the Pediatric Gastroenterology Clinic at UCSF Benioff Children’s Hospital [San Francisco, CA, USA] undergoing a colonoscopy and upper endoscopy were recruited for sample collection. Four were previously diagnosed with Crohn’s disease [CD] or UC, while the other five underwent diagnostic surveillance with diagnoses of two with CD, one irritable bowel syndrome [IBS], one colitis, and one not diagnosed with a gastrointestinal disorder [Supplementary Table 1]. Mucosal biopsy samples, representing close interactions between bacteria and mucosal tissue, were cultured in different microbial growth media, including brain heart infusion [BHI], to maximize recovery of microbial diversity [Figure 1A]. Media selection was informed by prior *ex vivo* batch culturing of human stool samples.^38^ Each outgrowth sample is referred to as a bacterial ‘community’, representing a mixture of taxa. While sample culturing inevitably introduces culture-based bias by enriching certain strains while depleting others from the original community, our objective was to acquire many human-derived isolates, rather than replicate native community structure.

We performed 16S V4 rRNA sequencing on the samples prior to and after culturing [Figure 1B and C; Supplementary Figures 1 and 2 and Supplementary Tables 2 and 3]. Prior to culturing, the microbiota communities differed significantly in composition based on sample collection sites [Figure 1D]. Mucosal-associated communities had similar microbiota composition to each other but differed significantly from the stool sample [Figure 1E], in accordance with the observations of others.^39–41^ To select a subset for downstream analysis, microbial communities grown in different media were evaluated for bacterial diversity, including total operational taxonomic units [OTUs] present in the sample, evenness, and Shannon diversity [Supplementary Figure 3]. We selected BHI as the base medium for downstream analysis based on its desirable profile, including a high number of OTUs, evenness across the cultures, and high Shannon diversity.

**Figure 2.**
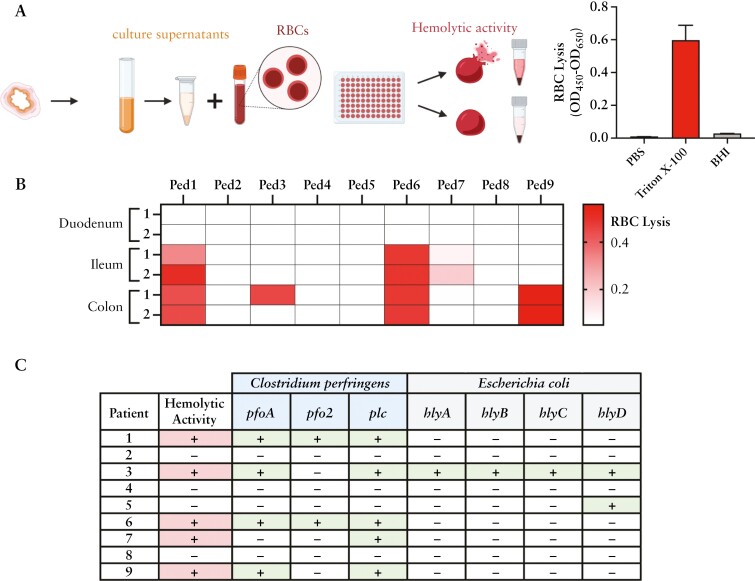
Patient biopsy community samples are haemolytic. [A] Experimental schematic for RBC lysis, measured as [OD_450_ − OD_650_]. Media control [BHI] and 100% lysis control [0.5% Triton X-100] are shown. [B] Biopsy outgrowth in BHI after the second passage for each patient was measured for RBC lysis. Two biopsies from each location were evaluated. [C] Long-read metagenome-assembled genomes [MAGs] of BHI outgrowth communities were searched against the virulence factor database [VFDB]. Presence of sequences homologous to known haemolysin genes is indicated with ‘+’ and absence with ‘-’. Haemolytic activity from samples outgrown in BHI is noted with ‘+’ and no activity with ‘-’. The genes *pfoA*, *pfo2*, *plc*, and *hlyA* are predicted haemolysins, and *hlyB–D* haemolysin accessory genes. Primary haemolytic data available in Supplementary Table 5.

IBD patients experience intestinal bleeding, which is uncommon in healthy individuals. We measured the microbial community supernatants for haemolytic activity [Figure 2A], which could potentially offer a competitive fitness advantage. Of the nine patients, five had outgrowth community supernatants that exhibited haemolytic activity [Patients 1, 3, 6, 7, and 9] [Figure 2B]. Four of those five patients had more than one haemolytic community derived from the mucosa biopsies, suggesting the presence of haemolytic strains across the gastrointestinal [GI] tract.

To identify putative haemolytic toxin genes, we sequenced a single mucosal community grown in BHI from each patient using long-read whole metagenome sequencing [WMS]. Draft assemblies were compared to the virulence factor database [VFDB] to identify evidence of homology to known virulence factors from bacterial pathogens^42,43^ [Supplementary Table 4]. Sequences homologous to known haemolysins were detected in all five haemolytic communities [Figure 2C] and came from two species: *C. perfringens* and *E. coli*. Homology to *hlyA*, limited to hypervirulent strains of *E. coli*,^44^ was present in only one haemolytic community [Figure 2C]. In contrast, homology to the *C. perfringens* alpha-toxin gene [*plc*: phospholipase C], encoding a well-characterized haemolysin, was found in all five haemolytic communities, along with the haemolysins perfringolysin O gene [*pfo*] in four, and the alveolysin gene [*pfo2*] in two communities.^45^ The universal presence of *C. perfringens* within the haemolytic BHI communities and high incidence amongst the paediatric patients prompted further characterization.

### 3.2. *C. perfringens* was present within microbial communities with haemolytic activity

**Figure 3.**
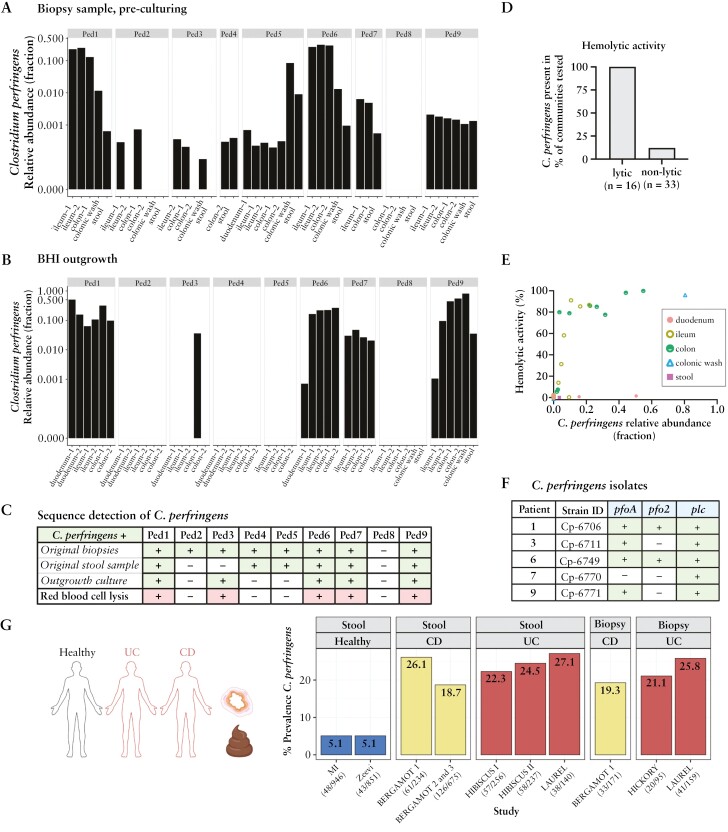
*C. perfringens* is present in biopsies, haemolytic communities, and enriched in IBD patients. 16S V4 sequencing identified *C. perfringens* abundance in patient samples [A] prior to outgrowth and [B] after outgrowth of the second passage in BHI. [C] *C. perfringens* detected [>0.001%] in biopsies [top row], stool samples [second row], outgrowth cultures [biopsy or stool], and haemolytic activity from outgrown cultures [biopsy or stool]. Haemolytic activity was defined by bacterial community supernatants grown in BHI that lysed RBCs with OD_450_ − OD_650_ > 0.1 after 4 h. [D] Haemolytic activity shown graphically for BHI grown communities containing >0.001% *C. perfringens* [Supplementary Table 5]. [E] Haemolytic activity vs *C. perfringens* relative abundance. [F] *C. perfringens* bacterial isolates had diverse haemolysin gene content. [G] Prevalence of *C. perfringens* in healthy Milieu Intérieur [MI]^32^ and non-diabetic Israeli [Zeevi]^33^ cohorts and IBD subjects enrolled in etrolizumab phase III clinical trials,^34–36^ as measured from baseline stool or biopsy samples.

Prior to culturing, *C. perfringens* was detected by 16S V4 rRNA in the stool, colonic wash, and/or biopsy samples in eight of nine patients [Figure 3A and C; Supplementary Table 5]. Patients 1 and 6 had >20% *C. perfringens* relative abundance in biopsies but <1% in stool. For Patients 2 and 3, *C. perfringens* was detected in the biopsy samples but not the corresponding stool samples. Furthermore, *C. perfringens* detection in stool was only evident when also detected in biopsy samples. This suggests mucosal samples are comparatively enriched for *C. perfringens*.

After culturing, *C. perfringens* was detected by sequencing in communities from five patients [Figure 3B and C; Supplementary Figure 4 and Supplementary Table 5]. The presence and abundance of *C. perfringens* is consistent with haemolytic activity [Figure 3C–E]. In particular, only one of two colonic biopsies from Patient 3 outgrew *C. perfringens*, and only that community had haemolytic activity despite most other taxa being similar between the biopsies [Supplementary Figure 5]. Similarly, Patient 9 bacterial communities grown from biopsies had stronger haemolytic activity and higher *C. perfringens* relative abundance compared to the stool-grown sample [Supplementary Figure 6]. In a limited number of communities, the presence of *C. perfringens* was detected but haemolytic activity was not observed [Figure 3D]. This may be due to low abundance, low levels of secreted toxin, or indirect mechanisms to inhibit haemolytic activity. Haemolytic activity was common when *C. perfringens* was present.

Expanding from this small cohort of paediatric patients, we evaluated *C. perfringen*s prevalence in adult healthy and IBD populations in large datasets. In healthy adults, *C. perfringens* was found in 5.07% [French *Milieu Intérieur* Consortium^32,46^] and 5.05% [Israeli non-diabetic population^33^] of baseline stool samples. Analysing 77 additional curated public datasets^37^ with 13 827 stool samples from ‘healthy’ or ‘control’ adults [Supplementary Table 6], *C. perfringens* prevalence in adults aged 18–65 years was 4.5% across available samples [Supplementary Figure 7]. In contrast, in adults with moderate to severe UC or CD enrolled in large, multicentre phase 3 clinical trials, baseline prevalence of *C. perfringens* was 18.7–27.1% in stool and 19.3–25.8% in biopsy samples [Figure 3G]. This higher prevalence in IBD samples is probably not due to dataset biases as samples were obtained globally and processed at multiple vendors, which included methods to incorporate vigorous lysing steps to lyse bacteria resistant to chemical lysis [Gram-positive bacteria and spores]. Analysis of publicly available curated IBD WMS datasets revealed *C. perfringens* abundance and prevalence varied between studies with a trend of higher prevalence in IBD patients [Supplementary Figure 7C and D]. These findings indicate *C. perfringens* is more prevalent in IBD adult patients than in healthy subjects, suggesting an association with IBD pathology.

### 3.3. Isolated *C. perfringens* strains are haemolytic and encode numerous virulence factors

We successfully isolated *C. perfringens* from five patients and performed whole genome sequencing [Supplementary Figure 8, Supplementary Tables 7 and 8]. Notably, each isolate had a unique genome and was assigned a unique strain identifier [e.g. Cp-6706]. Each genome was compared to the VFDB to identify homology to known virulence factors involved in adhesion, exotoxin production, and host extracellular matrix degradation.^42,43^ Homology to ten virulence factors was observed across all five strains [Supplementary Table 9]. Notably, no homology to genes for necrotizing toxin [*beta2*-toxin, *netB*], enterotoxin [*cpe*], iota toxin, epsilon toxin [*etx*], or mu toxin was found. Thus, all isolates are putatively toxinotype A, the most ubiquitous strain type found in the environment and occasionally linked to foodborne outbreaks.^47–49^ Core genome-based phylogenies were constructed using the isolates and publicly available genome assemblies.^45^ The five *C. perfringens* strains belong to three distinct clades [Supplementary Figure 9].

**Figure 4.**
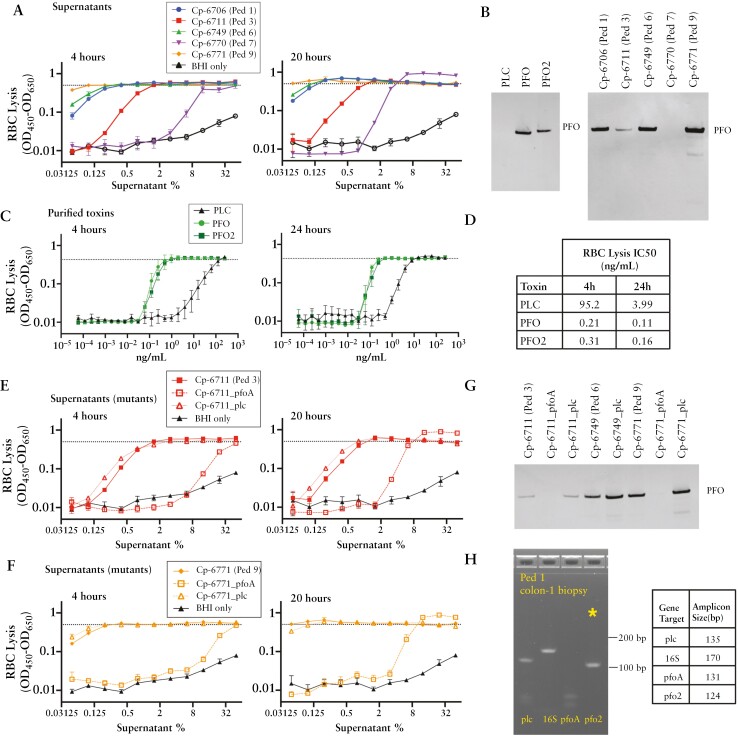
Variability in haemolytic activity of *C. perfringens* isolate supernatants. [A] RBC lysis activity measured from the supernatants of isolated *C. perfringens* strains grown overnight in BHI anaerobically for 4 h [left] and 20 h [right]. RBC lysis was measured as [OD_450_ − OD_650_]. Dotted lines represent lysis with 0.5% Triton X-100 as the 100% lysis control. Three biological replicates were evaluated. [B] Western blot probing for PFO protein levels from purified toxins [left, 2.5 µg/mL] or *C. perfringens* supernatants [right]. [C] RBC lysis activity measured from purified haemolysins, PFO, PFO2, and PLC after 4 h [left] and 24 h [right]. [D] Half maximal inhibitory concentrations [IC50] calculated at both 4 and 24 h for RBC lysis activity. [E, F] RBC lysis activity measured from supernatants of *C. perfringens* [E] Cp-6711 and [F] Cp-6771 and isogenic toxin mutants at 4 h [left] and 20 h [right]. Three biological replicates are shown. [G] Western blot analysis of matched supernatants from *C. perfringens* isogenic toxin mutants probing for PFO protein levels. Full Western blots are shown in Supplementary Figure 10. [H] Agarose DNA gel of PCR analysis of Patient 1 colon-1 biopsy cDNA from extracted RNA. Indicated genes probed with primer sets, with *pfo2* gene marked with an asterisk [*]. Full DNA gels are shown in Supplementary Figure 11.

Upon incubation with RBCs, every isolated *C. perfringens* strain demonstrated haemolytic activity [Figure 4A]. Cp-6770 required a higher concentration of supernatant to induce RBC lysis and encoded *plc* but neither *pfoA* nor *pfo2*, two toxin genes associated with haemolytic activity. Conversely, strains with the highest haemolytic activity all encoded *pfoA*. Cp-6706 and Cp-6749 also encoded *pfo2* in their genomes [Figure 3F]. To evaluate toxin production of each strain, we used a polyclonal antibody raised against PFO and measured protein levels in the supernatant. At 86% protein identity, both PFO and PFO2 proteins were detected by the antibody [Figure 4B], and measured total protein levels correlated with haemolytic activity [Figure 4A and B]. PFO protein sequences across the five strains shared >98% identity.

To directly compare the haemolytic activity of each toxin, phospholipase C [PLC], PFO, and PFO2 from Cp-6706 were expressed in *E. coli*, purified, and assessed for haemolytic activity [Figure 4C; Supplementary Figure 10]. As expected, each toxin was sufficient for haemolytic activity.^50,51^ PFO and PFO2 displayed similar activities with an IC50 at 4 h of 0.21 and 0.31 ng/mL, respectively, while PLC had an IC50 of 95.2 ng/mL, >300-fold less potent [Figure 4D]. To confirm these toxins were necessary for the strains’ haemolytic activity, we generated *C. perfringens* genetic insertion mutants using a Group II intron system.^22,23^ Disruption of *pfoA* in both Cp-6711 and Cp-6771 greatly reduced haemolytic activities, while disruption of *plc* did not [Figure 4E–G]. The results demonstrate PFO is the primary contributor to haemolytic activity observed in these strains.

To assess whether *C. perfringens* was synthesizing toxins in the mucosal region, we performed PCR analysis on cDNA of RNA extracted from biopsies with the highest *C. perfringens* relative abundances. Despite biopsy samples not being specifically preserved for subsequent RNA analysis and having unknown masses, both *plc* and *pfo2* signatures were found in Patient 1 colon biopsy [Figure 4H], suggesting active transcription of toxins at the mucosal site. Ileum biopsies from Patients 1 and 6 were also *pfo2*-positive, and biopsies analysed also had *plc* signatures [Supplementary Figure 11]. All biopsies had 16S rRNA, and the amplicons were verified through Sanger sequencing, with *pfo2* and *plc* showcased [Supplementary Figure 12].

### 3.4. *C. perfringens* supernatants and toxins were cytotoxic to several intestinal cell types

**Figure 5.**
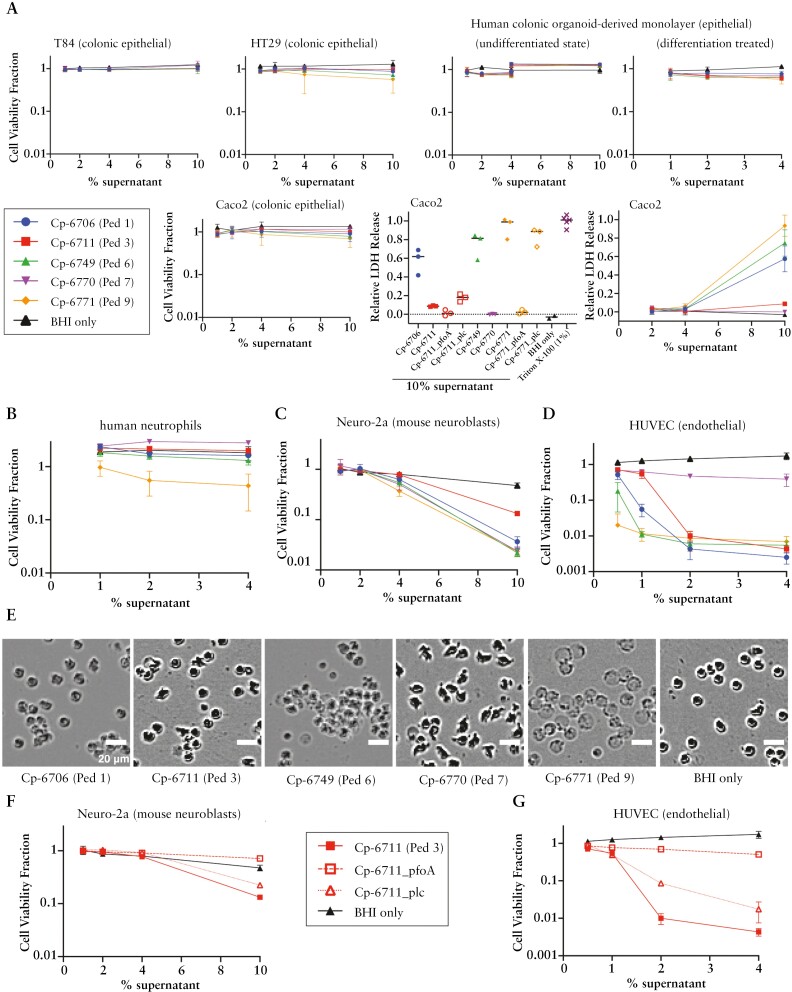
Toxicity of *C. perfringens* supernatants on select cell types. *C. perfringens* isolate supernatants grown in BHI media were incubated with different cells for 20–24 h and measured for viability based on ATP amount [CellTiter-Glo assay]. The cell viability fraction from the CellTiter-Glo assay was calculated by normalizing to the PBS-only control for each cell line. The relative lactate dehydrogenase [LDH] release was normalized to the 100% lysis control [0.5% Triton X-100]. [A] Epithelial cells, [B] isolated human neutrophils, [C] mouse neuroblast cell line Neuro-2a, and [D] human umbilical vein endothelial cells [HUVECs] are shown. Three replicates are shown. [A] Adult colonic organoid-derived monolayer epithelial cells in the undifferentiated state were tested from two adult donors, at 1–4% and 4–10% supernatants. Caco2 cells were additionally incubated with supernatants for 2 h and evaluated for LDH release. [B] Human neutrophils are two replicates each on two patient purifications [four replicates in total]. [E] Representative phase contrast images of human neutrophils with *C. perfringens* supernatants and BHI media only. Scale bar = 20 µm. [F, G] Bacterial supernatants from *C. perfringens* toxin mutants grown in BHI were incubated with [F] Neuro-2a and [G] HUVECs for ~24 h and measured for viability by CellTiter-Glo. Three biological replicates are shown.

To gain more understanding of *C. perfringens* strain toxicity, we broadened our characterization to assess their impact on cell viability to a variety of cell types found in the GI tract. Exposing three colonic epithelial cell lines and adult colonic organoid-derived monolayer epithelial cells^52^ to *C. perfringens* supernatants, we observed minimal viability losses for the majority of conditions [Figure 5A]. However, more visible damage was measured from select supernatants, notably treatment with Cp-6771 at the highest concentration [10%], which led to an ~40% decrease in HT29 cell viability. Using an alternative method to measure cell death, lactate dehydrogenase [LDH] release was quantified from Caco2 cells incubated with 10% bacterial supernatants for 2 h. This resulted in high LDH release from three strains, consistent with previous observations in *pfoA*-harbouring strains [Figure 5A].^15^ Incubating with 4% supernatants had minimal effects on LDH release, demonstrating high concentrations were needed to elicit the response. Cp-6711 and Cp-6771 insertion mutants showed greatly reduced LDH release from *pfoA* disruption but not from *plc* disruption.

Although LDH release indicated epithelial damage during the 2-h incubation period, ATP-based measurements after >20 h showed less impact, suggesting cells maintain viability across a population after initial injury. To investigate this process, we used YO-PRO green fluorescent dye, a nucleic acid stain typically excluded from viable cells, to monitor cell permeability, which arises after the formation of membrane pores. While not all cells were dye-permeable, cells appeared to contract at the highest concentrations of 10% supernatants [Supplementary Figure 13 and Supplementary Video 1]. Even after shrivelling, many cells still were not dye-permeable, and the ATP readings suggest population viability. Insertion mutants of *pfoA* reduced YO-PRO dye uptake, whereas *plc* mutants maintained or had higher dye amounts in cells [Supplementary Figure 13C].

To compare with Caco2 cells, we imaged adult colonic organoid-derived monolayer epithelial cells in the undifferentiated state using YO-PRO dye. In contrast to Caco2 cells, these organoid-derived cells were resistant against 10% supernatants and purified toxins, maintaining consistent cell coverage and minimal dye uptake over time [Supplementary Figure 14 and Supplementary Video 2]. Together, the epithelial toxicity experiments showed colonic organoid-derived monolayer epithelial cells and select cell lines demonstrate resilience to *C. perfringens* factors, while the Caco2 line may exhibit specific sensitivity.

In contrast to epithelial cells, cells that typically lie beneath the epithelial barrier, such as endothelial cells, neuroblasts, and neutrophils, measured substantially larger drops in ATP-based viability in a dose-dependent manner [Figure 5B–D]. HUVECs were very sensitive to *C. perfringens* supernatants. Cp-6770 caused the least amount of damage to HUVECs, while Cp-6771 was generally the most toxic across multiple cell types and was the only strain that reduced the viability of neutrophils. Cp-6771 also had the strongest haemolytic activity of the five isolates [Figure 4A]. Microscopy images of human neutrophils treated with *C. perfringens* supernatants revealed cell morphology changes including membrane puckering and blebbing, despite similar measurements in viability to the media control [Figure 5B]. After treatment with Cp-6771 supernatants, neutrophils lost phase contrast density, transforming into ghost cells and losing viability [Figure 5E]. The Cp-6711 *plc* and *pfoA* insertion mutants had reduced cell toxicity with both Neuro-2a mouse neuroblasts and HUVECs [Figure 5F and G]. The *pfoA* mutant nearly abolished toxicity in both cell lines, while the *plc* mutant showed minimal effects in Neuro-2a cells and partial reduction in HUVECs. These data suggest these PFO toxins are the primary drivers of cell toxicity and haemolytic activity in the *C. perfringens* supernatants.

To see how the *C. perfringens* toxicity profiles compared to other species typically found in the GI tract, we assessed 75 diverse bacterial strain supernatants, representing the major phyla of the human gut microbiome,^53^ for cytotoxicity to HT29 epithelial cells, human PBMCs, HUVECs, and RBCs [Supplementary Figure 15]. Cells remained >50% viable in the presence of all bacterial supernatants tested except *C. perfringens*, emphasizing *C. perfringens* cytotoxicity as a rare trait compared to numerous bacterial species typically found in the healthy human gut.

**Figure 6.**
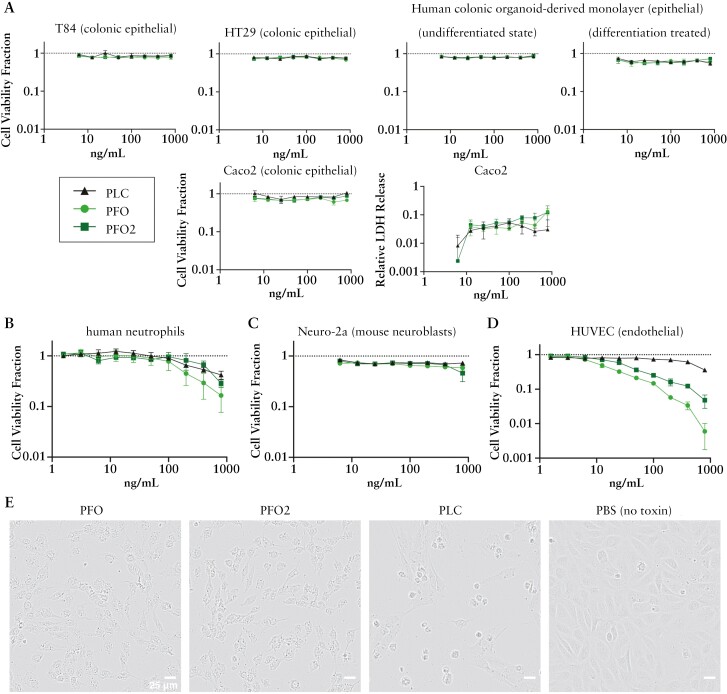
Toxicity of purified *C. perfringens* toxins on selected cell types. Purified *C. perfringens* toxins were incubated with different cells for 20–24 h and measured for viability based on ATP amount [CellTiter-Glo]. The cell viability fraction was calculated by normalizing to the PBS-only control for each cell type [no toxin, dotted lines = 1]. [A] Epithelial cells, [B] isolated human neutrophils, [C] mouse neuroblast cell line Neuro-2a, and [D] human umbilical vein endothelial cells [HUVECs] are shown. Three biological replicates are shown. [E] Representative phase contrast images of HUVECs incubated with 800 ng/mL of each indicated toxin for 23 h. Scale bar = 25 µm.

We next assessed whether purified *C. perfringens* toxins alone could induce cell toxicity. Similar to the findings from the supernatant experiments, the epithelial cells showed minimal decreases in viability. However, human neutrophils and HUVECs were sensitive to high concentrations [~800 ng/mL] of PLC, PFO, and PFO2, while Neuro-2a cells showed relatively milder effects [Figure 6A–D]. While HUVECs were sensitive to high concentrations of haemolysins, the amount required for toxicity was >500-fold greater than the <1 ng/mL needed to lyse RBCs [Figure 4D]. The morphology of HUVECs challenged with the three toxins displayed two phenotypes: treatment with either PFO or PFO2 resulted in cell granulation with cells continuing to stretch out, while treatment with PLC resulted in cells contracting, balling up, and granulating [Figure 6E], reinforcing the different toxin mode-of-actions described previously.^54^

Since IBD is characterized by an imbalance of pro-inflammatory and anti-inflammatory cytokines, we next investigated strain effects on human PBMCs to profile elicited immune responses.^55^ Supernatants from the five *C. perfringens* strains plus eight additional *Clostridium* species isolated from humans were profiled for their cytokine response [Supplementary Figure 16]. PBMC viability was unaffected after 24 h of incubation except when incubated with supernatants from three *C. perfringen*s strains: Cp-6706, Cp-6749, and Cp-6771. Common chemokines and cytokines induced across the different *Clostridium* species were CCL20, IL-12, IL-13, and IL-21. None of the *C. perfringens* led to GM-CSF nor IL-17E production despite responses from the other *Clostridium* species, while all *C. perfringens* induced IL-17A and IL-9 production, with little to no levels detected from the other *Clostridium* species.

### 3.5. *C. perfringens* supernatants activate sensory neurons

**Figure 7.**
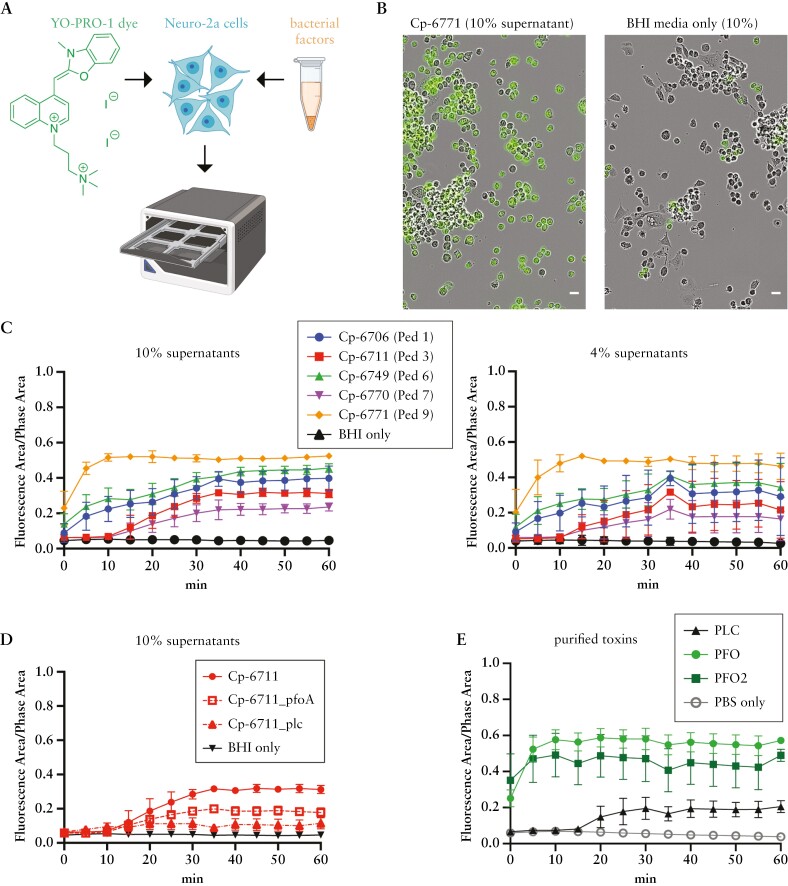
*C. perfringens* supernatants and purified toxins activate mouse neuroblasts. [A] Experimental setup: Neuro-2a [mouse neuroblastoma] cells were preloaded with the cell permeability fluorescent marker YO-PRO for live imaging. Individual *C. perfringens* strains were grown overnight in BHI media, and bacterial supernatants were added as indicated. Images were collected every 5 min. [B] Representative images of Neuro-2a cells incubated with Cp-6771 supernatant or BHI media only at *t* = 0. Green fluorescence is YO-PRO uptake. Scale bar = 25 µm. [C] The fraction of fluorescent cells, calculated from total areas, is displayed from three biological replicates and two image locations within a single well. Shown are cells treated with final concentrations of 10% and 4% bacterial supernatants. [D] Cp-6711 toxin mutant supernatants at 10% and [E] purified PLC and PFO toxins at 800 ng/mL were added and monitored for YO-PRO uptake.

Given that pain is a common IBD symptom and other pore-forming toxins from *Staphylococcus aureus* and *Streptococcus pyogenes* activate sensory neurons to elicit pain during infection,^11,12^ we investigated whether the *C. perfringens* supernatants could activate neuronal cells. Upon activation, sensory neuron receptors such as TRPV1, TRPA1, and P2xR open pores that transport not only ions, but also larger cationic dyes such as the green fluorescent dye YO-PRO non-selectively.^56,57^ Taking advantage of this property, YO-PRO uptake and intracellular fluorescence can be used as a proxy for sensory neuron activation. Treatment of Neuro-2a mouse neuroblasts with *C. perfringens* supernatants encoding *pfoA* resulted in a greater fraction of fluorescent cells compared to treatment with Cp-6770 and media controls, consistent with increased neuronal activation [Figure 7A–C]. Activity began quickly, with many cells rounding and taking up YO-PRO before imaging began [Figure 7B]. Cp-6711 mutants of *pfoA* and *plc* both showed reduced activity compared to the parent strain [Figure 7D]. Addition of purified toxins was sufficient to induce YO-PRO uptake. Treatment with PFO and PFO2 resulted in similar fast kinetics of YO-PRO uptake, while PLC treatment required longer incubation before YO-PRO uptake was detected [Figure 7E], reinforcing the previous observations of fast and potent activity by PFO and PFO2.

To supplement our findings with Neuro-2a cells, we also tested primary mouse DRG neuron responses to *C. perfringens* factors. Although image analysis was complicated due to mixed cell populations common to DRG purifications, bulk image analysis revealed similar behaviours of *C. perfringens* supernatants inducing YO-PRO uptake as seen with the Neuro-2a cells [Supplementary Figure 17]. Treatment of DRG neurons with *C. perfringens* supernatants encoding *pfoA* resulted in more YO-PRO uptake compared to DRG neurons treated with Cp-6770 and media controls, consistent with increased neuronal activation [Supplementary Figure 17B]. Highly toxic strains, particularly Cp-6771, had increased variability hindering the accuracy of YO-PRO uptake measurements. Cells internalized the dye quickly and lysed. In contrast to the clear uptake of the YO-PRO dye observed with *C. perfringens* supernatants by the DRG neurons, both PLC and PFO purified toxins elicited minimal dye uptake [Supplementary Figure 17D].

We additionally tested DRG neuron activation by measuring calcium signalling, a direct recording of neuronal activity, with the calcium-sensitive dye Fura-2. Addition of neuron media or bacterial growth media alone did not lead to neuronal activation. However, incubation with Cp-6771 supernatant resulted in activation of a subpopulation of neurons consistent with direct bacterial modulation of neuronal signalling [Supplementary Figure 18]. Together, these results demonstrate the secreted factors from *C. perfringens* can activate sensory neurons.

## 4. Discussion

*Clostridium perfringens* is one of the most common causes of foodborne diarrhoea, causing an estimated 1 million foodborne illnesses every year in the USA.^58^ The five unique strains we isolated from five patients were all toxinotype A, haemolytic, and cytotoxic to a variety of cell types, including endothelial cells, human neutrophils, neurons, and PBMCs [Figures 5 and 6], but less so to intestinal epithelial cells. This suggests that the species acts as an opportunistic pathogen.^59^ Its ability to associate with the mucosa, rapid doubling rate [8–12 min] at feverish temperatures [43°C],^60^ and capacity to release multiple toxins^60^ may be significant in IBD. As a sporulating species, *C. perfringens* can reside in the GI tract without symptoms, germinate under favourable conditions, and return to the spore phase when conditions are unfavourable. This pattern aligns with typical disease patterns of IBD, characterized by phases of low activity and intermittent exacerbation.^61^ One speculation is that since both PLC and PFO toxins are regulated by quorum sensing,^62–64^ when conditions are favourable, colonized *C. perfringens* can replicate quickly to achieve a quorum, releasing multiple toxins capable of injuring various cell types.^65^ Without a quorum, toxins are not produced. When the intestinal epithelial barrier is intact, toxins produced by *C. perfringens* do not affect sensitive cell types residing beneath the intestinal epithelial layer [Supplementary Figure 19]. However, in cases when the epithelial barrier is damaged, as is the case for patients with IBD,^66,67^ the previously protected cell types become exposed to the toxins. This may lead to further destruction of the epithelial tissue, intestinal bleeding, an enhanced pro-inflammatory immune response, and intestinal pain. These factors collectively contribute to disease progression and worse outcomes in IBD patients.

Although the isolated strains do not encode the enterotoxin [*cpe*] commonly associated with diagnosed *C. perfringens* gastrointestinal illness,^13^*cpe*-negative toxinotype A strains have also been linked to cases of foodborne illness outbreaks.^49^ In a recent study, it was found that the presence of PFO and PFO2 toxins were strongly linked to virulent lineages of *C. perfringens* isolated from preterm infants with necrotizing enterocolitis.^15^ Those PFO-positive *C. perfringens* isolates, many of which also contained the beta2 toxin, caused more cellular damage both *in vitro* and in an *in vivo* mouse model compared to strains lacking PFO.^15^ In a separate study that examined 11 *C. perfringens* isolates obtained from humans, the haemolytic activity, PBMC lysis, and secretion of pro-inflammatory cytokines [IL-6 and IL-8] was found to correlate with PFO levels rather than PLC.^50^ Moreover, extensive studies of PLC and PFO have characterized their mechanisms in human and animal infections.^13^ PLC is a phospholipase shown to target phosphatidylcholine and sphingomyelin, while PFO, functioning as a cholesterol-dependent cytolysin, specifically targets cholesterol, facilitating membrane binding and pore formation.^13^ Additionally, PFO2 is hypothesized to have arisen from a gene duplication event of *pfoA*.^45^ Our data support PFO and PFO2 as the primary drivers of haemolytic activity *in vitro*, though synergy between PFO and PLC is likely *in vivo*.^68^ Collectively, these results suggest that toxinotype A strains may exert cytotoxic effects, particularly when the epithelial mucosal barrier is compromised, thereby accelerating the progression of IBD and leading to treatment failure. Therefore, these strains may play a significant role as detrimental agents in the gut.

Our findings with neuroblasts and primary mouse DRG neurons are particularly relevant for understanding the mechanisms underlying IBD-associated pain. Products from pathogenic bacteria, including toxins, have been shown to cause pain in acute pain and mouse abscess models through direct activation of sensory neurons.^11,69,70^ While IBD patient microbiomes are not considered to be pathogenic in the traditional definition, these experiments shed light on specific strains found in the microbiota that can produce toxins that directly activate neuroblasts and primary mouse DRG sensory neurons. This observation suggests, in addition to the conventional understanding that IBD-related pain results primarily from host inflammation, there exists an additional mechanism contributing to pain in IBD—one involving production of intestinal bacterial toxins.

While *C. perfringens* can cause gastrointestinal illness, its presence alone in the GI tract is not sufficient to cause IBD pathology, as it is found in asymptomatic individuals.^71^ Detecting its prevalence is challenging due to varied detection methods used, including direct culturing, sequencing [16S V4 or individual toxin DNA], and ELISA for toxin levels. Spore formation adds complexity, as spores are harder to detect than vegetative cells.^72,73^ With this consideration in mind, large-scale microbiome studies in healthy adult populations using whole metagenome sequencing have detected *C. perfringens* with prevalence ranging from 0.8% in the American human microbiome project^71^ to ~5% in the French *Milieu Intérieur* Consortium,^32,46^ Israeli consortium,^33^ and across 77 public WMS stool datasets. In contrast, *C. perfringens* prevalence in stool samples was ~4-fold higher [18–27%] in adult subjects with moderate to severe IBD compared to healthy subjects [Figure 3G]. Analysing two large IBD datasets processed independently by different vendors, along with multiple independent and sizable datasets from healthy individuals, gives us confidence these findings are not due to technical artefacts such as batch effects. Though stool samples may only reflect transient presence, such as from food intake rather than colonization, it is noteworthy that the large difference in prevalence remains consistent across multiple cohorts from diverse geographical regions and in the mucosa biopsies suggesting an IBD gut environment is more favourable to *C. perfringens* than a healthy one. Collectively, these findings reveal *C. perfringens* presence in healthy, asymptomatic individuals, but more commonly in IBD patients.

In this study, we consistently detected *C. perfringens* in the biopsy samples of patients when it was also detected in the stool, which implies mucosal interaction or colonization. In two patients, *C. perfringens* was exclusively detected in the biopsies and not stool. This observation may extend to adult patients as well. It raises the question of whether assessments of the stool microbiota in adults underestimate gastrointestinal presence of *C. perfringens*, as seen in our limited paediatric cohort.

Similarly to *C. perfringens*, *Clostridioides difficile* can be found in asymptomatic adults.^74^ However, *Clostridioides difficile* is routinely monitored in clinical settings due to its well-known potential to cause colitis. Among bacterial pathogens, *Clostridioides difficile* is the most established in its association with worse outcomes in IBD.^75,76^ Our observations here suggest *C. perfringens* infections may act similarly to *Clostridioides difficile* by exacerbating IBD symptoms. Interestingly, a case study involving two UC patients who underwent faecal microbiota transplant [FMT] for the management of *Clostridioides difficile* infection [CDI] reported an adverse event where severe diarrhoea developed^77^ were confirmed to be positive for enterotoxigenic *C. perfringens*. The authors of the study concluded *C. perfringens* is an overlooked pathogen and advocated for routine testing in FMT donor screening guidelines. Our findings support this call for increased attention to *C. perfringens* in clinical practice.

Our study has several limitations that should be acknowledged. We were unable to directly measure *C. perfringens* toxins within the tissue samples, resulting in an inability to demonstrate Koch’s postulates. Consequently, the physiologically relevant concentrations of *C. perfringens* and its products found in the local mucosal environments of these patients remain unknown. In future studies, it will be important to directly quantify both *C. perfringens* and toxin levels within the tissue itself. Despite evidence suggesting a potential role for *C. perfringens* in the pathogenesis of IBD, any causal relationship between *C. perfringens* and intestinal damage leading to IBD remains unclear. Additionally, it remains uncertain whether an IBD environment may simply be more hospitable to *C. perfringens* proliferation. Lastly, it is worth considering the possibility that we overlooked other bacterial pathogens, some of which may not express haemolysins yet still play a pivotal role in the pathogenesis of IBD.

Our study represents a significant advancement in the understanding of IBD pathology. It brings to light the identification of *C. perfringens* isolates derived from mucosal biopsies that possess the capacity to cause tissue damage and inflammation, two key features of IBD pathology. The presence of *C. perfringens* in this specific context is noteworthy, since this species has many unique attributes, including the ability to persist in a dormant spore state within the GI tract, rapid replication, production of numerous toxins, and well-established connections to causing infectious colitis.

Our *C. perfringens* findings may open up new avenues of therapeutic intervention for IBD. By recognizing that bacterial toxins can directly contribute to tissue damage, sensory neuron activation, and pain in IBD, researchers and clinicians may explore strategies to manage and ameliorate bacterially related IBD disease activity, not just for CDI. This may complement the conventional approach of solely addressing inflammation.

These results present a compelling rationale for the IBD medical community to consider actively monitoring *C. perfringens* as a potential contributor to disease activity. Furthermore, it prompts a critical examination of whether targeted antibiotic therapy to eliminate *C. perfringens* could yield therapeutic benefits for IBD patients housing *C. perfringens* in their GI tract. By expanding therapeutic approaches beyond traditional anti-inflammatories and addressing both bacterial and inflammatory aspects of disease, a synergistic effect of these interventions may significantly enhance management and overall outcomes for individuals with IBD.

## Supplementary Data

Supplementary data are available online at *ECCO-JCC* online.

jjae016_suppl_Supplementary_Tables

jjae016_suppl_Supplementary_Material

jjae016_suppl_Supplementary_Video_S1

jjae016_suppl_Supplementary_Video_S2
