# Supplementary material for: Toxigenic Clostridium perfringens Isolated from At-Risk Paediatric Inflammatory Bowel Disease Patients
Source: J Crohns Colitis. 2024 Jan 24;18(7):985–1001. doi: 10.1093/ecco-jcc/jjae016 (PMC11302968; doi:10.1093/ecco-jcc/jjae016)
Supplement: jjae016_suppl_Supplementary_Material [file jjae016_suppl_supplementary_material.pdf]

Supplementary Information

Toxigenic *Clostridium perfringens* isolated from at-risk paediatric inflammatory bowel disease patients

James Kuo[1], Jasmina Uzunovic[2], Amanda Jacobson[3], Michelle Dourado[4], Sarah Gierke[5], Manohary Rajendram[1], Daniela Keilberg[1], Jordan Mar[6], Emily Stekol[7], Joanna Curry[7], Sofia Verstraete[7], Jessica Lund[8], Yuxin Liang[8], Fiona B. Tamburini[6], Natalie S. Omattage[1], Matthieu Masureel[9], Steven T. Rutherford[1], David H. Hackos[4], Man-Wah Tan[1], Allyson L. Byrd[10], Mary E. Keir[6], Elizabeth Skippington[1,2], Kelly M. Storek[1,*]

[1]Department of Infectious Diseases and Host-Microbe Interactions, Genentech Inc., South San Francisco, CA, USA

[2]Department of Bioinformatics, Genentech Inc., South San Francisco, CA, USA

[3]Department of Immunology Discovery, Genentech Inc., South San Francisco, CA, USA

[4]Department of Neuroscience, Genentech Inc., South San Francisco, CA, USA

[5]Department of Pathology, Genentech Inc., South San Francisco, CA, USA

[6]Department of Human Pathobiology and OMNI Reverse Translation, Genentech Inc., South San Francisco, CA, USA

[7]Department of Pediatrics, University of California San Francisco Benioff Children's Hospital, San Francisco, CA, 94158, USA.

[8]Department of Microchemistry, Proteomics & Lipidomics, Genentech Inc., South San Francisco, CA, USA

[9]Department of Structural Biology, Genentech Inc., South San Francisco, CA, USA

[10]Department of Cancer Immunology, Genentech Inc., South San Francisco, CA, USA

***Correspondence:** storek.kelly@gene.com. 408-309-7014

SUPPLEMENTARY MATERIALS and METHODS

Study Design and Patients

A prospective case-control study of patients aged 2 years and older undergoing esophagogastroduodenoscopy (EGD) and colonoscopy as part of clinical care at UCSF Benioff Children's Hospital Mission Bay was conducted as approved by the UCSF Institutional Review Board. Patients who had taken antibiotics, proton pump inhibitors (PPI), or H2-receptor antagonists within two weeks of procedure, with current parasites (*Cryptosporidium*, *Entamoeba histolytica*, *Giardia*), bacteria (*C. difficile*, *Campylobacter*, *E. coli* O157, Enterotoxigenic *E. coli* (ETEC) LT/ST, *Salmonella*, *Shigella*, shiga toxin-producing *E. coli* (STEC) stx1/stx2, *Vibrio cholera*), and viruses (adenovirus 40/41, Norovirus GI/GII, Rotavirus A) in the stool culture as detected by PCR or stool culture + Ova and Parasites (O&P) stool test within two months of procedure, history of transplant (solid organ or HSCT), history of gastric or intestinal bypass surgery, current motility disorder, advanced comorbid conditions, inability to stop high-risk medications such as antiplatelets or anticoagulants, or known immunodeficiencies, malignancies, or human immunodeficiency virus were excluded from this study.

Patients were contacted via phone or email one to two weeks before their scheduled procedure to have a complete consent discussion with the study team. Once consented, stool collection kits were mailed to the patient's home to facilitate stool collection ahead of their scheduled cleanout. Patients were instructed to collect stool in OMNIgene-GUT OMR-200 tubes (DNA Genotek) within 1-3 days of their procedure. Patients were prepared for a standard endoscopy with MiraLAX and Dulcolax. During the procedure, two mucosal biopsies were obtained according to standard clinical practice from the duodenum or jejunum, ileum, and colon and placed in BIOME-Preserve anaerobic microbiome tubes. When present, biopsies were obtained from diseased areas of the colon.

Demographic data (sex, race, ethnicity, date of birth), height and weight at time of procedure, nicotine exposure, disease information (UC, CD, other GI disorder, or none), and medication history (including antibiotic exposure) were collected. Abnormalities macroscopically and microscopically at the time of the endoscopy were noted.

Culturing and processing patient samples

Typically from each patient was collected (a) two biopsies each from three sections (duodenum, ileum, colon) stored in Anaerobic Tissue Transport Media (Anaerobe Systems AS-919), (b) pre-biopsy colonic phosphate buffer wash (stored in BIOME-Preserve Anaerobic

Microbiome Collection tubes (Anaerobe Systems)), and (c) stool collected before the procedure (also stored in BIOME-Preserve tubes or OMNIgene-GUT OMR-200 tubes (DNA Genotek). Samples were transported within 4 hours of the procedure and processed — culturing the biopsies and freezing the remaining samples. Biopsies were transferred to 1-2 mL of PBS in a gentleMACS M-tube (Miltenyi Biotec) using a 1 mL pipette tip in an anaerobic chamber (Coy, vinyl). M-tubes were sealed tightly, removed from the anaerobic chamber and homogenized using a gentleMACS Dissociator (Miltenyi Biotec) for 55 seconds (RNA_01.01 setting) 1-3 times. The tubes were then brought back into the anaerobic chamber and processed for culturing.

Culturing was with 1 mL media in 2 mL 96-deepwell polypropylene plates (Corning 3960). To each 1 mL media, ~50 µL of homogenized biopsy slurry was added. Plates were sealed with AeraSeal films (Excel Scientific BS-25) and incubated at 37°C anaerobically. After 2-3 days, the plates were passaged by transferring 10 µL to a fresh 1 mL of media, then grown an additional 2-3 days. After each round of growth, cultures were saved as glycerol plates (50 µL culture + 50 µL of 50% glycerol), neat plates (100 µL culture), and DNA extraction plates (250 µL culture). After the second growth passage, cultures were additionally processed as supernatant plates for in vitro assays, prepared by spinning down 300+ µL of culture for 3,000 x g for 5-10 min, then filtering supernatant with a 0.2 µm PES AcroPrep Advance 96-well filter plate (PALL 8019), spinning 1,500 x g for 2 min. Note that some supernatants clogged the filter. Supernatants were collected in polystyrene 96-well round bottom plates (Corning 3788). Plates were sealed with aluminum foil covers (Excel Scientific FCS-25) and stored at -80°C.

Culturing media was selected based on previous efforts[1], including Mega media[2], simulated intestinal efflux media (SIEM)[3], simulator of the human intestinal microbial ecosystem (SHIME) media[4], and a DMEM-based media with mucin and pectin for chip culture[5]. Notably, another recent effort culturing from human samples also chose BHI[6].

C. perfringens culturing and genetics

To increase our odds of isolating *C. perfringens,* we selected cultures with the highest *C. perfringens* relative abundances for colony plating on sulfite-containing plates and used conditions to select for spores, like aerobic ethanol treatment[7].

Insertional mutants were generated using the Group II intron system, or “ClosTron”, developed previously[8]. We created plasmids based on what was used recently[9] containing a thiolase-promoter driven erythromycin resistance (ermB) gene and a 309 bp variable intron targeting region. PCR was performed using Q5 Hot Start Master Mix (New England BioLabs,

M0494), Gibson assemblies using NEBuilder HiFi DNA Assembly mix (New England BioLabs, E2621), and plasmid cloning was in *E. coli* TOP10 chemically competent cells (Invitrogen). Plasmids were transformed by electroporation into *E. coli* CA434 for conjugation. Overnight cultures of the donor *E. coli* and recipient *C. perfringens* were mixed in a 2:1 ratio (1 mL + 0.5 mL) and spun down 3,000 x g for 2 min. The supernatant was removed to leave about 20-30 µL, which was then spotted on a tryptic soy broth (TSB) plate for 24-48 h conjugation at 37°C anaerobically. Spots were scraped into PBS and plated onto selection agar plates, BHI-YH + 15 µg/mL thiamphenicol + 250 µg/mL cycloserine, then restreaked onto BHI-YH + 10 µg/mL erythromycin + 250 µg/mL cycloserine agar plates. Mutants were checked by sequencing.

DNA Extraction and 16S_V4 sequencing, data processing, and analysis

Microbial DNA was extracted from ~200 µL of culture using DNeasy QIACubeHT Powersoil Pro (Qiagen) for Patients 1-3 or DNeasy 96 PowerSoil Pro (Qiagen) Patients 4-9. Biopsies were also directly extracted using the single tubes QIAamp PowerFecal Pro DNA kit (Qiagen). Extracted DNA was used for 16S_V4 PCR using barcoded reverse primers[10]. PCR was done with Takara Ex Taq DNA Polymerase, Hot Start version (RR006B) and Roche BSA (#10711454001) as previously described[11].

16S libraries were pooled and sequenced following Illumina's metagenomics workflow. The library pool was denatured with 0.2 N NaOH for 5 mins and diluted to 8 pM with the Illumina buffer HT1. The Illumina control library PhiX (denatured and diluted) was spiked into the diluted denatured library at 5%. The mixture was heat denatured at 96°C for 2 minutes, chilled in an ice-water bath for 5 minutes, then added to the MiSeq cartridge along with custom primers spiked into the corresponding Illumina primer wells and sequenced paired end 200 cycles with a 12 base i7 index. Microbiome sequence data is available from The European Genome-phenome Archive (EGA, https://ega-archive.org/) under Study ID EGAS00001007538.

QIIMEv2019.7 was used to process the 16S-V4 rRNA gene sequence data as previously described[12,13]. Briefly, raw sequence data was demultiplexed, read trimming was performed to remove regions of low sequence quality, and paired-end reads were denoised, dereplicated, and chimera filtered with DADA2[14]. Taxonomy was assigned based on the V4 region of the Genome Taxonomy Database 16S rRNA gene sequence database (r202)[15,16]. Resulting abundance tables were rarefied and data was visualized using MicrobiomeExplorer[17]. β-diversity measures were calculated in QIIMEv2019.7 [12,13]. All statistical analyses were conducted in the R programming language (v4.3.0) (R Core Team. R: A language and environment for statistical computing. Vienna: Austria; 2020.). Bray-Curtis distance matrices

were visualized via non-metric multidimensional scaling (NMDS) and permutational multivariate analysis of variance (PERMANOVA) was used to determine relationships between metadata (e.g., sample location) and bacterial microbiota composition using the adonis function from the the vegan package (Oksanen J, Simpson G, Blanchet F, Kindt R, Legendre P, Minchin P, et al. vegan: Community Ecology Package. R package version 2.6-4. 2022.). Plots were generated in R v4.3.0 using packages from the tidyverse v2.0.0 suite (dplyr v1.1.3, ggplot2 v3.4.3)[18].

Oxford Nanopore whole-genome sequencing and analysis

Genomic DNA was sequenced either individually on MinION flow cells or multiplexed on PromethION flow cells. DNA quality was determined by Nanodrop (Thermo Fisher Scientific) to ensure an OD_{260}/OD_{280} of 1.8-2.0 and an OD_{260}/OD_{230} of 2.0–2.2 for all library input. DNA was quantified using the Qubit dsDNA BR assay kit (Thermo Fisher Scientific). DNA size was determined using the Genomic DNA ScreenTape on TapeStation 4200 (Agilent Technologies). For library preparation, the Ligation Sequencing Kit (Oxford Nanopore Technologies, SQK-LSK109) was used with an input of 1 µg of genomic DNA. Sample multiplexing was performed using 500 ng of end-repaired DNA with the Native Barcoding Expansion Kit (Oxford Nanopore Technologies, EXP-NBD104). 400 ng of library from each sample was sequenced on an R9.4 flow cell on a MinION device to generate fast5 files that were subsequently converted to fastq files using Guppy 5.0.11 (Oxford Nanopore Technologies). An average of 350 ng of multiplexed library pool of 3 or 4 samples was sequenced on an R9.4 flow cell on a PromethION device and base-called in real time using Guppy 5.1.12.

Draft metagenomes were assembled using metaFlye 2.8.3-b1695 with the '–meta–plasmids' option[19]. To identify virulence genes, the assembled metagenomes were compared against the VFDB[20], using ABRicate v 0.5 (https://github.com/tseemann/abricate) with default parameter settings. BLAST high-scoring segment pairs (HSPs) satisfying >80% sequence identity were considered putative evidence for presence of a virulence factor protein gene. Contigs putatively encoding virulence factors were assigned species labels via BLASTn searches against the NCBI non-redundant database. Long-read data is available from NCBI under BioProject ID PRJNA1043401 with BioSample accessions SAMN38369910-18.

Whole-genome sequencing and analysis (Illumina)

Quality of the genomic DNA was determined using the Genomic DNA ScreenTape and TapeStation 4200 (Agilent Technologies). Genomic DNA was quantified using the Qubit dsDNA BR assay kit (Thermo Fisher Scientific). For library preparation, the Nextera DNA Flex kit

(Illumina) was used with an input of 100 ng of genomic DNA. The resulting libraries were multiplexed and sequenced on NovaSeq (Illumina) to generate 5 million paired end 75 base pair reads for each sample.

Draft isolate genomes were assembled from Illumina short read data using SPAdes v3.13.1 with default parameter settings[21], and BRIG was used to visually compare them[22]. Gene calling was performed using Prokka v1.12 [23] and genome quality assessed using CheckM v1.2.2 [24]. Roary with default parameter settings[25] was used to produce a core gene alignment of *C. perfringens* genomes sequenced in the current study, as well as publicly available genomes. The *C. perfringens* phylogeny was estimated with RAxML v8 using the generalized time-reversible (GTR) model with gamma correction for among-site rate variation and 10 starting trees[26]. Support for nodes was assessed using 500 bootstrap replicates. All *C. perfringens* genome data is available from NCBI under BioProject ID PRJNA1043401 with BioSample accessions SAMN38338936-40.

Red blood cell lysis assays

Whole blood from healthy subjects was obtained with Genentech's internal donor program, Samples for Science, following the approved IRB protocol (20080040) study specifications. Bacterial supernatants to 10% were mixed with 2% whole blood in phosphate-buffered saline to a final volume of 200 µL in a 96-well plate. Plates were incubated at 37℃ for the specified amount of time, and for 4 h for single point readings. At each time point, 50 µL of supernatant was removed and mixed with 50 µL of PBS. The 100% lysis control was 0.5% Triton X-100. Absorbance at 450 nm was subtracted from the 650 nm absorbance.

Toxin Purification, Protein Gels, Western Blots

Toxins were expressed with 6xHis and SUMO cleavage tags in *E. coli* grown in Terrific Broth. Cells were resuspended in lysis buffer (50 mM Tris pH 7.5, 300 mM NaCl, 15% glycerol, 5 mM imidazole, 5 mM $MgCl_2$, 1 mM TCEP (Tris(2-carboxyethyl)phosphine)) with Roche cOmplete Ultra EDTA-free protease inhibitor (5892970001) and sonicated 3x 1 min (1 s on, 1 s off). The lysates were incubated with AmMag Ni resin magnetic beads (Genscript L00776). Resin was incubated with 50 mM Tris pH 7.5, 300 mM NaCl, 15% glycerol, 1 mM TCEP with 20 mM imidazole for washes and 250 mM imidazole for elution. The eluate was concentrated using a 10 kD Amicon Ultra centrifugal filter. Proteins were further purified by size exclusion chromatography at 4°C with a Superdex 200 Increase 10/300 GL column (GE Healthcare) and 20 mM HEPES pH 8.0 with 150 mM NaCl on an AKTA pure (Cytiva).

Protein SDS-PAGE was run with 10% Bis-Tris gels (Invitrogen, 1.0 mm for Western blots, or 1.5 mm otherwise) with MOPS running buffer. SimplyBlue SafeStain (Invitrogen) was used for visualization according to manufacturer instructions. Western blots were run using Invitrogen's iBlot 2 system: nitrocellulose membrane with primary anti-PFO polyclonal antibody (Invitrogen PA5-117550, 3 mg/mL, rabbit IgG) at 1:2500 and secondary anti-rabbit (Li-Cor IRDye 800CW, 1 mg/mL) at 1:5000. Commercial recombinant PFO protein (ATCC BTX-100) was used for comparison. For alpha-toxin, primary anti-alpha-toxin polyclonal antibody (3A4D10-23.0, Absolute Antibody, 1 mg/mL, rabbit IgG) was used at 1:2500.

PCR and sequencing analysis of biopsy RNA

Biopsy slurries in PBS were extracted using the AllPrep PowerViral DNA/RNA kit (Qiagen) with added on-column DNase I (Qiagen) step before final wash and elution. RNA was further treated with DNase using the TURBO DNA-free kit (Invitrogen AM1907) to ensure genomic DNA removal prior to cDNA prep by iScript Reverse Transcription (Bio-Rad). Both final cDNA and pre-reverse transcription RNA were used as template to run PCR analysis with Q5 Hot Start mix: 8 µL reactions with 0.8 µL template and 500 nM each primer (**Supplementary Table 11**). Thermocycling: 98°C for 2 min, 40x cycles [98°C for 10 sec, 58°C for 30 sec, 72°C for 30 sec], 72°C for 10 min, then hold at 4°C. The first PCR reaction was followed by a second using 0.8 µL of the first PCR reaction as template. Reactions were visualized on 4% agarose E-gels (Invitrogen), and positive hits were Sanger sequenced to verify.

CellTiter-Glo cell viability assays to estimate toxicity

Mammalian cells were cultured using standard techniques in respective growth media in T75 or T150 flasks. Cell lines (T84, HT29, Caco2, Neuro-2a) were grown from in-house stocks originally passaged from ATCC. T84 cells were grown in RPMI 1640 with 10% heat-inactivated fetal bovine serum (FBS, R&D Systems), 2 mM Glutamax and Pen-Strep. HT29, Caco-2 and Neuro-2a cells were grown in DMEM with 10% FBS, 2 mM GlutaMAX and 1X Pen-Strep (Gibco). HUVEC cells (PromoCell) were grown in Endothelial Cell Growth Media (PromoCell). Cells were seeded at ~10,000 cells per well in white clearbottom tissue culture treated 96-well plates (Corning 3610) and grown an additional 1-3 days depending on the cell type. For human colonic organoid-derived monolayers, organoids were derived from deceased adult Donor Network West donors. Two donors were used in this study: a 36-year-old Asian Indian female and a 53-year-old White female. Organoids were grown in expansion media modified from previous protocols[27]. Organoids were dissociated to single cells and plated at a density of

100,000 per well on Collagen IV (Sigma C5533) coated plates. Organoid-derived monolayers were differentiated for 7 days. Media was swapped with low serum (0.25% FBS) or serum-free (for HUVEC) media, and incubated with test compounds at 4-10% overnight (16-24 h). Cell viability was then measured using CellTiter-Glo Luminescent Cell Viability Assay (Promega) according to kit instructions, with luminescence read out on a SpectraMax M5 plate reader at 500 ms integration time. Roche's Cytotoxicity Detection Kit (LDH) was used according to kit instructions.

Human neutrophils were extracted from whole blood using the EasySep Direct Human Neutrophil Isolation Kit (STEMCELL Technologies), resuspended in RPMI 1640 with 10% FBS, 2 mM Glutamax and Pen-Strep, and assayed a few hours after seeding. Human PBMCs were purchased from STEMCELL Technologies. PBMCs were incubated with compounds and the resulting supernatants submitted for Luminex cytokine analysis using the Bio-Rad 23-plus cytokine panel. Values below the detection range were set as zero. Phase contrast imaging for select cytotoxicity assays was done at 10X magnification on an Incucyte S3 (Sartorius).

For YO-PRO toxicity studies, cells were incubated at the same conditions as for CellTiter-Glo assays but with YO-PRO-1 dye (Invitrogen Y3603) at 1 µM, imaged every 20-30 min at 20X on an Incucyte S3 in phase contrast and green fluorescence channels (300 ms exposure, 441–481 nm excitation, 503–544 nm emission).

Neuron-activation assays and DRG isolation

Neuro-2a cells were incubated in DMEM with 0.25% FBS (as used in toxicity assays) with YO-PRO-1 dye and test compounds added, and imaged every 5 min on an Incucyte S3 in phase contrast and green fluorescence channels (300 ms exposure, 441–481 nm excitation, 503–544 nm emission). YO-PRO uptake was estimated as the area of positive green fluorescence divided by total area of cells segmented in the phase contrast channel using the Incucyte analysis software– note that this underrepresents the fraction of fluorescent cells due to differences in tracked phase area and fluorescence-positive area.

Mouse DRGs were isolated from mice and processed as previously described[28]. Briefly, wild-type mice were euthanized by CO_2 inhalation, and DRGs were quickly excised into cold Hanks' Balanced Salt Solution (HBSS, Gibco) and dural sheaths were removed. The DRGs were then placed in divalent-free HBSS and enzymatically digested using a combination of collagenase and trypsin with gentle shaking. After several spins and washes, DRGs were placed in DMEM containing 10% inactivated horse serum, and dissociated by gentle trituration using 1 mL and 200 mL pipet tips. The dissociated cell suspensions were purified by myelin

elimination using magnetic anti-myelin beads. The purified cells were counted and suspended in HibernateA containing 10% inactivated horse serum. The cells were plated on 96 well PDL-coated plates (BD) and incubated overnight at 37°C for YO-PRO influx measurements, and on Poly-D-Lysine-coated glass coverslips placed in a multiwell culture dish for Calcium influx measurements. Plated cells were then flooded with complete cell medium consisting of DMEM, 10% heat-inactivated horse serum, 2 mM L-glutamine, 0.8% D-glucose, 100 units penicillin, and 100 mg/ml streptomycin. YO-PRO uptake and calcium-influx measurements were performed within 24 hours of cell isolation.

Mouse DRGs were incubated in DMEM with 10% FBS and preincubated with YO-PRO-1 dye for 20+ minutes before *C. perfringens* supernatant addition. Cells were then imaged every 5 minutes for 4 h in phase contrast and FITC channels with a Plan Apo 20x (NA 0.75, Nikon) objective on a Nikon TI-E perfect focus inverted microscope equipped with a Neo sCMOS camera (Andor, Oxford Instruments), 37°C/5% CO_2 environmental chamber (Okolab), and a SOLA V-NIR LED lightsource (Lumencor), all run by NIS Elements software (Nikon). For purified toxin experiments, cells were imaged every 3 min for 2.5 h in phase contrast and 488 nm laser channels with a similar Nikon microscope setup equipped with a spinning disk confocal CSU-X1 (Andor, Oxford Instruments), ILE laser launch (Spectral Applied Research), and Prime 95B sCMOS camera (Teledyne Photometrics). YO-PRO uptake was analyzed by the sum green fluorescence intensity above a set threshold, normalized to the initial area of cells segmented in phase-contrast channel in NIS Elements software.

Prevalence estimate of C. perfringens

Preprocessing and taxonomy classification were performed on metagenomic sequence reads as previously described[29]. Briefly, reads were de-replicated using PrinSeq v0.20.4, trimmed using trimmomatic v0.39, and aligned to PhiX and the Pacific Biosciences human genome (GenBank GCA_000772585.1) using bowtie2 v2.4.1. Unmapped reads were subsequently classified with kraken v2.1.1 with a confidence parameter of 0.2 (“--confidence 0.2”) against a custom reference database containing 112,320 genomes from 64,468 species (61,220 bacterial, 3,248 archaeal) from the Genome Taxonomy Database (GTDB) release 207 [15,16] and then processed with bracken v2.5 with a read threshold of 250 (“-t 250”). Prevalence of *C. perfringens* was calculated from bracken output for two independent healthy cohorts (Milieu Intérieur, Israeli), three UC cohorts, and two CD cohorts. The Milieu Intérieur cohort included 500 men and 500 women between 20 and 69 years of age from the suburban Rennes area (Ille-et-Vilaine, Bretagne, France)[29]. Stool aliquots were processed, DNA extracted, and shotgun

metagenomic sequencing was performed by Diversigen, Inc. as described previously[29]. The Israeli cohort included 1,159 samples from 851 non-diabetic donors between 20 and 69 years of age[30]. The CD (BERGAMOT cohorts 1, 2, 3) and UC (LAUREL, HIBISCUS I and II) cohorts were obtained from five phase 3 randomized clinical trials comparing the safety and efficacy of etrolizumab in patients from 18 to 80 years of age with moderate-to-severe active UC or CD[31–33]. Stool samples from BERGAMOT cohort 1 and LAUREL were processed, DNA extracted, and shotgun metagenomic sequenced by Microbiotica, Ltd. Stool samples from BERGAMOT 2 and 3 and HIBISCUS I and II were processed, DNA extracted, and shotgun metagenomic sequenced by CosmosID.

DNA was extracted from biopsy tissue from LAUREL, HICKORY, and BERGAMOT cohort 1 using the AllPrep DNA/RNA Kit (Qiagen) with bead beating. 16S sequencing of the V3_V4 region was performed by Microbiotica, Ltd. using the primers 357F and 800R. 16S sequences were quality trimmed, denoised, dereplicated, and filtered for chimeras using QIIME2 v2019.7. Taxonomy was assigned using a custom database of 16S rRNA sequences from GTDB release 207 and prevalence of *C. perfringens* was calculated from species-level count tables.

Using the curatedMetagenomicData R package (v3.9.1)[34], *C. perfringens* relative abundances, as determined by MetaPhlAn3, and sample metadata were retrieved for 21,030 stool samples with the command “returnSamples”. Of those, the 13,827 stool samples with disease equal to “healthy” or study_condition equal to “control” were selected, along with 2,054 samples with disease equal to “IBD”, from 77 studies (**Supplementary Table 6**). Some subjects were sampled more than once: relative abundance values for *C. perfringens* were averaged per-subject where indicated.

SUPPLEMENTARY FIGURES

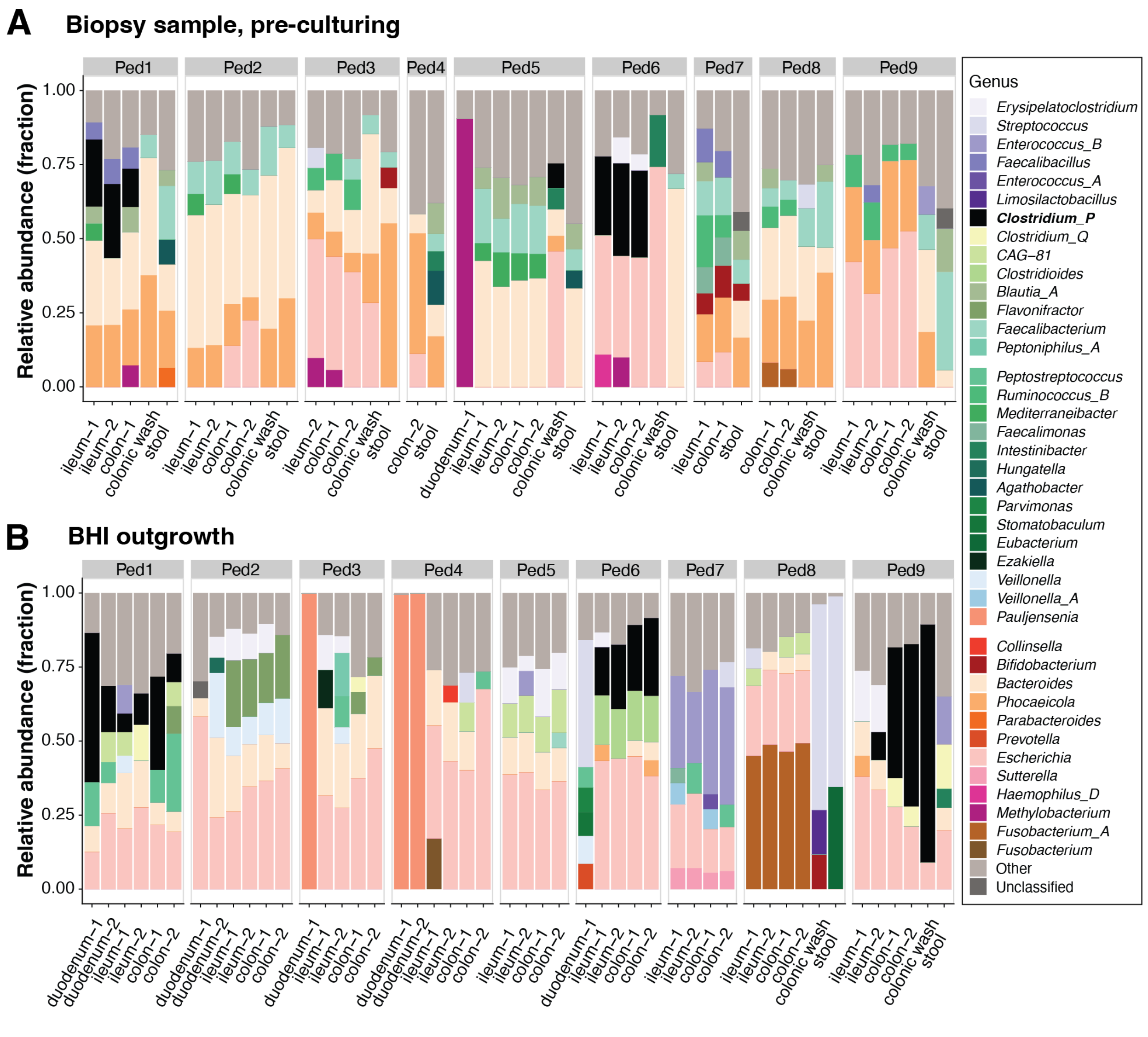

Supplementary Figure 1. Variation in microbiota composition of colonic biopsy outgrowth across different growth media. Patient samples sequenced (A) directly as biopsies pre-culturing and (B) after the second passage outgrowth in BHI media. Samples were sequenced using 16S V4 sequencing and classified at the genus taxonomic rank. *Clostridium_P* (black, bold) is *Clostridium perfringens*. Minimum 10,000 reads filtering cutoff per sample.

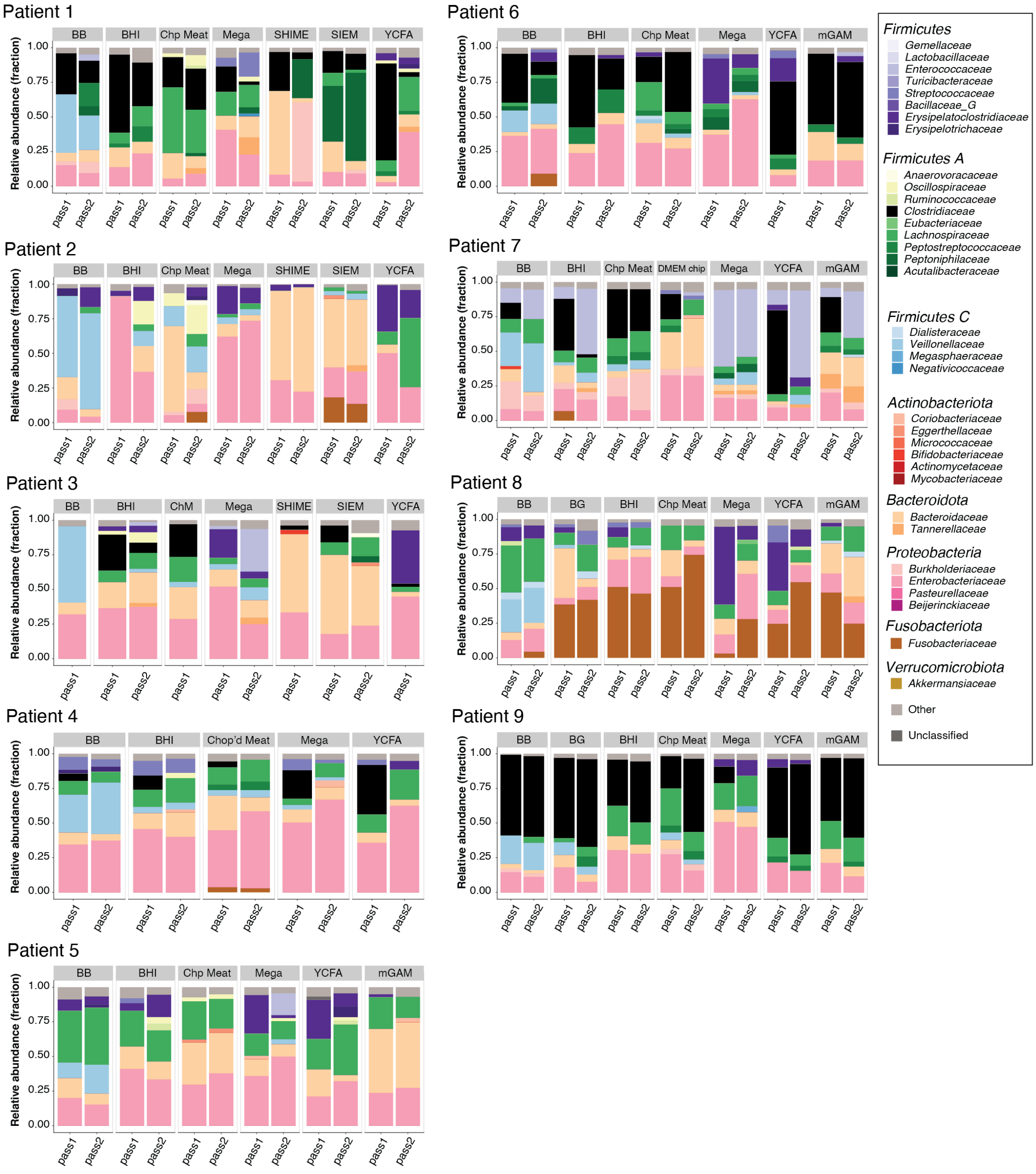

Supplementary Figure 2. Variation in microbiota composition of colonic biopsy outgrowth across different growth media. The microbiota outgrowth of one colonic biopsy from each patient grown in different media was sequenced using 16S V4 rRNA and classified at family taxonomic rank. Filtering cutoff per sample: minimum 10,000 reads. Both passages shown. BB, Bryant and Burkey; BHI, brain heart infusion; Chp Meat/ChM/Chp'd Meat, chopped meat; SHIME, simulator of the human intestinal microbial ecosystem; SIEM, simulated intestinal efflux media; YCFA, yeast casitone fatty acids (modified); mGAM, modified Gifu Anaerobic Medium; BG, BB + mGAM combo.

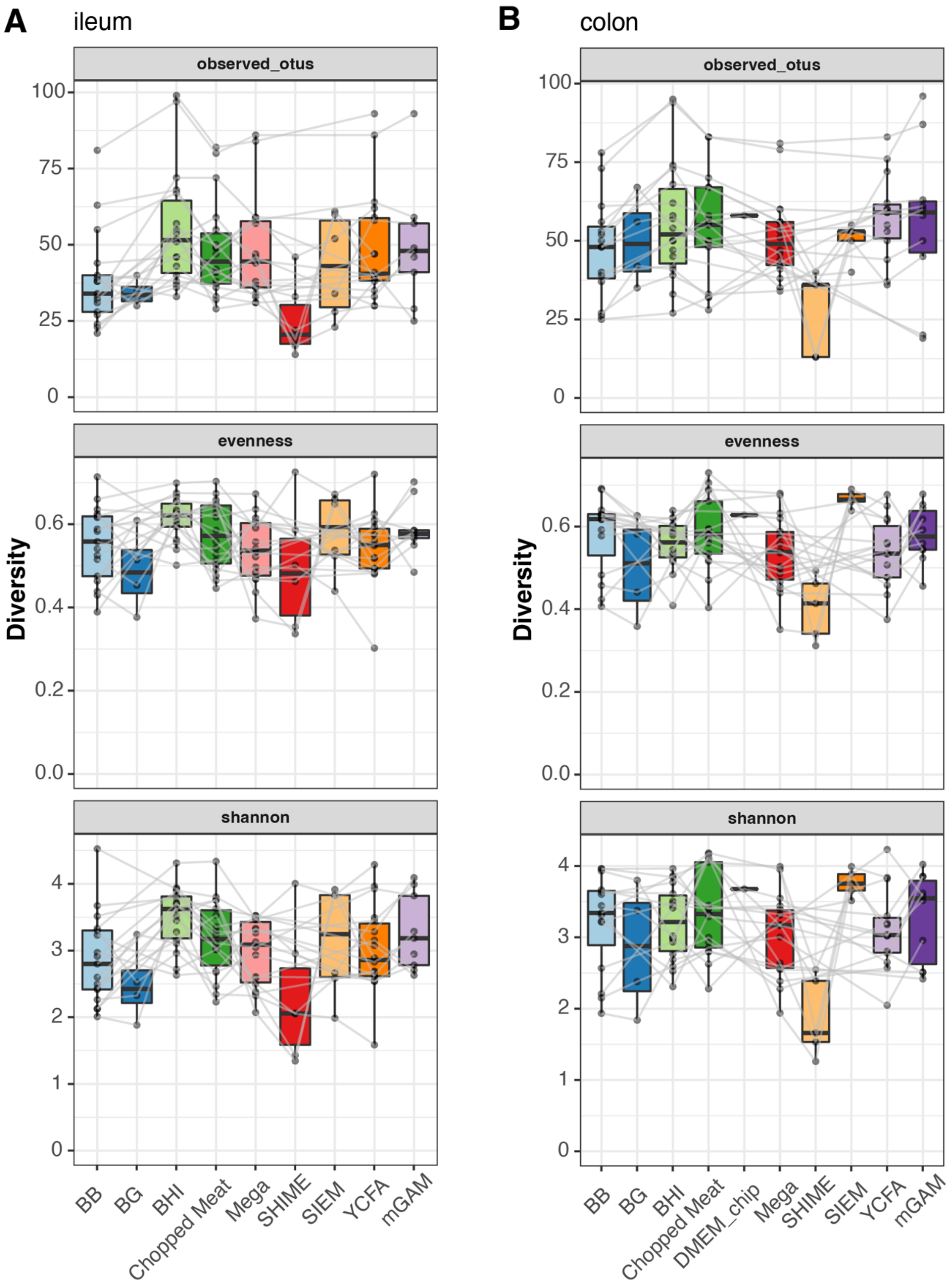

Supplementary Figure 3. α-diversity of microbial communities grown from ileal and colonic biopsies in various media. 16S V4 sequence data from (A) ileal and (B) colonic biopsy outgrowth in different growth mediums from the second passage for each patient. Top panel: Total number of observed OTUs (richness). Middle panel: Community evenness. Bottom panel: Shannon diversity index.

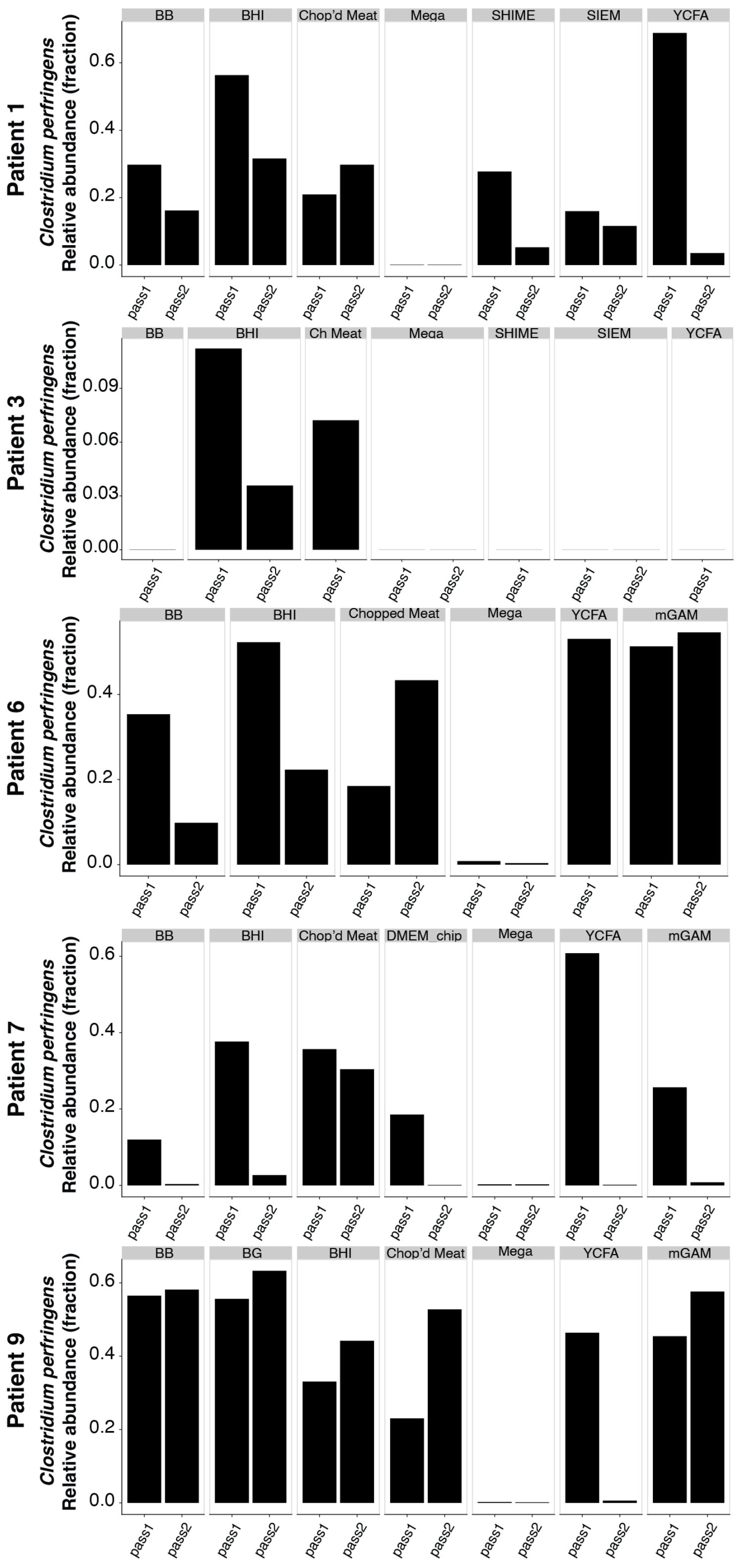

Supplementary Figure 4. Impact of growth media and passage number on the outgrowth of *C. perfringens* from patient biopsies. 16S V4 sequencing is sufficient to classify *C. perfringens* to the species level. *C. perfringens* abundance from the colonic biopsy outgrowth in different growth conditions is shown for each patient where *C. perfringens* was detected after outgrowth. Both passages are shown.

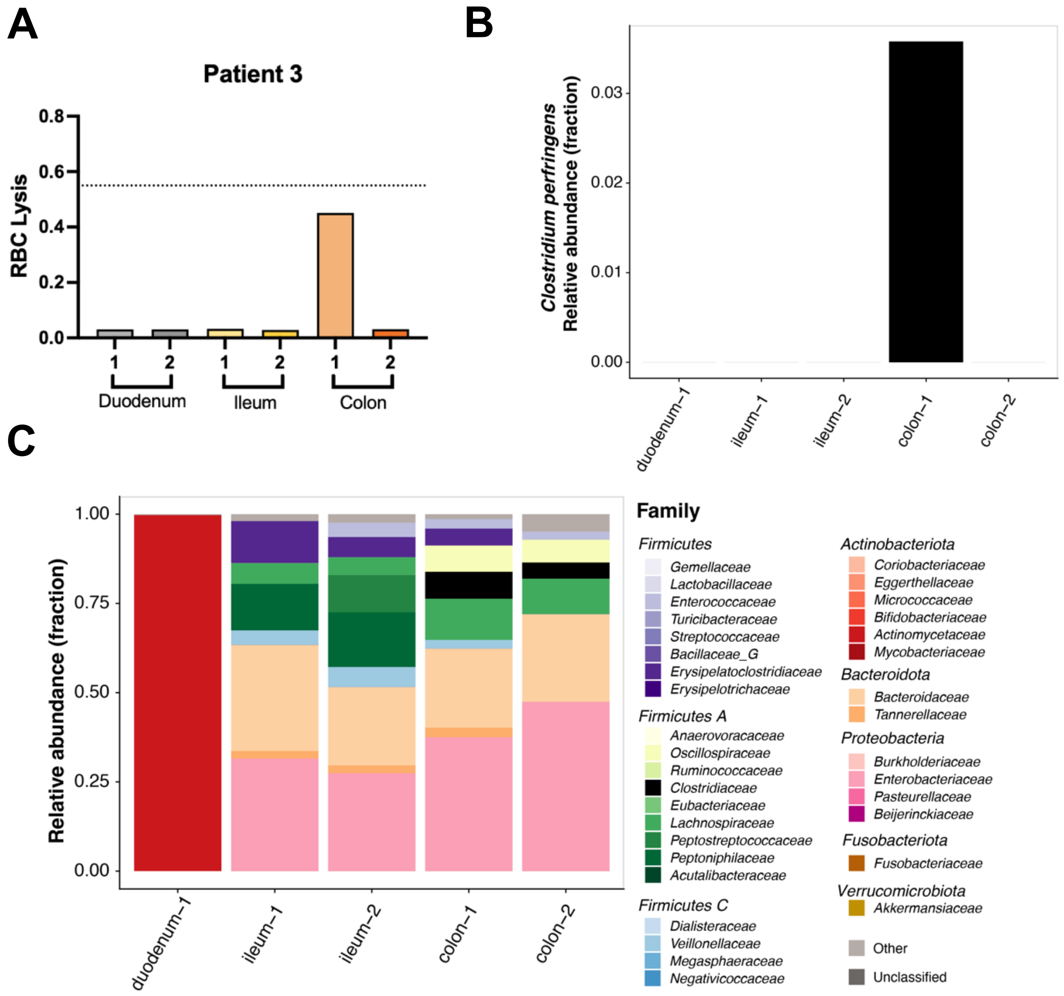

Supplementary Figure 5. Correlation between hemolytic activity and *C. perfringens* presence in a colonic biopsy from Patient 3. (A) RBC lysis measured after 4 hour incubation with 10% bacterial supernatants from the BHI outgrowth after two passages of Patient 3 biopsies. RBC lysis was measured as (OD_{450}-OD_{650}). Dotted lines represent lysis with 0.5% Triton X-100 as the 100% lysis control. 16S V4 sequencing from the matched BHI outgrown communities were performed: (B) *C. perfringens* community abundance and (C) family taxonomic rank are shown (note that *Clostridiaceae* also includes other species besides *C. perfringens*).

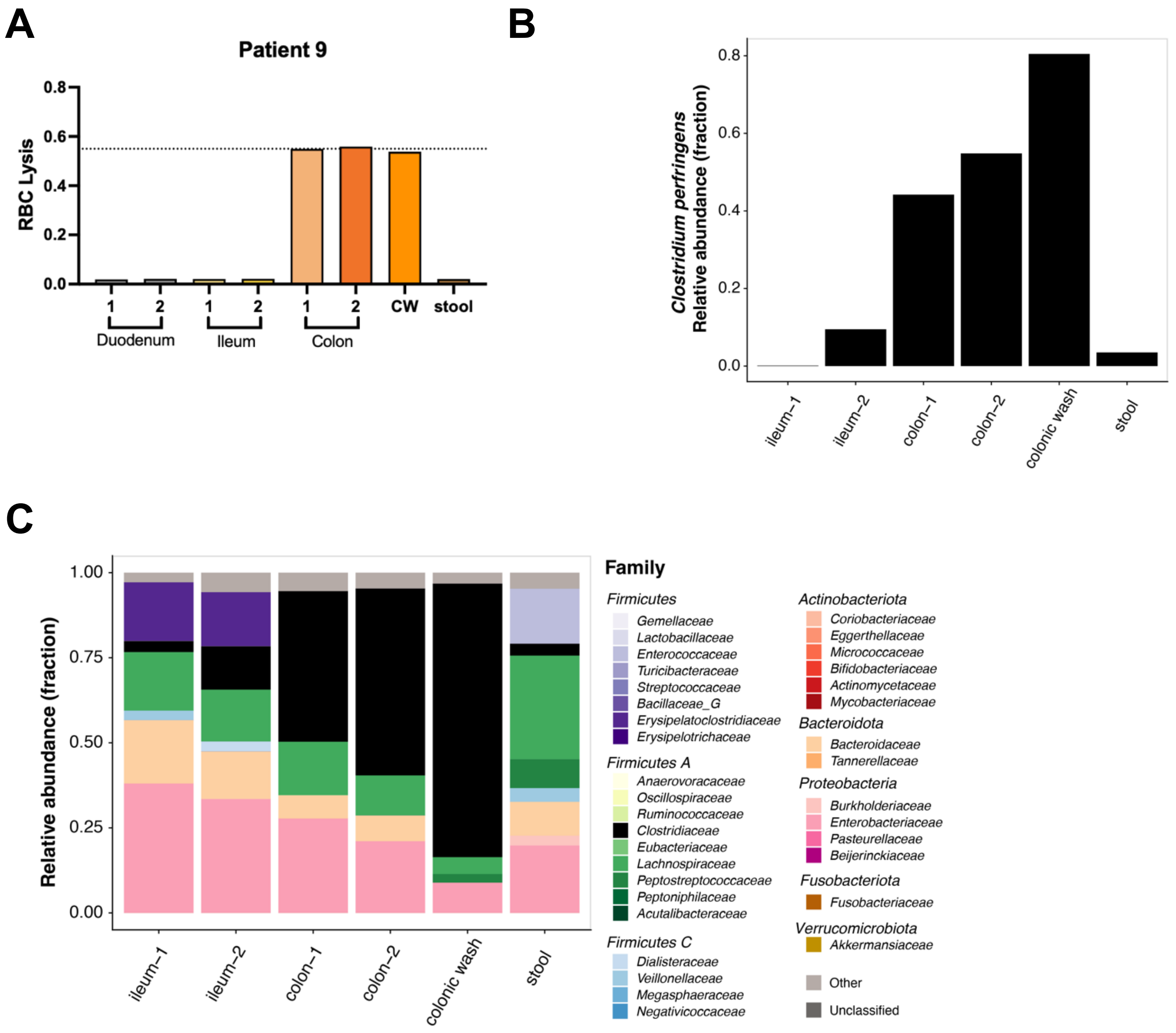

Supplementary Figure 6. Elevated hemolytic activity in biopsy outgrowth compared to stool from Patient 9. (A) RBC lysis measured after 4 hour incubation with 10% bacterial supernatants from the BHI outgrowth supernatants after two passages of Patient 9 biopsies, colonic wash (CW), and stool samples. Dotted lines indicate lysis with 0.5% Triton X-100 as the 100% lysis control. Two biopsies from each location were evaluated for activity. RBC lysis was measured as (OD_{450}-OD_{650}). 16S V4 sequence from the matched BHI community was performed: (B) *C. perfringens* community abundance and (C) family taxonomic rank are shown (note that *Clostridiaceae* also includes other species besides *C. perfringens*).

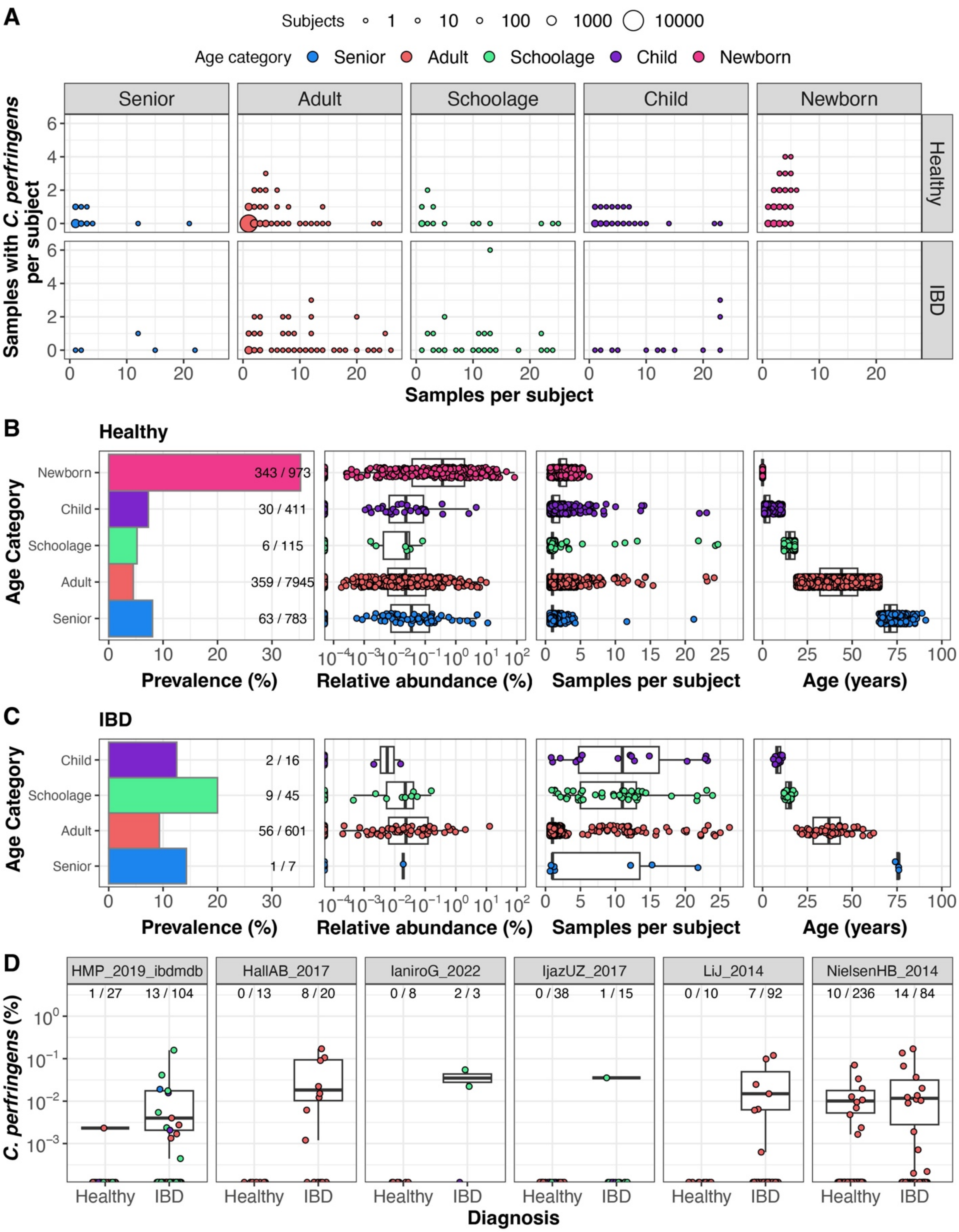

Supplementary Figure 7. *C. perfringens* abundance and prevalence in healthy subjects. Publicly available curated metagenomic datasets (77 total) were analyzed for *C. perfringens* abundance and prevalence in "healthy" or "control" subjects and IBD subjects. Subjects were grouped by age (newborn:

<1 year old, child: 1-11 y, schoolage: 12-18 y, adult: 19-65 y, senior: 65+ y). An exact age was not provided for some subjects, only age category. For a list of compiled studies, see **Supplementary Table 6**. (A) Multiple samples were collected from some subjects, without a clear correlation of more sampling leading to more *C. perfringens* detected. (B,C) *C. perfringens* prevalence, average relative abundance, number of samples collected per subject, and subject age for (B) "healthy" and "control" subjects or (C) IBD subjects. Prevalence is calculated based on detection of *C. perfringens* in any sample from a given subject. Relative abundance is the average across all samples from a subject. (D) Direct comparison of *C. perfringens* relative abundance in healthy/control vs. IBD subjects from studies with both healthy/control and IBD groups. Fraction of total samples with *C. perfringens* detected shown along the top.
(Note that different extraction methods were used for datasets, with many protocols not including bead-beating. It is also worth noting that different computational methods vary in species detection ability, where Kraken/bracken (used to analyze MI, Zeevi, etrolizumab WMS data featured in Figure 3G) is much more sensitive and less specific than MetaPhlAn3 (used for curatedMetagenomicData).)

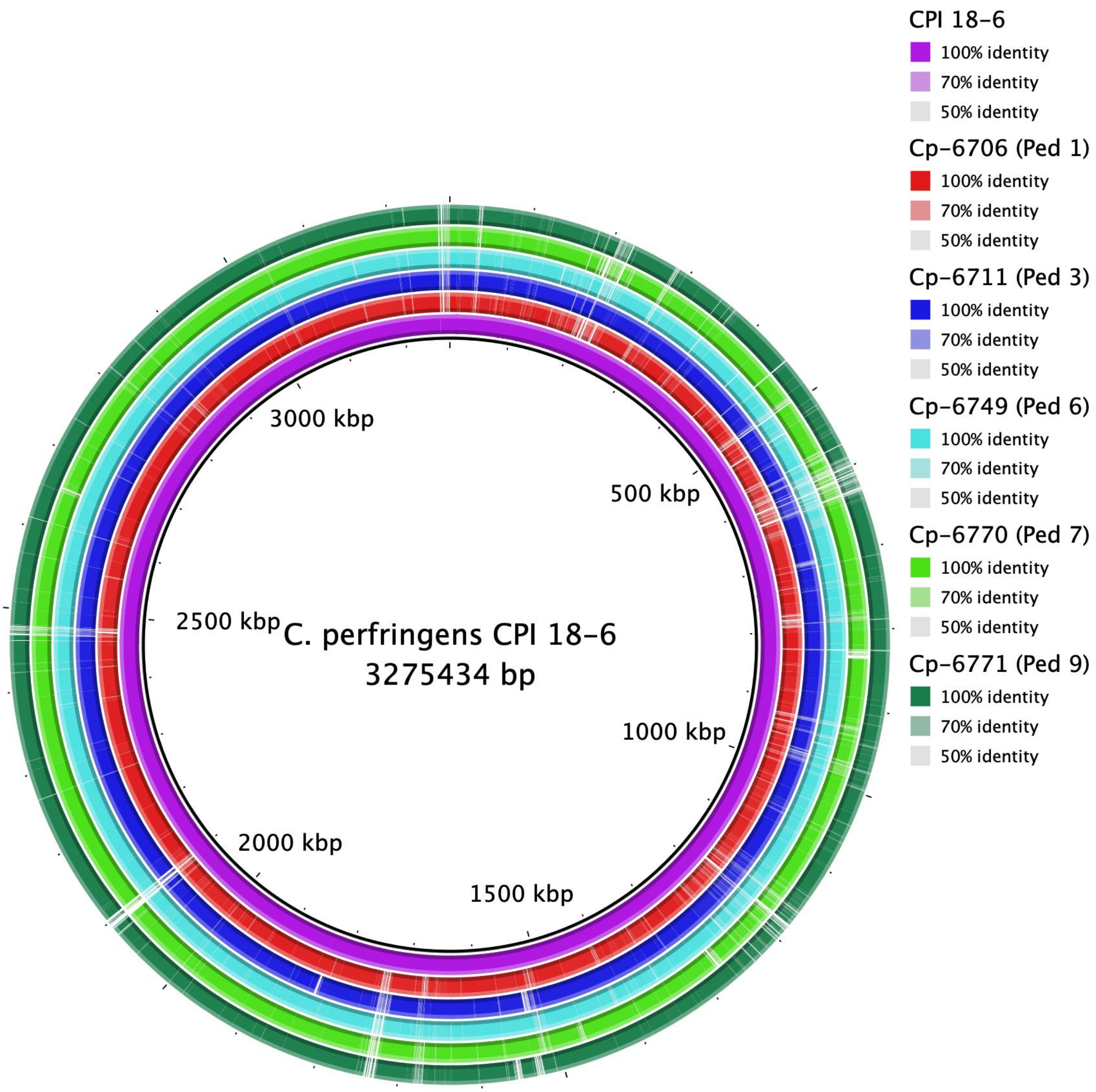

Supplementary Figure 8. Distinct genome content of the five *C. perfringens* isolates. Shown is a genome-wide BLAST comparison of *C. perfringens* genome sequences. Rings show high-scoring segment pairs (HSPs) yielded by BLASTn searches of the corresponding query *C. perfringens* genomes against reference genome *C. perfringens* CPI 18-6 with default BLAST parameter settings. This figure was produced using BRIG[22].

Clade I. (Patient 9)

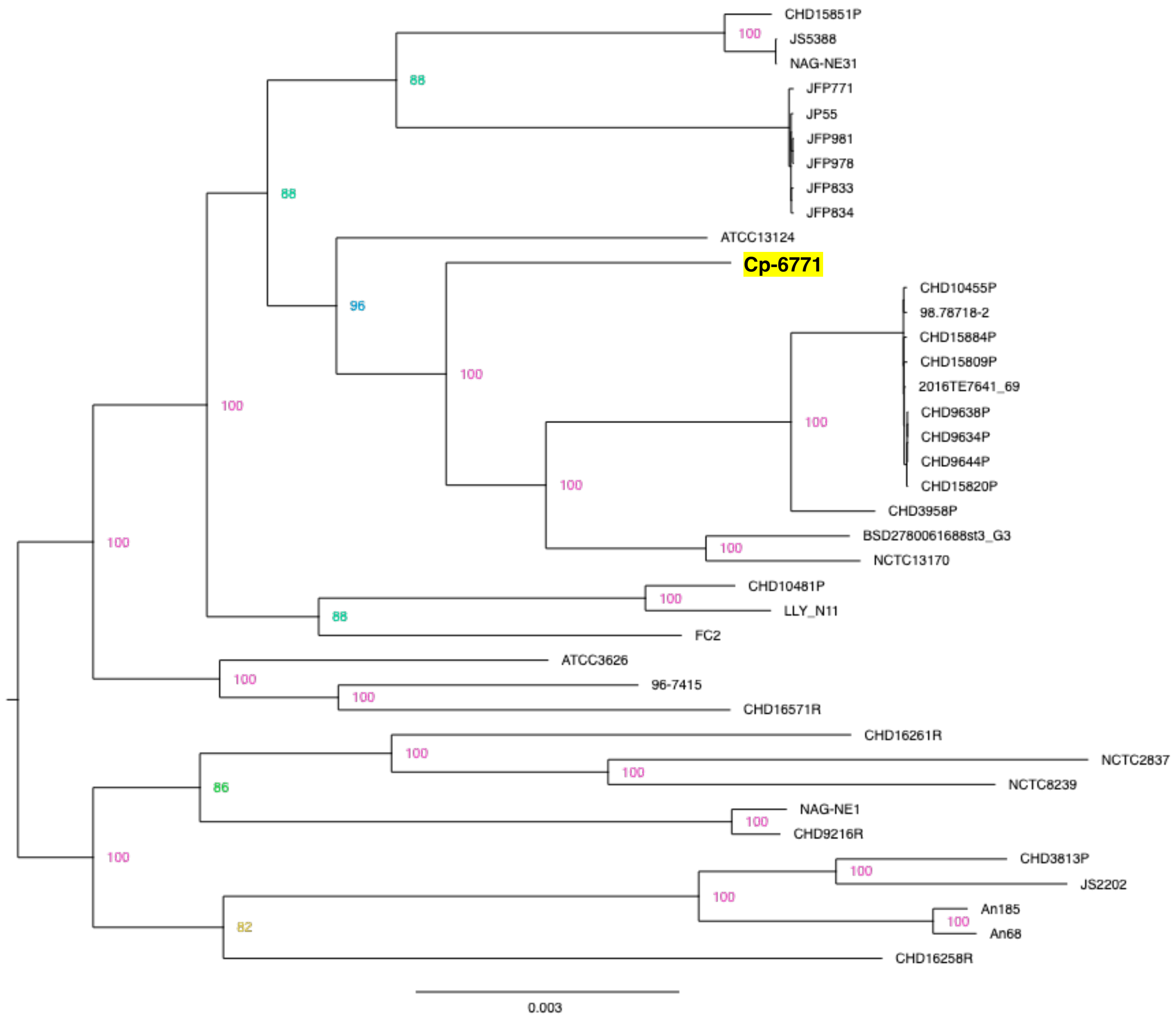

Supplementary Figure 9.1. Maximum likelihood phylogeny based on an alignment of *C. perfringens* core genes – Cp-6771. Bootstrap values are shown. Public genomes were selected for inclusion based on a prior publication[35] and *C. perfringens* isolates from the current study are highlighted.

Clade II. (Patients 3 and 7)

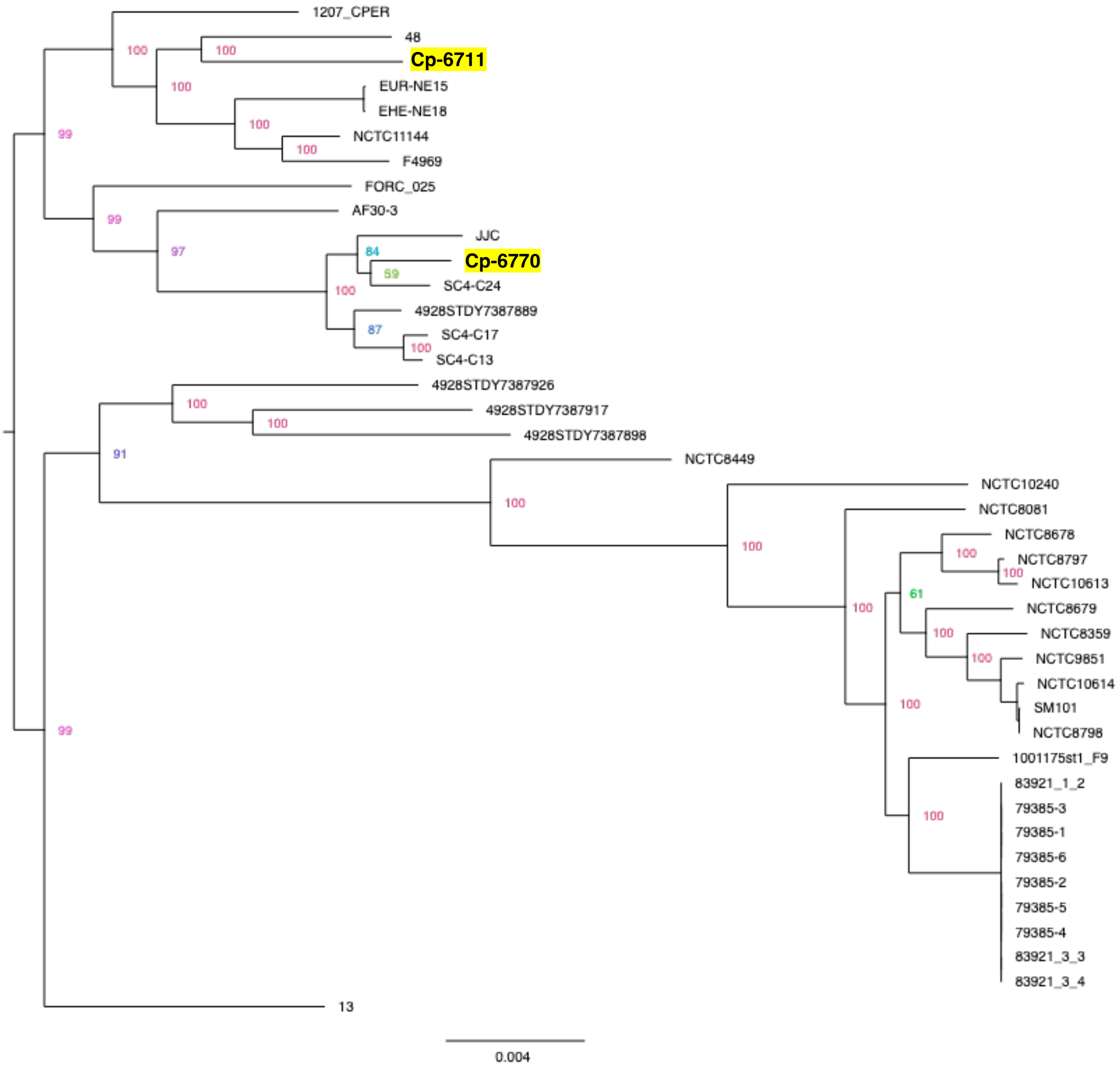

Supplementary Figure 9.2. Maximum likelihood phylogeny based on an alignment of *C. perfringens* core genes – Cp-6711 and Cp-6770. Bootstrap values are shown. Public genomes were selected for inclusion based on a prior publication[35] and *C. perfringens* isolates from the current study are highlighted.

Clade V. (Patients 1 and 6)

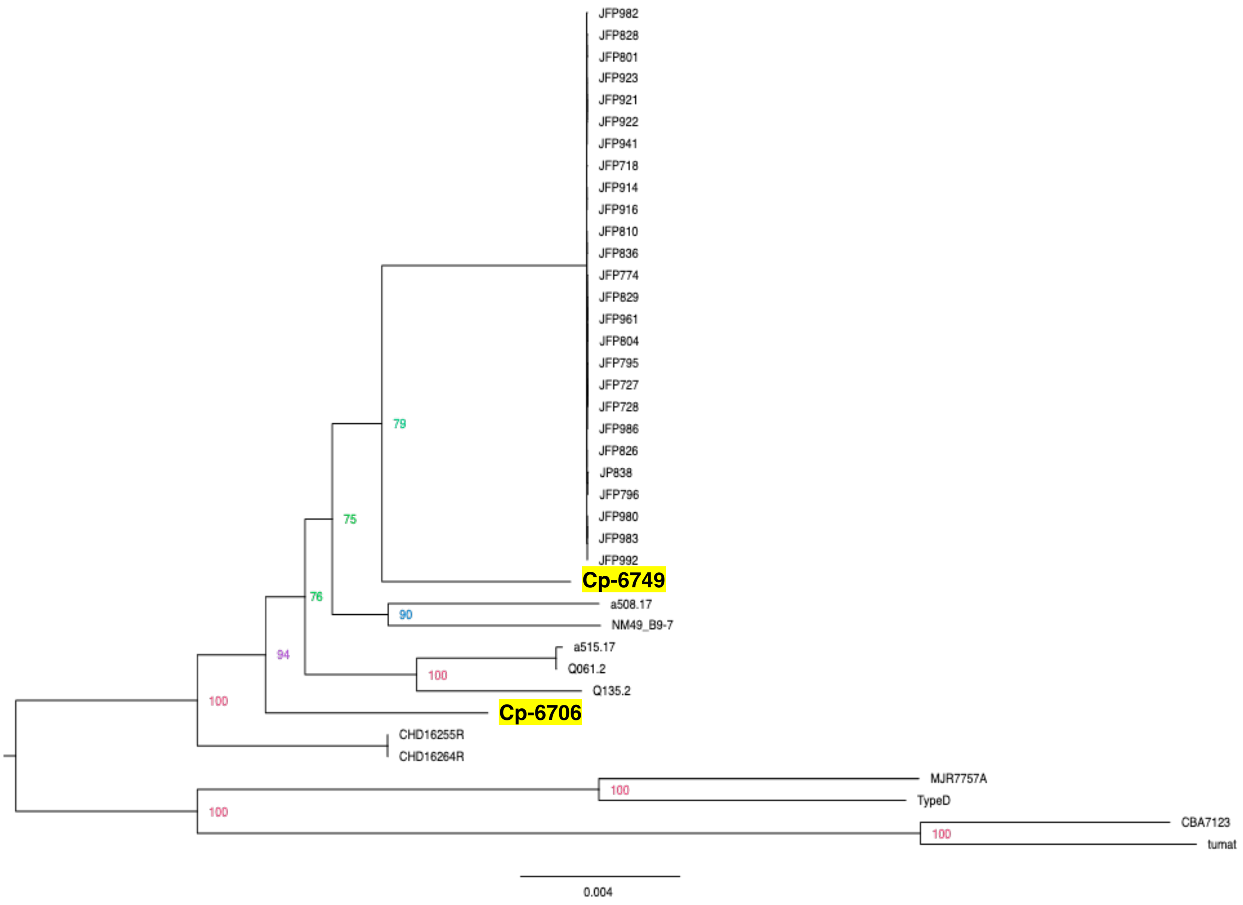

Supplementary Figure 9.3. Maximum likelihood phylogeny based on an alignment of *C. perfringens* core genes – Cp-6706 and Cp-6749. Bootstrap values are shown. Public genomes were selected for inclusion based on a prior publication[35] and *C. perfringens* isolates from the current study are highlighted.

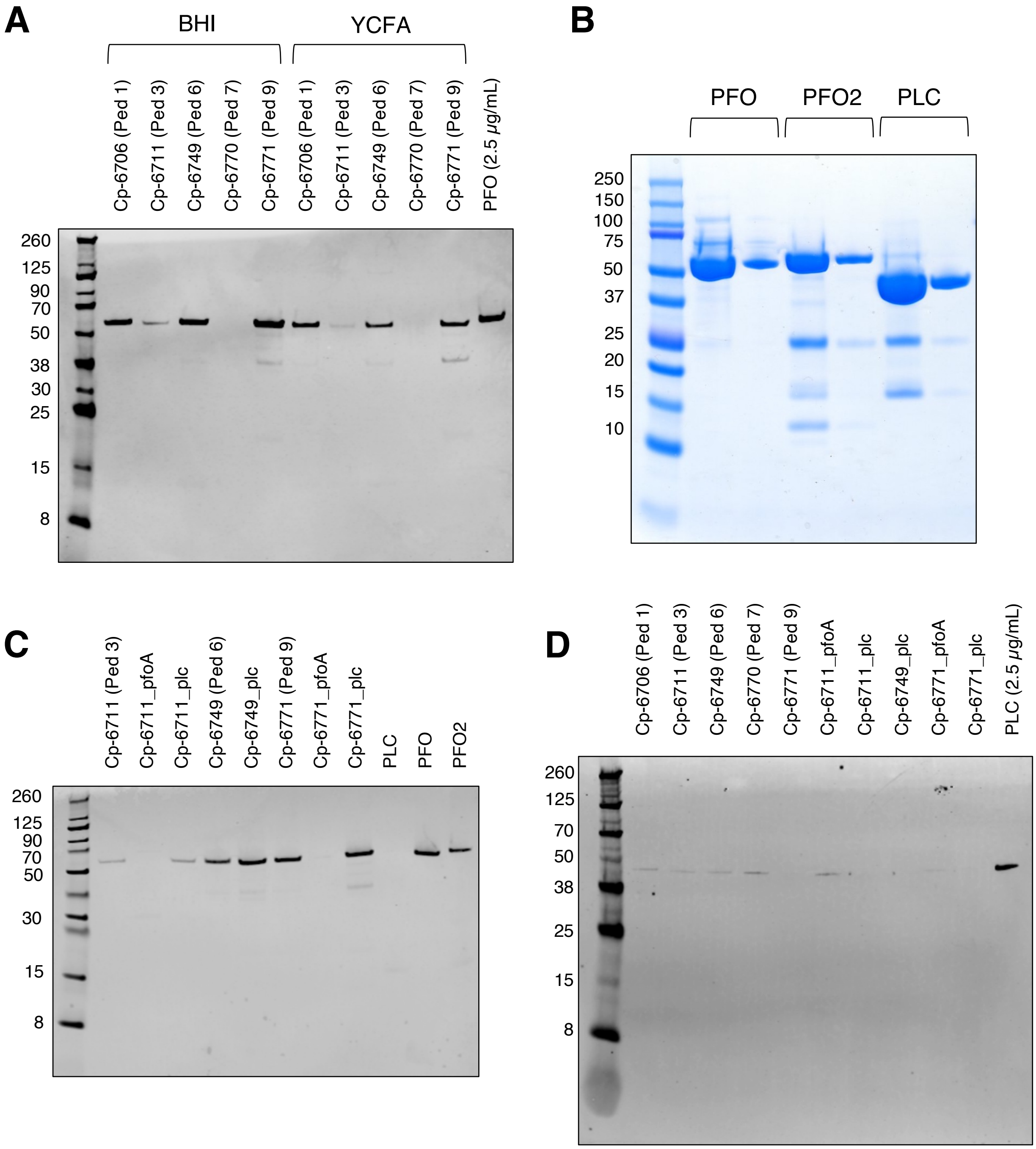

Supplementary Figure 10. Protein analysis of *C. perfringens* supernatants and purified hemolytic toxins. (A) Full Western blot of *C. perfringens* supernatants grown in BHI and YCFA probed for PFO protein levels, compared with commercial PFO protein (ATCC) at 2.5 μg/mL. Selected section shown in Figure 4B. (B) SDS-PAGE analysis of *E. coli* expressed and purified hemolytic toxins from *C. perfringens*: PFO1, perfringolysin O; PFO2, alveolysin; PLC, alpha-toxin. 10 μl (first lane) and 1 μl (second lane) of the eluted fraction were loaded. (C) Full Western blot of *C. perfringens* toxin mutant supernatants grown in BHI and purified toxins, probed for PFO protein levels. Selected section shown in Figure 4G. (D) Western blot of *C. perfringens* supernatants grown in BHI, probed for PLC protein.

A

B

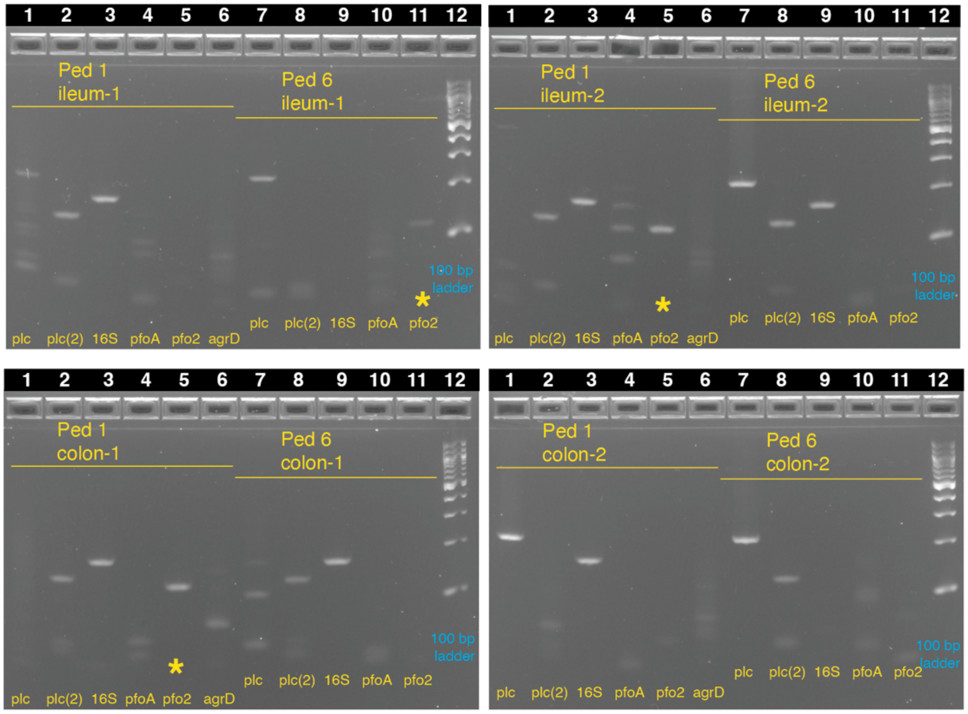

C

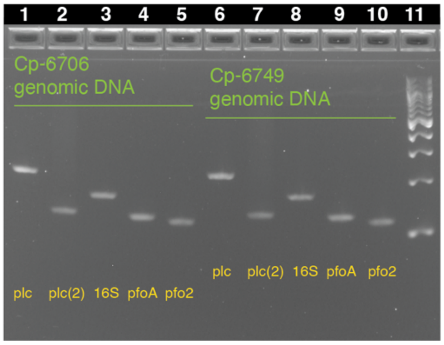

D

Gene Target	Amplicon Size (bp)	Primers
plc	219	Cper-plc508-F, Cper-plc508-R
plc(2)	135	j361, j362
16S (C. perfringens)	170	s-Clper-F, ClPER-R
pfoA (Cp-6707, Cp-6749)	131	j365, j366
pfoA (Cp-6711, Cp-6771)	124	j367, j368
pfo2 (Cp-6707)	124	j369, j370
pfo2 (Cp-6749)	122	j371, j372
agrD	76	j373, j374

Supplementary Figure 11. Biopsy cDNA PCR for *C. perfringens* genes. (A-D) PCR of biopsy cDNA using qPCR-design primers for signatures of indicated genes. RNA was extracted from biopsies, DNase-treated with two methods, and cDNA was prepared by reverse transcription. Annotated plc and plc(2) indicate different primer sets for the *plc* gene; *agrD* is a quorum sensing autoinducer peptide gene[36]. (E) PCR of genomic DNA with corresponding primers. Primers were also confirmed on cDNA from extracted RNA of pure isolate *C. perfringens* cultures grown in BHI. (F) Amplicon sizes for each gene targeted and primers used. The plc and 16S primer sets are those used in a different study[37].

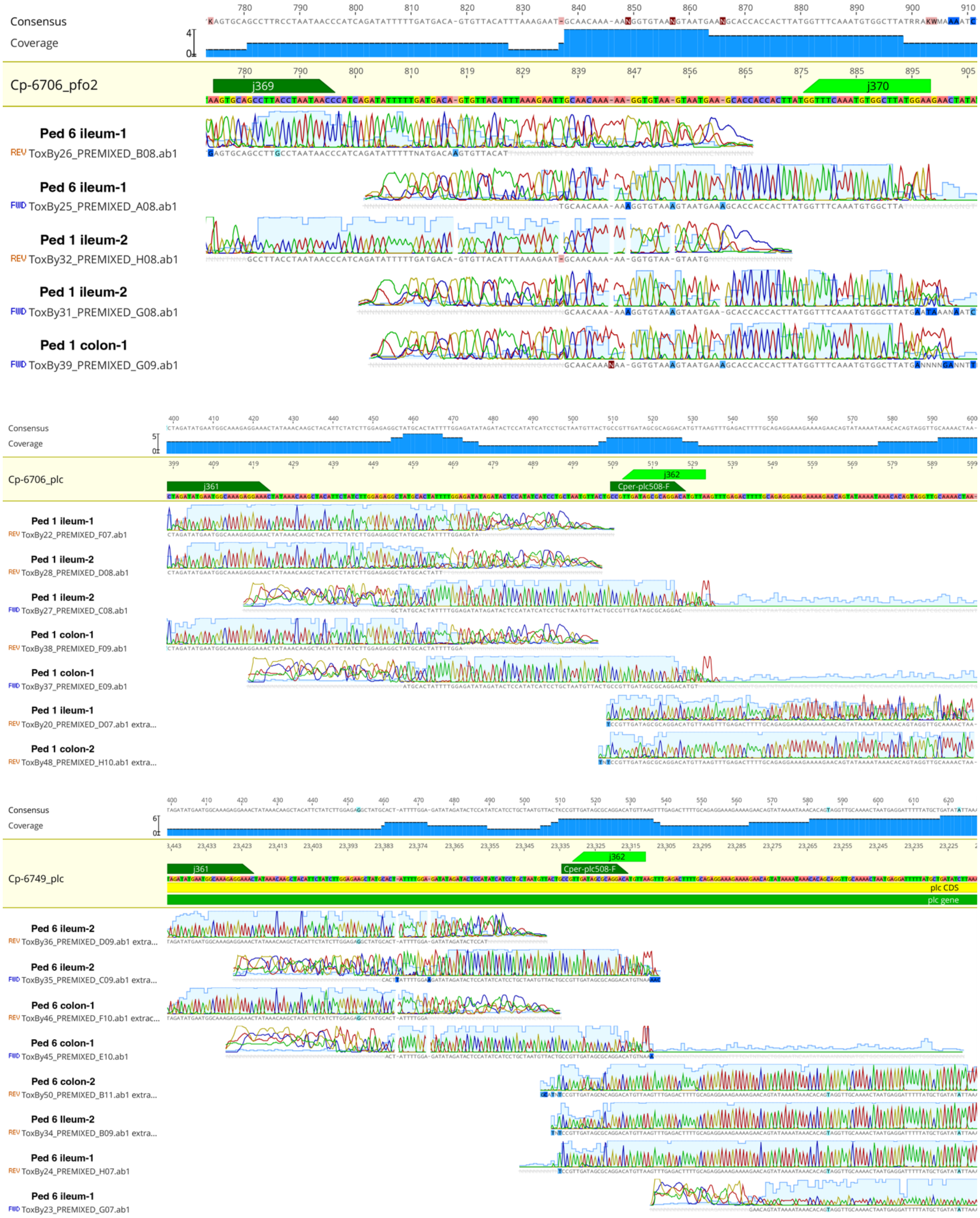

Supplementary Figure 12. Biopsy cDNA PCR Sanger sequencing results. Sequencing of (A) *pfo2* and (B) *plc* amplicons from biopsy cDNA PCR. Corresponding bands seen in **Supplementary Figure 11**. Alignment of select sequences using Geneious version 2023.2 created by Biomatters.

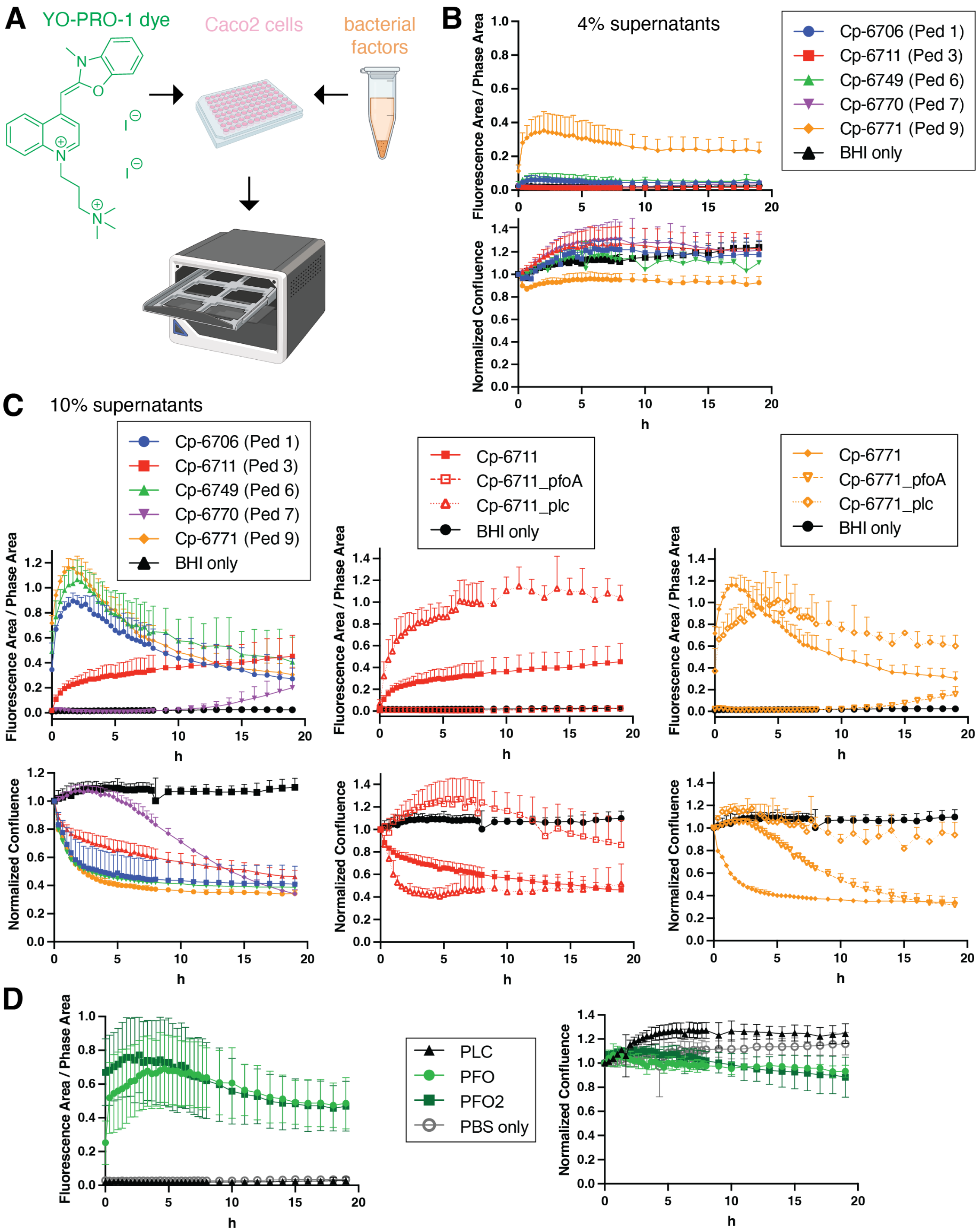

Supplementary Figure 13. Caco2 epithelial permeability is induced by *C. perfringens*. (A) Experimental setup schematic: Caco2 epithelial cells were incubated with apoptotic cell permeable fluorescent dye YO-PRO and bacterial supernatants or proteins for live imaging in an Incucyte S3, monitoring YO-PRO uptake. Individual *C. perfringens* strains were grown overnight in BHI media, and final concentrations of (B) 4% or (C) 10% bacterial supernatants were added to the Caco2 cells with YO-PRO, measuring fluorescence area as a fraction of phase contrast area, and confluence normalized to the starting confluence per field of view. (C) Measurements were on unmodified parent strains, Cp-6711 insertion mutants, and Cp-6771 insertion mutants. (D) Purified toxins were added at 800 ng/mL. Three (B-C) or four (D) biological replicate wells and two image locations within each well.

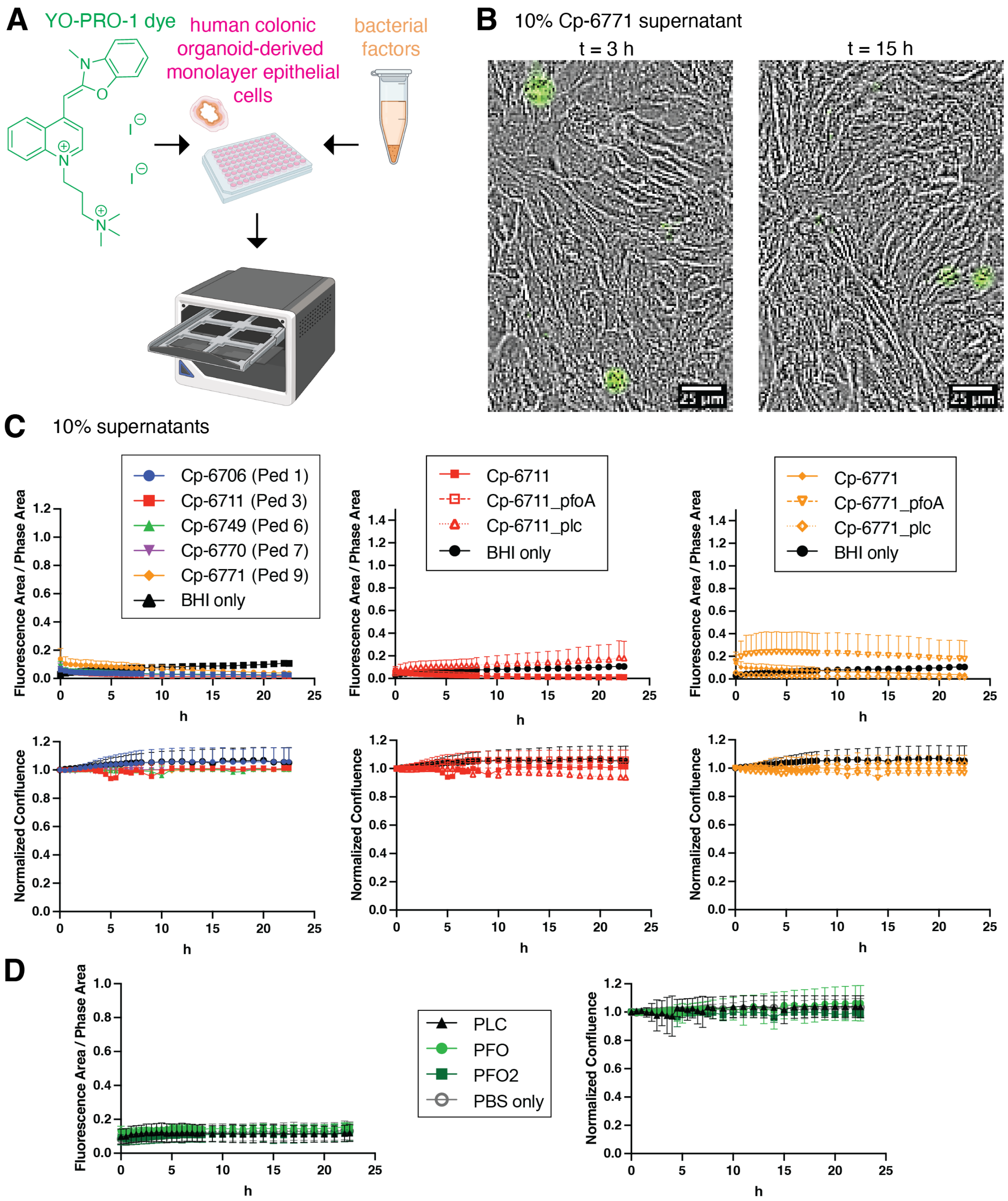

Supplementary Figure 14. Human colonic organoid-derived monolayer epithelial cells mostly resistant to *C. perfringens*. (A) Experimental setup: colonic organoid-derived monolayer epithelial cells were incubated with apoptotic cell permeable fluorescent dye YO-PRO and bacterial supernatants or proteins for live imaging in an Incucyte S3, monitoring YO-PRO uptake. Individual *C. perfringens* strains were grown overnight in BHI media, and final concentrations of 10% bacterial supernatants were added with YO-PRO, measuring fluorescence area as a fraction of phase contrast area, and confluence normalized to the starting confluence per field of view. (B) Phase contrast images with green fluorescence show monolayer of cells remains intact throughout. (C) Measurements were with unmodified parent strains, Cp-6711 insertion mutants, and Cp-6771 insertion mutants. (D) Purified toxins were added at 800 ng/mL. Three (B-C) or four (D) biological replicate wells and two image locations within each well.

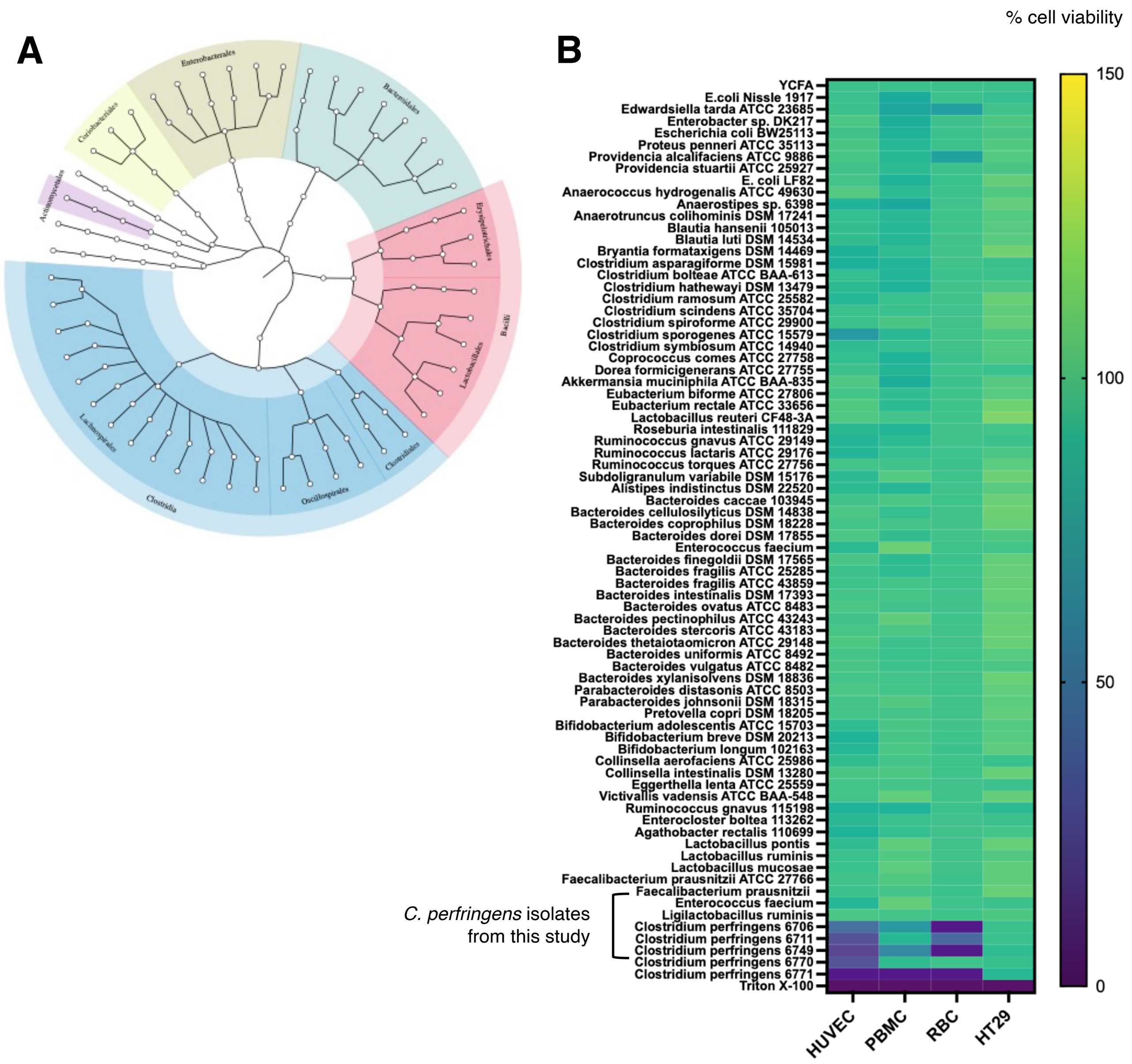

Supplementary Figure 15. Cell viability is preserved in the presence of diverse bacterial strain supernatants. (A) Taxonomic tree of 75 bacterial strains representing the broad phyla present in the human gut microbiome. Built using GraPhlAn[38]. (B) Bacterial supernatants grown in YCFA were added to HUVECs, PBMCs, HT29 epithelial cells, and RBCs for 20-24 h. Cell viability was measured by CellTiter-Glo. Relative luminescence units (RLU) were log transformed, and values were normalized to media control (100% viable) and Triton X-100 (0% viable).

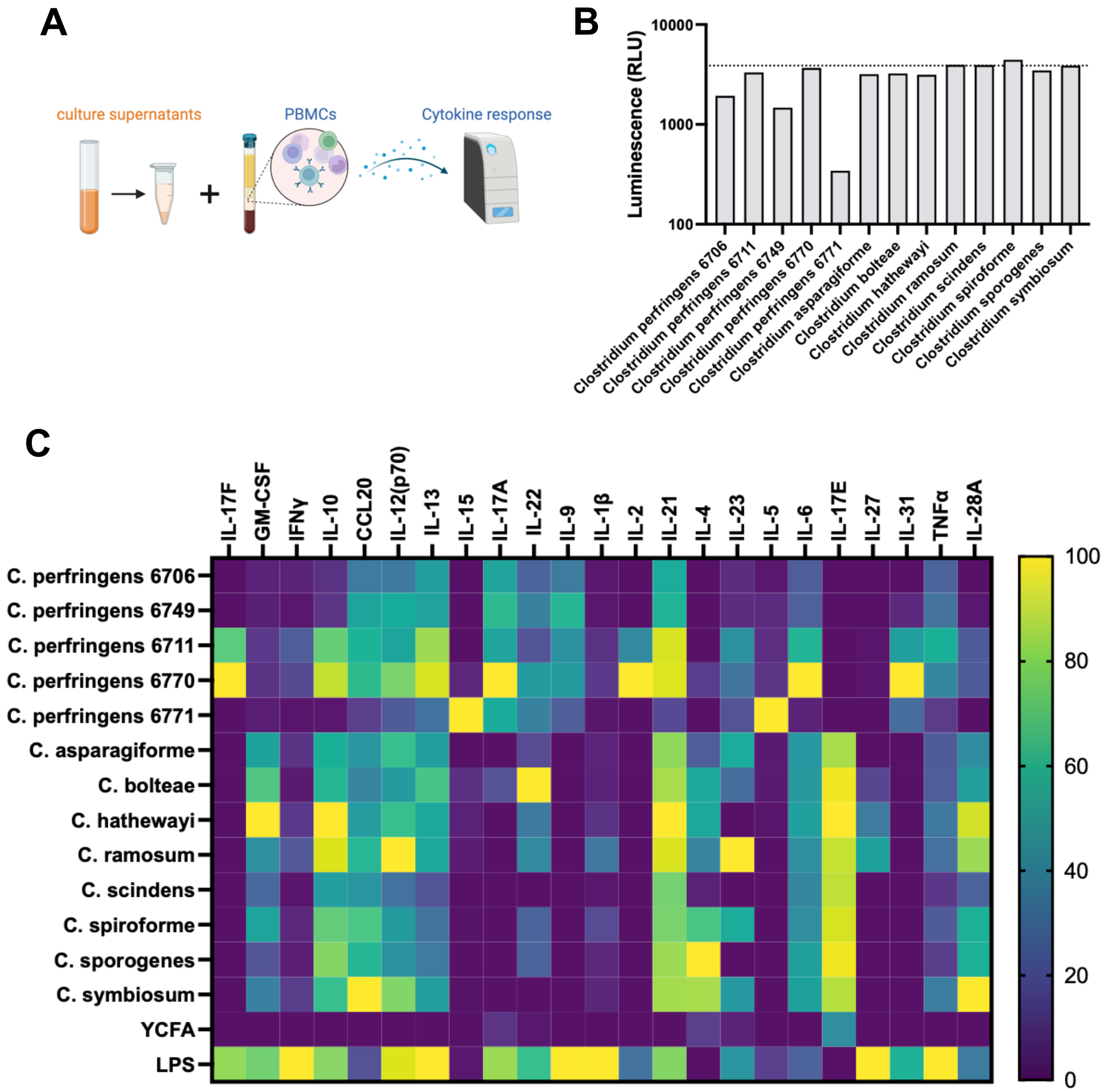

Supplementary Figure 16. Cytokine response of PBMCs stimulated with *Clostridium* supernatants. (A) Experimental setup schematic. Individual strains were grown overnight in BHI. 10% bacterial supernatants incubated with isolated peripheral blood mononuclear cells (PBMCs) for 24 hours. Cell supernatants were evaluated with the Bio-Rad 23-plus cytokine luminex assay. (B) PBMC viability was measured by CellTiter-Glo at the end of the assay. Relative light unit readings shown. (C) Cytokine responses are shown as a heat map relative to the highest and lowest values per cytokine in the dataset including YCFA culture media only (0%) and (lipopolysaccharide) LPS-stimulated (100%) controls.

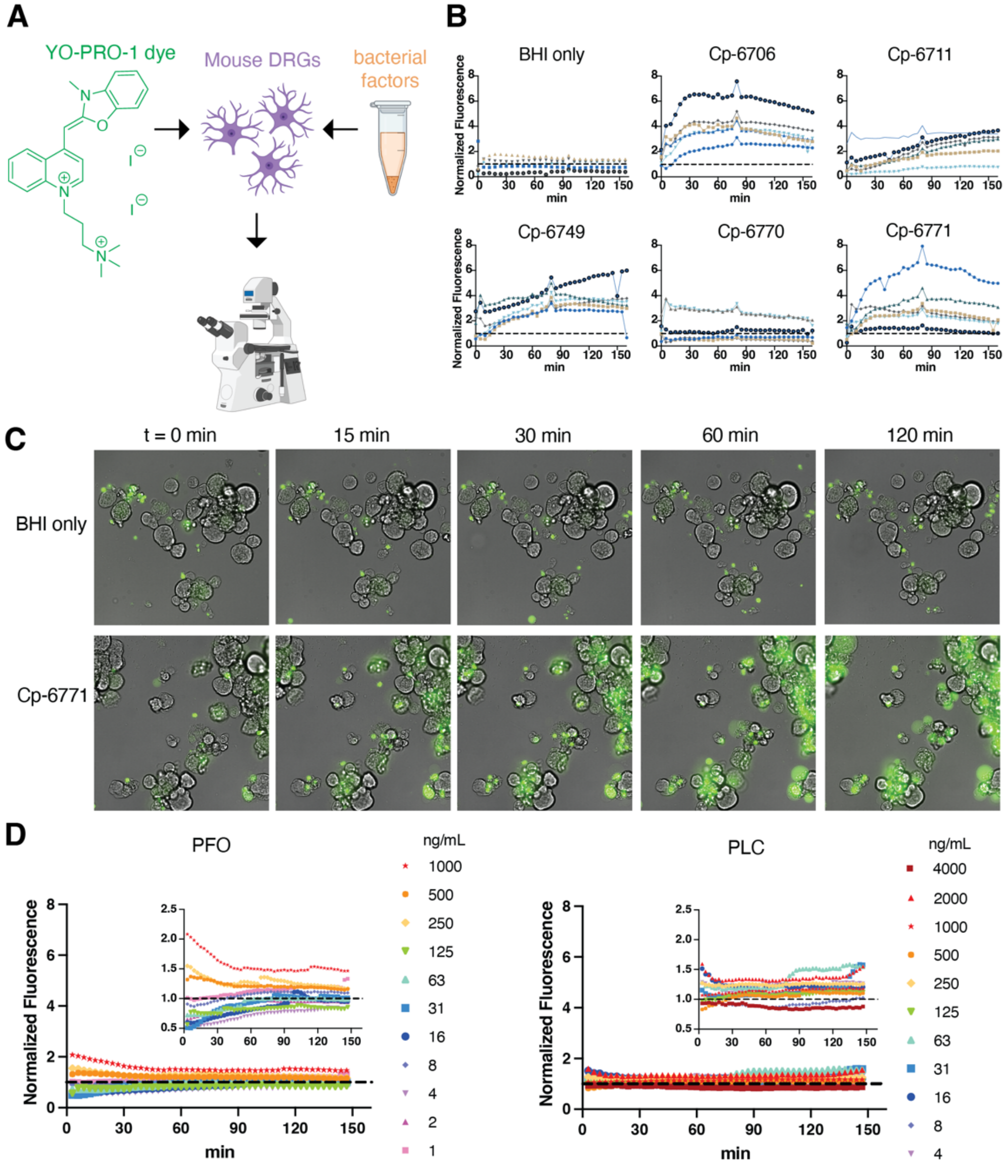

Supplementary Figure 17. *C. perfringens* supernatants trigger YO-PRO uptake in sensory neurons. (A) Experimental setup: isolated mouse DRG neurons were pre-loaded with YO-PRO before live imaging. (B) YO-PRO uptake was monitored by fluorescence microscopy. Individual *C. perfringens* strains were grown overnight in YCFA media, and final concentrations of 10% bacterial supernatants were added to the pre-loaded DRGs. Images were collected every 5 minutes. The normalized fluorescence is displayed from three biological replicates and two image locations within a single well. For each *C. perfringens* strain supernatant, the summed fluorescence intensity from cells was normalized to the average BHI media only trace at each time point. The average YCFA media is shown as a dashed line at y = 1. Wells with fewer than 20 cells were removed from analysis. (C) Representative time-lapse images with BHI media only or Cp-6771 supernatant. (D) Purified PLC and PFO toxins were added to DRG neurons pre-loaded with YO-PRO. The normalized fluorescence to the media only control (0 ng/mL) from four image locations within a single well is displayed as a dashed line at y = 1. Images were collected every 3 minutes. Insets are zoomed in on normalized fluorescence 0.5 to 2.5.

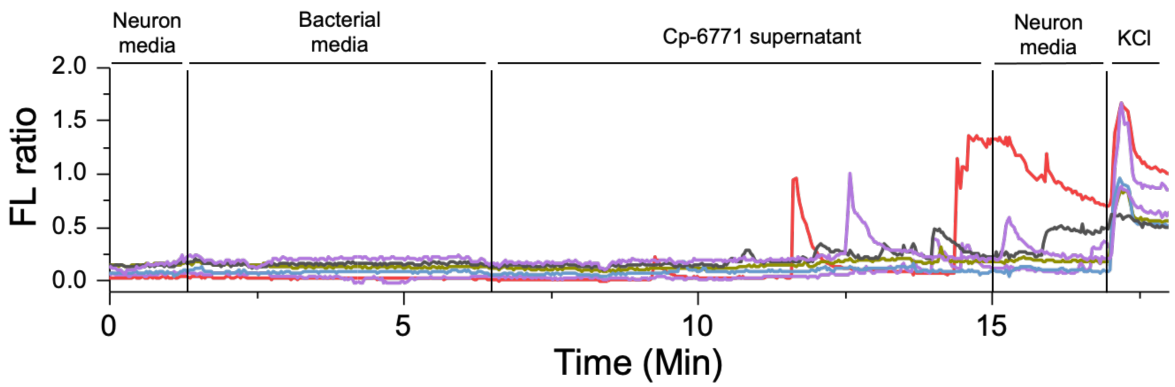

Supplementary Figure 18. *C. perfringens* supernatants activate DRG sensory neurons. Calcium signaling in mouse DRG neurons was monitored by the calcium sensitive dye, Fura-2. Neither the addition of neuronal media nor bacterial media activated DRG neurons. Addition of Cp-6771 supernatant triggered calcium signaling through membrane depolarization. The cells all responded to the positive signal from potassium chloride (KCl).

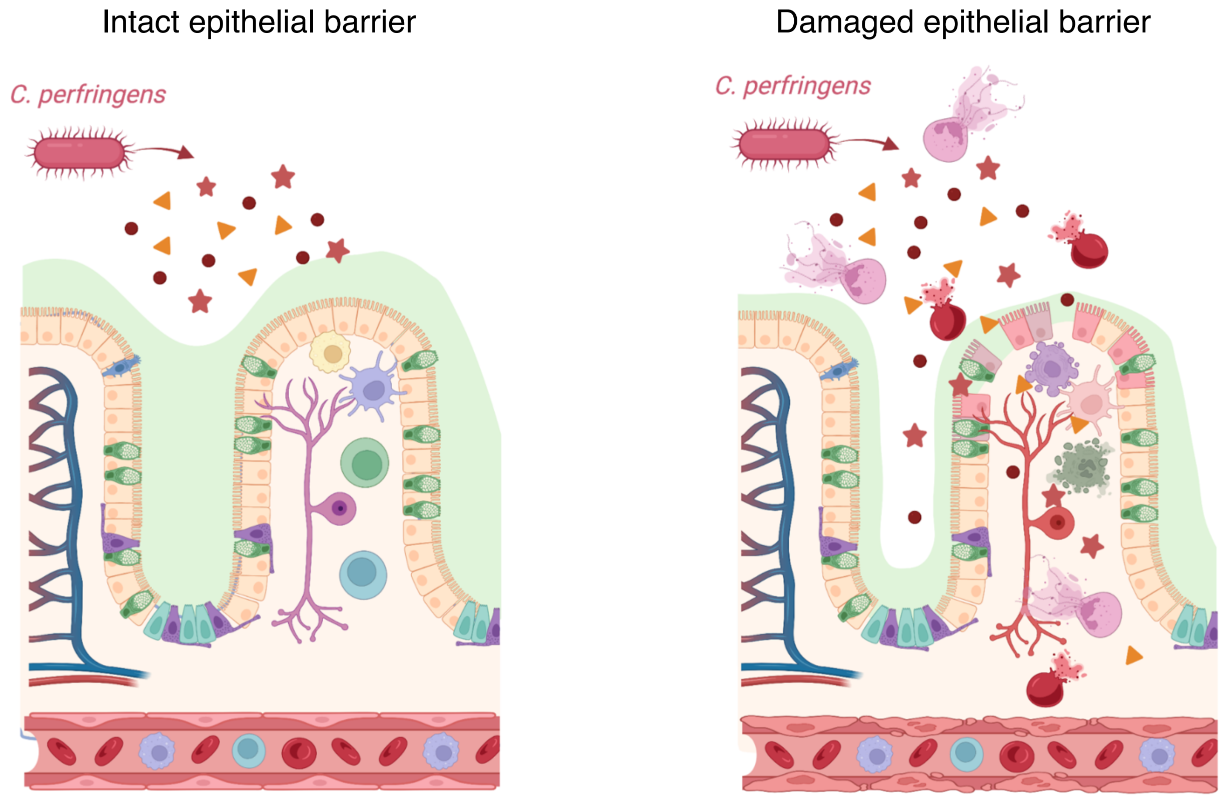

Supplementary Figure 19. A proposed model for how *C. perfringens* infection may exacerbate tissue damage in patients with impaired intestinal epithelium. The damaging potential of *C. perfringens* may depend on an intact epithelial barrier. When the epithelial barrier is intact, the effects of toxins secreted from *C. perfringens*, such as PLC (triangles), PFO (stars), and PFO2 (circles), are minimal since they are unable to gain access to the underlying sensitive cell types. When the epithelial barrier is damaged, as in cases of patients with IBD, this exposes sensitive cells (RBCs, neurons, neutrophils, PBMCs, and endothelial cells) to toxin activity, resulting in cell lysis.

SUPPLEMENTARY VIDEOS

Available online.

Supplementary Video 1. Caco2 epithelial permeability is induced by *C. perfringens.* Caco2 epithelial cells were incubated with apoptotic cell permeable fluorescent dye YO-PRO and 10% Cp-6771 bacterial supernatant for live imaging in an Incucyte S3, monitoring YO-PRO uptake. Phase contrast images with green fluorescence show monolayer of cells being damaged. *C. perfringens* strain Cp-6771 was grown overnight in BHI media.

Supplementary Video 2. Human colonic organoid-derived monolayer epithelial cells mostly resistant to *C. perfringens.* Colonic organoid-derived monolayer epithelial cells were incubated with apoptotic cell permeable fluorescent dye YO-PRO and 10% Cp-6771 bacterial supernatant for live imaging in an Incucyte S3, monitoring YO-PRO uptake. Phase contrast images with green fluorescence show monolayer of cells remains mostly intact throughout. *C. perfringens* strain Cp-6771 was grown overnight in BHI media.

SUPPLEMENTARY TABLES

Supplementary Table 1. Patient information and disease status for subjects recruited into the study.

Available online.

Supplementary Table 2. 16S V4 Sequencing classifications at the genus level from biopsies. Patient samples were sequenced directly from biopsy, using 16S V4 sequencing and classified at the genus taxonomic rank. Accompanies Supplementary Figure 1B.

Available online.

Supplementary Table 3. 16S V4 Sequencing classifications at the genus level from BHI outgrowth. Patient samples were sequenced after the second passage outgrowth in BHI media, using 16S V4 sequencing and classified at the genus taxonomic rank. Accompanies Supplementary Figure 1C.

Available online.

Supplementary Table 4. Hemolytic communities encode known hemolysins. One bacterial community grown in BHI from a biopsy from each patient was sequenced using long-read sequencing and analyzed with the virulence factor database (VFDB)[20]. Known hemolytic toxins and *C. difficile* toxins are displayed with the percent identity to the reference virulence factor is shown.

	C. perfringens			***C. difficile***		***Escherichia coli***						
**Patient**	**pfoA**	**pfo2**	**plc**	**toxA**	**toxB**	**vat**	**hylA**	**hylB**	**hylC**	**hylD**	**senB**	**espL1**
**1**	100	92.95	100	-	-	-	-	-	-	-	-	100
**2**	-	-	-	-	-	-	-	-	-	-	-	100
**3**	100	-	99.92	-	-	83.61	99.93	99.76	99.7	99.93	99.91	
**4**	-	-	-	-	-	-	-	-	-	-	-	99.95
**5**	-	-	-	-	-	81.92	-	-	-	99.91	-	100
**6**	100	92.95	100	99.83	99.99	99.88	-	-	-	-	-	100
**7**	-	-	99.92	-	-	-	-	-	-	-	-	99.79
**8**	100	-	-	-	-	-	-	-	-	-	-	
**9**	100	-	100	-	-	-	-	-	-	-	-	99.95

Supplementary Table 5. *C. perfringens* sample abundance. 16S V4 sequencing identified *C. perfringens* in patient biopsies and stool samples. *C. perfringens* abundance in patient samples (top) prior to outgrowth and (middle) after outgrowth of the second passage in BHI. Tabulated values corresponding to **Figure 3B** and **C**. (Bottom) Hemolytic activity of biopsy and stool samples outgrown in BHI expressed as percent lysis compared to the 0.5% Triton X-100 control. Tabulated values correspond to **Figure 2B**.

I. *C. perfringens* percent abundance in patient samples prior to outgrowth

	**Ped1**	**Ped2**	**Ped3**	**Ped4**	**Ped5**	**Ped6**	**Ped7**	**Ped8**	**Ped9**
**duodenum-1**	-	-	-	-	0.0	-	-	-	-
**duodenum-2**	-	-	-	-	-	-	-	-	-
**ileum-1**	22.6	0.0	-	-	0.0	26.6	0.6	-	0.2
**ileum-2**	24.9	0.0	0.0	-	0.0	31.2	-	-	0.2
**colon-1**	12.9	0.1	0.0	-	0.0	-	0.5	0.0	0.2
**colon-2**	-	0.0	0.0	0.0	0.0	29.5	-	0.0	0.1
**colonic wash**	1.2	0.0	0.0	-	8.4	1.3	-	0.0	0.1
**stool**	0.1	0.0	0.0	0.0	0.9	0.1	0.1	0.0	0.1

"-" sample had < 10,000 reads

II. *C. perfringens percent* abundance in patient samples after outgrowth in BHI

	**Ped1**	**Ped2**	**Ped3**	**Ped4**	**Ped5**	**Ped6**	**Ped7**	**Ped8**	**Ped9**
**duodenum-1**	50.5	0.0	0.0	0.0	-	0.1	-	-	-
**duodenum-2**	15.6	0.0	0.0	0.0	-	-	-	-	-
**ileum-1**	6.3	0.0	0.0	0.0	0.0	16.3	3.0	0.0	0.1
**ileum-2**	10.7	0.0	0.0	0.0	0.0	21.8	4.7	0.0	9.5
**colon-1**	31.6	0.0	3.6	0.0	0.0	22.3	2.6	0.0	44.2
**colon-2**	9.7	0.0	0.0	0.0	0.0	26.4	2.0	0.0	54.8
**colonic wash**	N/A	N/A	N/A	N/A	N/A	N/A	N/A	0.0	80.5
**stool**	N/A	N/A	N/A	N/A	N/A	N/A	N/A	0.0	3.5

"N/A" - sample type not grown out in BHI

"-" sample had < 10,000 reads

III. Hemolytic activity (%) from BHI outgrown communities

	**Ped1**	**Ped2**	**Ped3**	**Ped4**	**Ped5**	**Ped6**	**Ped7**	**Ped8**	**Ped9**
**duodenum-1**	1.5	1.7	2.1	0.2	0.5	-0.4	1.7	1.2	-0.2
**duodenum-2**	0.8	1.2	2.1	0.1	0.4	-1.1	1.7	0.4	0.4
**ileum-1**	58.2	1.5	2.4	-0.1	-0.1	85.3	13.8	-0.1	0.1
**ileum-2**	91.0	0.4	1.7	-0.2	0.6	86.7	31.3	0.0	0.3
**colon-1**	77.5	1.0	79.9	-0.3	0.0	85.9	7.5	0.0	98.1
**colon-2**	78.9	1.1	2.2	-0.3	1.9	85.0	5.5	-0.1	99.8
**colonic wash**	N/A	N/A	N/A	N/A	N/A	N/A	N/A	-1.3	96.0
**stool**	N/A	N/A	N/A	N/A	N/A	N/A	N/A	-1.2	0.0

"N/A" - sample type not grown out in BHI

Supplementary Table 6. Metagenomic datasets used in analyzing *C. perfringens* prevalence in "healthy" and "control" and IBD populations.

Available online.

Supplementary Table 7. Summary of *C. perfringens* genome quality as assessed using CheckM. All strains are marker lineage o__Clostridiales (UID1375) for the taxonomic rank of the lineage-specific marker set used to estimated genome completeness, contamination, and strain heterogeneity.

Strain	(a) # genomes	(b) # markers	(c) # marker sets	(d) 0	(d) 1	(d) 2	(d) 3	(d) 4	(d) 5+	(e) Completeness	(f) Contamination	(g) Strain heterogeneity
Cp-6706	50	332	124	0	332	0	0	0	0	100	0	0
Cp-6711	50	332	124	0	332	0	0	0	0	100	0	0
Cp-6749	50	332	124	0	332	0	0	0	0	100	0	0
Cp-6770	50	332	124	0	331	1	0	0	0	100	0.81	0
Cp- 6771	50	332	124	0	332	0	0	0	0	100	0	0

(a) number of reference genomes used to infer the lineage-specific marker set
(b) number of marker genes within the inferred lineage-specific marker set
(c) number of co-located marker sets within the inferred lineage-specific marker set
(d) *0-5+*: number of times each marker gene is identified
(e) *e*stimated completeness of genome as determined from the presence/absence of marker genes and the expected colocalization of these genes
(f) estimated contamination of genome as determined by the presence of multi-copy marker genes and the expected collocalization of these genes
(g) estimated strain heterogeneity as determined from the number of multi-copy marker pairs which exceed a specified amino acid identity threshold

Supplementary Table 8. Assembly metrics for *C. perfringens* isolate strains sequenced. Strains were sequenced by Illumina whole-genome sequencing. Compared to *C. perfringens* ATCC 13124 (NCBI RefSeq GCF_000013285.1), the type strain sequenced by others (including other *C. perfringens* strains) in a different work[35] and CPI 18-6 (NCBI Accession GCF_020138775.1).

Strain	Genome size (bp)	% GC	Genome Status	Contigs	N50 (bp)	Mean contig length (bp)	Longest contig (bp)
Cp-6706	3324615	28.0	draft	48	197007	69262	755967
Cp-6711	3239695	28.1	draft	37	248819	87559	902872
Cp-6749	3328069	28.0	draft	39	370148	85335	913122
Cp-6770	3220289	28.1	draft	46	200106	70006	331103
Cp-6771	3211615	28.1	draft	30	387197	107053	922488
ATCC 13124*	3256683	28.4	complete	-	-	-	-
CPI 18-6*	3275434	28.5	complete	-	-	-	-

*not sequenced in this work

Supplementary Table 9. *C. perfringens* isolate genomes encode diverse sets of virulence factors. *C. perfringens* isolates from each patient were individually whole genome sequenced and analyzed with the virulence factor database (VFDB)[20]. All virulence factors identified are displayed with the percent identity to the reference virulence factor is shown. List of virulence factors searched for: alpha-toxin (*plc*), beta-toxin (*cpb*), beta2-toxin (*cpb2*), *Clostridium perfringens* enterotoxin (*cpe*), epsilon toxin (*etx*), iota-toxin, necrotic enteritis toxin B (*netB*), perfringolysin O (*pfoA*), perfringolysin O-2 (*pfo2*, alveolysin), alpha-clostripain (*cloSI*), kappa-toxin (*colA*, collagenase), mu-toxin, hyaluronidases (*nagH*, *nagI*, *nagJ*, *nagK*, *nagL*), and sialidases (*nanH*, *nanI*, and *nanJ*). Toxin genes not found in any strains are not shown in the table.

Patient	Strain	*pfoA*	*pfo2*	*plc*	*cloSI*	*colA*	*nagH*	*nagI*	*nagJ*	*nagK*	*nagL*	*nanH*	*nanI*	*nanJ*
**1**	Cp-6706	100	92.95	100	100	100	100	100	100	99.74	99.97	100	100	99.97
**3**	Cp-6711	100	-	100	100	100	100	100	100	100	100	100	99.95	99.97
**6**	Cp-6749	100	92.95	100	100	100	100	100	100	100	99.73	100	100	99.97
**7**	Cp-6770	-	-	100	100	100	100	100	100	100	100	100	100	99.97
**9**	Cp-6771	100	-	100	100	100	99.94	99.92	100	100	-	100	100	99.97

Supplementary Table 10. Bacterial strains.

Name	Species	Description	Source
UCSF Pediatric Patient Isolate Strains			
Cp-6706	*Clostridium perfringens*	from Patient 1	this study
Cp-6711	*Clostridium perfringens*	from Patient 3	this study
Cp-6749	*Clostridium perfringens*	from Patient 6	this study
Cp-6770	*Clostridium perfringens*	from Patient 7	this study
Cp-6771	*Clostridium perfringens*	from Patient 9	this study
Intron-integration mutants			
Cp-6711_pfoA	*Clostridium perfringens*	pfoA intron insertion mutant, made with plasmid pJK758	this study
Cp-6711_plc	*Clostridium perfringens*	plc intron insertion mutant, made with plasmid pJK760	this study
Cp-6749_plc	*Clostridium perfringens*	plc intron insertion mutant, made with plasmid pJK761	this study
Cp-6771_pfoA	*Clostridium perfringens*	pfoA intron insertion mutant, made with plasmid pJK757	this study
Cp-6771_plc	*Clostridium perfringens*	plc intron insertion mutant, made with plasmid pJK760	this study
Conjugation Strains (E. coli)			
	Escherichia coli	CA434	Nigel Minton

Other Gut bacterial strains[39]

	Species	Strain	Source
	Bifidobacterium adolescentis	ATCC 15703	ATCC
	Bifidobacterium breve	DSM 20213	ATCC
	Bifidobacterium longum subsp. Infantis	CCUG 52486	ATCC
	Bifidobacterium ruminatum	fecal isolate	ATCC
	Collinsella aerofaciens	ATCC 25986	ATCC
	Collinsella intestinalis	DSM 13280	ATCC
	Eggerthella lenta	ATCC 25559	ATCC
	Alistipes indistinctus	DSM 22520	ATCC
	Bacteroides caccae	ATCC 43185	ATCC
	Bacteroides cellulosilyticus	DSM 14838	ATCC
	Bacteroides coprophilus	DSM 18228	ATCC
	Bacteroides dorei	DSM 17855	ATCC
	Bacteroides eggerthii	DSM 20697	ATCC
	Bacteroides finegoldii	DSM 17565	ATCC
	Bacteroides fragilis	NCTC 9343	ATCC
	Bacteroides fragilis	3397 (T10)	ATCC
	Bacteroides fragilis	T(B)9	ATCC
	Bacteroides fragilis	DS-208	ATCC
	Bacteroides fragilis	HMW 610	ATCC
	Bacteroides fragilis	HMW 615	ATCC
	Bacteroides fragilis	ATCC 43859	ATCC
	Bacteroides intestinalis	DSM 17393	ATCC
	Bacteroides ovatus	ATCC 8483	ATCC
	Bacteroides pectinophilus	ATCC 43243	ATCC
	Bacteroides stercoris	ATCC 43183	ATCC
	Bacteroides thetaiotaomicron	VPI-5482	ATCC
	Bacteroides thetaiotaomicron	3731	ATCC
	Bacteroides thetaiotaomicron	7330	ATCC
	Bacteroides uniformis	ATCC 8492	ATCC
	Bacteroides vulgatus	ATCC 8482	ATCC
	Bacteroides WH2	WH2	ATCC
	Bacteroides xylanisolvens	DSM 18836	ATCC
	Odoribacter splanchnius	fecal isolate	ATCC
	Parabacteroides distasonis	ATCC 8503	ATCC
	Parabacteroides johnsonii	DSM 18315	ATCC
	Parabacteroides merdae	ATCC 43184	ATCC
	Pretovella copri	DSM 18205	ATCC

(cont'd)	**Species**	**Strain**	**Source**
	Anaerococcus hydrogenalis	DSM 7454	ATCC
	Anaerostipes sp.	fecal isolate	ATCC
	Anaerotruncus colihominis	DSM 17241	ATCC
	Blautia hansenii	DSM 20583	ATCC
	Blautia luti	DSM 14534	ATCC
	Bryantia formataxigens	DSM 14469	ATCC
	Clostridium asparagiforme	DSM 15981	ATCC
	Clostridium bolteae	ATCC BAA-613	ATCC
	Clostridium difficile	120	ATCC
	Clostridium hathewayi	DSM 13479	ATCC
	Clostridium sp.	fecal isolate	ATCC
	Clostridium ramosum	ATCC 25582 / DSM 1402	ATCC
	Clostridium scindens	ATCC 35704	ATCC
	Clostridium spiroforme	ATCC 29900 / DSM 1552	ATCC
	Clostridium sporogenes	ATCC 15579	ATCC
	Clostridium symbiosum	ATCC 14940	ATCC
	Coprococcus comes	ATCC 27758	ATCC
	Dorea formicigenerans	ATCC 27755	ATCC
	Enterococcus faecalis	V583	ATCC
	Eubacterium biforme	DSM 3989	ATCC
	Eubacterium hallii	DSM 3353	ATCC
	Eubacterium rectale	ATCC 33656	ATCC
	Eubacterium ventriosum	ATCC 27560	ATCC
	Lactobacillus reuteri CF48-3A	BEI HM-102	ATCC
	Roseburia intestinalis	L1-82	ATCC
	Ruminococcus gnavus	ATCC 29149	ATCC
	Ruminococcus lactaris	ATCC 29176	ATCC
	Ruminococcus torques	ATCC 27756	ATCC
	Subdoligranulum variabile	DSM 15176	ATCC
	Victivallis vadensis	ATCC BAA-548	ATCC
	Edwardsiella tarda	ATCC 23685	ATCC
	Enterobacter cancerogenus	ATCC 35316	ATCC
	Escherichia coli	BW25113	ATCC
	Proteus penneri	ATCC 35198	ATCC
	Providencia alcalifaciens	DSM 30120	ATCC
	Providencia rettgeri	DSM 1131	ATCC
	Providencia stuartii	ATCC 25827	ATCC
	Salmonella typhimurium	LT2	ATCC
	Akkermansia muciniphila	ATCC BAA-835	ATCC

Supplementary Table 11. Primers used in this study.

Use	Name	DNA Sequence (5' to 3')	Source
**pGM-ACAQ plasmid assembly**			
	j241	CGCCGATGGTAGTGTGGGGTCT	this study
	j242	GATGGCTTGTAGATATGACGACAGGAAGAGTTTGTAGAAACGCA AAAAGGCC	this study
	j243	CGTCATATCTACAAGCCATCCCCCCACAGATACGGCCATTATTT TTTTGAACAAT	this study
	j244	TATACCACATTTTTGTGAATTTTTGCTTATAATCCATAACAATC ATC	this study
	j245	CTTCGCGACTCATAGAATTATTTC	this study
	j246	ATGAACAAAAATATAAAATATTCTC	this study
	j247	ATGCACTCGTAGTAGTCTGAGAAG	this study
	j248	ACATTCACTTGTGTTTATGAATCAC	this study
	j249	ACACCCTGCAGGGGGCCCGATCGG attgcgttgcgctcact	this study
	j250	CGCCGATGGTAGTGTGGGGTCTCCCC gccgcatagttaagcca	this study
	j251	AGACCCCACACTACCATCGGCG attgcgttgcgctcact	this study
	j252	CCGATCGGGCCCCCTGCAGGGTGT gccgcatagttaagcca	this study
	j253	TCAAGGTGTACTGCCTTC	this study
	j254	TCAAGAAGAGCGACTTC	this study
	j255	cgtcgttaaatgccctt	this study
	j256	TGCTCGACATTCACTTG	this study
	j268	tttattacgtggcgacg	this study
	j269	catatatggctagatcg	this study
	j270	tgtctgtaagcggatgc	this study
	j271	taatgaccccgaagcag	this study
	j272	acagaggaaagcagtat	this study
	j273	ccgatcgggccccctgca	this study
	j274	ggggagaccccacactaccatcg	this study
	j275	cgccgatggtagtgtggggtctcc	this study
	j276	acaccctgcagggggcccgat	this study
	j277	cttctcagactactacgagtg	this study
	j278	gtgattcataaacacaagtgaatgtcgag	this study
	j279	tcaaaaaaataatggccgtatctgtg	this study
	j280	ggatgattgttatggattataagcaa	this study
	j281	tctgtgacactgtcagac	this study
***C. perfringens* toxin PCR**			
plc	Cper-plc508-F	CCGTTGATAGCGCAGGACA	Nagpal et al. 2015 [37]
	Cper-plc508-R	CCCAACTATGACTCATGCTAGCA	Nagpal et al. 2015 [37]
plc(2)	j361	CTAGATATGAATGGCAAAGAGGAAAC	this study
	j362	TAACATGTCCTGCGCTATCAA	this study
16S (*C. perfringens*)	s-Clper-F	GGGGGTTTCAACACCTCC	Nagpal et al. 2015 [37]
	ClPER-R	GCAAGGGATGTCAAGTGT	Nagpal et al. 2015 [37]
pfoA (Cp-6706, Cp-6749)	j365	CAGTTGCTGCTGTTCACAATAA	this study
	j366	TGAAACTTCATCCCATGCTACT	this study
pfoA (Cp-6711, Cp-6771)	j367	CAGTTGCTGCTGTTCACAATAA	this study
	j368	TCATCCCAGGCTACTTCAAAC	this study
pfo2 (Cp-6706)	j369	AGTGCAGCCTTACCTAATAACC	this study
	j370	TTCCATAAGCCACATTTGAAACC	this study
pfo2 (Cp-6749)	j371	AGTGCAGCCTTGCCTAATAA	this study
	j372	CCATAAGCCACATTTGAAACCA	this study
agrD	j373	GCTGCATTAACAACAGTAGTTGC	this study
	j374	GTTCCTCTGGTTGGTGTGTAAA	this study

Supplementary Table 12. Sequences: Group II introns for insertion mutants of *pfoA*, *plc*. Introns in pGM-ACAQ (pCB102 origin of replication)[9].

Name	Description	DNA Sequence (5' to 3')
pJK757	Cp_pfoA intron	TTATCCTTAGAATCCATACTTGTGCGCCCAGATAGGGTGTTAAGTCAAGTAGTTTAAGGTACTACTC<br>TGTAAGATAACACAGAAAACAGCCAACCTAACCGAAAAGCGAAAGCTGATACGGGAACAGAGCACGG<br>TTGGAAAGCGATGAGTTACCTAAAGACAATCGGGTACGACTGAGTCGCAATGTTAATCAGATATAAG<br>GTATAAGTTGTGTTTACTGAACGCAAGTTTCTAATTTCGATTGATTCTCGATAGAGGAAAGTGTCTG<br>AAACCTCTAGTACAAAGAAAGGTAAGTTACAAAGTATGACT
pJK758	Cp_pfoA intron2	TTATCCTTAATTGCCATACTTGTGCGCCCAGATAGGGTGTTAAGTCAAGTAGTTTAAGGTACTACTC<br>TGTAAGATAACACAGAAAACAGCCAACCTAACCGAAAAGCGAAAGCTGATACGGGAACAGAGCACGG<br>TTGGAAAGCGATGAGTTACCTAAAGACAATCGGGTACGACTGAGTCGCAATGTTAATCAGATATAAG<br>GTATAAGTTGTGTTTACTGAACGCAAGTTTCTAATTTCGGTTGCAATCCGATAGAGGAAAGTGTCTG<br>AAACCTCTAGTACAAAGAAAGGTAAGTTAGCAAGTATGACT
pJK760	Cp_plc intron	TTATCCTTAGGAACCCATGCTGTGCGCCCAGATAGGGTGTTAAGTCAAGTAGTTTAAGGTACTACTC<br>TGTAAGATAACACAGAAAACAGCCAACCTAACCGAAAAGCGAAAGCTGATACGGGAACAGAGCACGG<br>TTGGAAAGCGATGAGTTACCTAAAGACAATCGGGTACGACTGAGTCGCAATGTTAATCAGATATAAG<br>GTATAAGTTGTGTTTACTGAACGCAAGTTTCTAATTTCGATTGTTCCTCGATAGAGGAAAGTGTCTG<br>AAACCTCTAGTACAAAGAAAGGTAAGTTAATAGCATGGACT
pJK761	Cp_plc intron2	TTATCCTTAGTTTTCGCAAAAGTGCGCCCAGATAGGGTGTTAAGTCAAGTAGTTTAAGGTACTACTC<br>TGTAAGATAACACAGAAAACAGCCAACCTAACCGAAAAGCGAAAGCTGATACGGGAACAGAGCACGG<br>TTGGAAAGCGATGAGTTACCTAAAGACAATCGGGTACGACTGAGTCGCAATGTTAATCAGATATAAG<br>GTATAAGTTGTGTTTACTGAACGCAAGTTTCTAATTTCGATTAAAACTCGATAGAGGAAAGTGTCTG<br>AAACCTCTAGTACAAAGAAAGGTAAGTTAGGTTTTGCGACT

SUPPLEMENTARY REFERENCES

1. Javdan B, Lopez JG, Chankhamjon P, Lee YCJ, Hull R, Wu Q, Wang X, Chatterjee S, Donia MS. Personalized Mapping of Drug Metabolism by the Human Gut Microbiome. *Cell*. 2019;181(7):1661-1679.e22. doi:10.1016/j.cell.2020.05.001

2. Ng KM, Pannu S, Liu S, Burckhardt JC, Hughes T, Treuren WV, Nguyen J, Naqvi K, Nguyen B, Clayton CA, Pepin DM, Collins SR, Tropini C. Single-strain behavior predicts responses to environmental pH and osmolality in the gut microbiota. *mBio*. Published online 2023:e00753-23. doi:10.1128/mbio.00753-23

3. Ladirat SE, Schols HA, Nauta A, Schoterman MHC, Keijser BJF, Montijn RC, Gruppen H, Schuren FHJ. High-throughput analysis of the impact of antibiotics on the human intestinal microbiota composition. *J Microbiol Methods*. 2013;92(3):387-397. doi:10.1016/j.mimet.2012.12.011

4. Abbeele PV den, Grootaert C, Marzorati M, Possemiers S, Verstraete W, Gérard P, Rabot S, Bruneau A, Aidy SE, Derrien M, Zoetendal E, Kleerebezem M, Smidt H, Wiele TV de. Microbial community development in a dynamic gut model is reproducible, colon region specific, and selective for Bacteroidetes and Clostridium cluster IX. *Appl Environ Microbiol*. 2010;76(15):5237-5246. doi:10.1128/aem.00759-10

5. Jalili-Firoozinezhad S, Gazzaniga FS, Calamari EL, Camacho DM, Fadel CW, Bein A, Swenor B, Nestor B, Cronce MJ, Tovaglieri A, Levy O, Gregory KE, Breault DT, Cabral JMS, Kasper DL, Novak R, Ingber DE. A complex human gut microbiome cultured in an anaerobic intestine-on-a-chip. *Nat Biomed Eng*. 2019;3(7):520-531. doi:10.1038/s41551-019-0397-0

6. Wang K, Zhang Z, Hang J, Liu J, Guo F, Ding Y, Li M, Nie Q, Lin J, Zhuo Y, Sun L, Luo X, Zhong Q, Ye C, Yun C, Zhang Y, Wang J, Bao R, Pang Y, Wang G, Gonzalez FJ, Lei X, Qiao J, Jiang C. Microbial-host-isozyme analyses reveal microbial DPP4 as a potential antidiabetic target. *Science*. 2023;381(6657):eadd5787. doi:10.1126/science.add5787

7. Adcock PW, Saint CP. Rapid confirmation of Clostridium perfringens by using chromogenic and fluorogenic substrates. *Appl Environ Microb*. 2001;67(9):4382-4384. doi:10.1128/aem.67.9.4382-4384.2001

8. Heap JT, Kuehne SA, Ehsaan M, Cartman ST, Cooksley CM, Scott JC, Minton NP. The ClosTron: Mutagenesis in Clostridium refined and streamlined. *J Microbiol Methods*. 2010;80(1):49-55. doi:10.1016/j.mimet.2009.10.018

9. Jin WB, Li TT, Huo D, Qu S, Li XV, Arifuzzaman M, Lima SF, Shi HQ, Wang A, Putzel GG, Longman RS, Artis D, Guo CJ. Genetic manipulation of gut microbes enables single-gene interrogation in a complex microbiome. *Cell*. 2022;185(3):547-562.e22. doi:10.1016/j.cell.2021.12.035

10. Caporaso JG, Lauber CL, Walters WA, Berg-Lyons D, Huntley J, Fierer N, Owens SM, Betley J, Fraser L, Bauer M, Gormley N, Gilbert JA, Smith G, Knight R. Ultra-high-throughput

microbial community analysis on the Illumina HiSeq and MiSeq platforms. *Isme J*. 2012;6(8):1621-1624. doi:10.1038/ismej.2012.8

11. Mar JS, Ota N, Pokorzynski ND, Peng Y, Jaochico A, Sangaraju D, Skippington E, Lekkerkerker AN, Rothenberg ME, Tan MW, Yi T, Keir ME. IL-22 alters gut microbiota composition and function to increase aryl hydrocarbon receptor activity in mice and humans. *Microbiome*. 2023;11(1):47. doi:10.1186/s40168-023-01486-1

12. Bolyen E, Rideout JR, Dillon MR, Bokulich NA, Abnet CC, Al-Ghalith GA, Alexander H, Alm EJ, Arumugam M, Asnicar F, Bai Y, Bisanz JE, Bittinger K, Brejnrod A, Brislawn CJ, Brown CT, Callahan BJ, Caraballo-Rodríguez AM, Chase J, Cope EK, Silva RD, Diener C, Dorrestein PC, Douglas GM, Durall DM, Duvallet C, Edwardson CF, Ernst M, Estaki M, Fouquier J, Gauglitz JM, Gibbons SM, Gibson DL, Gonzalez A, Gorlick K, Guo J, Hillmann B, Holmes S, Holste H, Huttenhower C, Huttley GA, Janssen S, Jarmusch AK, Jiang L, Kaehler BD, Kang KB, Keefe CR, Keim P, Kelley ST, Knights D, Koester I, Kosciolek T, Kreps J, Langille MGI, Lee J, Ley R, Liu YX, Loftfield E, Lozupone C, Maher M, Marotz C, Martin BD, McDonald D, McIver LJ, Melnik AV, Metcalf JL, Morgan SC, Morton JT, Naimey AT, Navas-Molina JA, Nothias LF, Orchanian SB, Pearson T, Peoples SL, Petras D, Preuss ML, Pruesse E, Rasmussen LB, Rivers A, Robeson MS, Rosenthal P, Segata N, Shaffer M, Shiffer A, Sinha R, Song SJ, Spear JR, Swafford AD, Thompson LR, Torres PJ, Trinh P, Tripathi A, Turnbaugh PJ, Ul-Hasan S, Hooft JJJ van der, Vargas F, Vázquez-Baeza Y, Vogtmann E, et al. Reproducible, interactive, scalable and extensible microbiome data science using QIIME 2. *Nat Biotechnol*. 2019;37(8):852-857. doi:10.1038/s41587-019-0209-9

13. Wagner F, Mansfield JC, Lekkerkerker AN, Wang Y, Keir M, Dash A, Butcher B, Harder B, Orozco LD, Mar JS, Chen H, Rothenberg ME. Dose escalation randomised study of efmarodocokin alfa in healthy volunteers and patients with ulcerative colitis. *Gut*. 2023;72(8):1451-1461. doi:10.1136/gutjnl-2022-328387

14. Callahan BJ, McMurdie PJ, Rosen MJ, Han AW, Johnson AJA, Holmes SP. DADA2: High resolution sample inference from Illumina amplicon data. *Nat methods*. 2016;13(7):581-583. doi:10.1038/nmeth.3869

15. Parks DH, Chuvochina M, Chaumeil PA, Rinke C, Mussig AJ, Hugenholtz P. A complete domain-to-species taxonomy for Bacteria and Archaea. *Nat Biotechnol*. 2020;38(9):1079-1086. doi:10.1038/s41587-020-0501-8

16. Parks DH, Chuvochina M, Waite DW, Rinke C, Skarshewski A, Chaumeil PA, Hugenholtz P. A standardized bacterial taxonomy based on genome phylogeny substantially revises the tree of life. *Nat Biotechnol*. 2018;36(10):996-1004. doi:10.1038/nbt.4229

17. Reeder J, Huang M, Kaminker JS, Paulson JN. MicrobiomeExplorer: an R package for the analysis and visualization of microbial communities. *Bioinformatics*. 2020;37(9):1317-1318. doi:10.1093/bioinformatics/btaa838

18. Wickham H, Averick M, Bryan J, Chang W, McGowan L, François R, Grolemund G, Hayes A, Henry L, Hester J, Kuhn M, Pedersen T, Miller E, Bache S, Müller K, Ooms J, Robinson D, Seidel D, Spinu V, Takahashi K, Vaughan D, Wilke C, Woo K, Yutani H. Welcome to the Tidyverse. *J Open Source Softw*. 2019;4(43):1686. doi:10.21105/joss.01686

19. Kolmogorov M, Bickhart DM, Behsaz B, Gurevich A, Rayko M, Shin SB, Kuhn K, Yuan J, Polevikov E, Smith TPL, Pevzner PA. metaFlye: scalable long-read metagenome assembly using repeat graphs. *Nat Methods*. 2020;17(11):1103-1110. doi:10.1038/s41592-020-00971-x

20. Chen L, Yang J, Yu J, Yao Z, Sun L, Shen Y, Jin Q. VFDB: a reference database for bacterial virulence factors. *Nucleic Acids Res*. 2005;33(suppl_1):D325-D328. doi:10.1093/nar/gki008

21. Bankevich A, Nurk S, Antipov D, Gurevich AA, Dvorkin M, Kulikov AS, Lesin VM, Nikolenko SI, Pham S, Prjibelski AD, Pyshkin AV, Sirotkin AV, Vyahhi N, Tesler G, Alekseyev MA, Pevzner PA. SPAdes: A New Genome Assembly Algorithm and Its Applications to Single-Cell Sequencing. *J Comput Biol*. 2012;19(5):455-477. doi:10.1089/cmb.2012.0021

22. Alikhan NF, Petty NK, Zakour NLB, Beatson SA. BLAST Ring Image Generator (BRIG): simple prokaryote genome comparisons. *BMC Genom*. 2011;12(1):402-402. doi:10.1186/1471-2164-12-402

23. Seemann T. Prokka: rapid prokaryotic genome annotation. *Bioinformatics*. 2014;30(14):2068-2069. doi:10.1093/bioinformatics/btu153

24. Parks DH, Imelfort M, Skennerton CT, Hugenholtz P, Tyson GW. CheckM: assessing the quality of microbial genomes recovered from isolates, single cells, and metagenomes. *Genome Res*. 2015;25(7):1043-1055. doi:10.1101/gr.186072.114

25. Page AJ, Cummins CA, Hunt M, Wong VK, Reuter S, Holden MTG, Fookes M, Falush D, Keane JA, Parkhill J. Roary: rapid large-scale prokaryote pan genome analysis. *Bioinformatics*. 2015;31(22):3691-3693. doi:10.1093/bioinformatics/btv421

26. Stamatakis A. RAxML version 8: a tool for phylogenetic analysis and post-analysis of large phylogenies. *Bioinformatics*. 2014;30(9):1312-1313. doi:10.1093/bioinformatics/btu033

27. Fujii M, Matano M, Toshimitsu K, Takano A, Mikami Y, Nishikori S, Sugimoto S, Sato T. Human Intestinal Organoids Maintain Self-Renewal Capacity and Cellular Diversity in Niche-Inspired Culture Condition. *Cell Stem Cell*. 2018;23(6):787-793.e6. doi:10.1016/j.stem.2018.11.016

28. Jung M, Dourado M, Maksymetz J, Jacobson A, Laufer BI, Baca M, Foreman O, Hackos DH, Riol-Blanco L, Kaminker JS. Cross-species transcriptomic atlas of dorsal root ganglia reveals species-specific programs for sensory function. *Nat Commun*. 2023;14(1):366. doi:10.1038/s41467-023-36014-0

29. Byrd AL, Liu M, Fujimura KE, Lyalina S, Nagarkar DR, Charbit B, Bergstedt J, Patin E, Harrison OJ, Quintana-Murci L, Mellman I, Duffy D, Albert ML, Consortium MI. Gut microbiome stability and dynamics in healthy donors and patients with non-gastrointestinal cancers. *J Exp Med*. 2020;218(1):e20200606. doi:10.1084/jem.20200606

30. Zeevi D, Korem T, Zmora N, Israeli D, Rothschild D, Weinberger A, Ben-Yacov O, Lador D, Avnit-Sagi T, Lotan-Pompan M, Suez J, Mahdi JA, Matot E, Malka G, Kosower N, Rein M, Zilberman-Schapira G, Dohnalová L, Pevsner-Fischer M, Bikovsky R, Halpern Z, Elinav E,

Segal E. Personalized Nutrition by Prediction of Glycemic Responses. *Cell*. 2015;163(5):1079-1094. doi:10.1016/j.cell.2015.11.001

31. Sandborn WJ, Panés J, Danese S, Sharafali Z, Hassanali A, Jacob-Moffatt R, Eden C, Daperno M, Valentine JF, Laharie D, Baía C, Atreya R, Panaccione R, Rydzewska G, Aguilar H, Vermeire S, Group BS. Etrolizumab as induction and maintenance therapy in patients with moderately to severely active Crohn's disease (BERGAMOT): a randomised, placebo-controlled, double-blind, phase 3 trial. *Lancet Gastroenterol Hepatol*. 2023;8(1):43-55. doi:10.1016/s2468-1253(22)00303-x

32. Vermeire S, Lakatos PL, Ritter T, Hanauer S, Bressler B, Khanna R, Isaacs K, Shah S, Kadva A, Tyrrell H, Oh YS, Tole S, Chai A, Pulley J, Eden C, Zhang W, Feagan BG, Group LS, Abraham P, Júnior MAC, Aguilar H, Ahmed T, Altorjay I, Andersen V, Arai R, Arnold H, Ausk K, Axler J, Ayub K, Balekuduru A, Neto GB, Bassan I, Behm B, Bekal P, Bhatia S, Bod B, Mello CEB, Brandeburova J, Breedt J, Bressler B, Chopey I, Connor M, Corlin R, Hernandez CAC, De A, Rolim A de S, Boratto SDF, Dixon T, Poli DD, Dresner D, Vall GAD, Ebert M, Ehehalt R, Ertan A, Valencia RE, Etzel J, Fallingborg J, Feagan B, Fedurco M, Castro EF, Borges VF de A, Finklestein M, Fischer A, Fleisher M, Rendon ARF, Fogel R, Junior OF, Freedland C, Gatof D, Gill K, Glerup H, Gokak V, Goldin E, Jaramillo HAG, Gupta N, Gurzo Z, Gyrina O, Habeeb MA, Hanauer S, Hardi R, Harlan W, Hemaidan A, Heyman M, Hoffmann P, Holderman W, Holtkamp-Endemann F, Horvat G, Isaacs K, Israeli E, Miszputen SJ, Jensen S, Johnson K, Jones J, Junior O, Kadleckova B, Kalla M, Kallo Z, Karyotakis N, et al. Etrolizumab for maintenance therapy in patients with moderately to severely active ulcerative colitis (LAUREL): a randomised, placebo-controlled, double-blind, phase 3 study. *Lancet Gastroenterol Hepatol*. 2022;7(1):28-37. doi:10.1016/s2468-1253(21)00295-8

33. Rubin DT, Dotan I, DuVall A, Bouhnik Y, Radford-Smith G, Higgins PDR, Mishkin DS, Arrisi P, Scalori A, Oh YS, Tole S, Chai A, Chamberlain-James K, Lacey S, McBride J, Panés J, Rustem A, Norasiah AB, Humberto A, Diego A, Hale A, Evangelos A, Olga A, Bagdadi A, Andres A, Ashwin A, Jane A, Tomasz A, Nathan A, Ozlen A, Mauro B, Jozef B, George B, Marko B, Andrey B, Guerino BN, Metin B, Curtis B, Stefan B, William B, Fatih B, Sudhir B, Andrzej B, Leonid B, Bahri B, Pavol B, Verle B, Julia B, Vladimir B, Francisco BP, Yoram B, James B, Tetiana B, Igor B, Ivan B, Jonathon C, Azlida CA, Tatiana C, Michael C, Ivan C, Dimitrios C, Shan CP, Galina C, Andrew C, Robert C, Mirjana C, Ulku D, Cezary DW, Olena D, Henry D, Elena D, Jelena D, Nik DJ, Julia D, Oleg D, Tomas D, Piotr D, Gerald D, Pedro DG, Aaron DG, Mikhail D, Craig E, Yusuf E, Galyna F, Oleksandr F, Olga F, Miroslav F, Roland F, Jorge F, Lorena FM, Lucky F, Bradley F, Keith F, Sergio F, Beata GW, Leonel GPF, Daniel GE, Nataliia G, et al. Etrolizumab versus adalimumab or placebo as induction therapy for moderately to severely active ulcerative colitis (HIBISCUS): two phase 3 randomised, controlled trials. *Lancet Gastroenterol Hepatol*. 2022;7(1):17-27. doi:10.1016/s2468-1253(21)00338-1

34. Pasolli E, Schiffer L, Manghi P, Renson A, Obenchain V, Truong DT, Beghini F, Malik F, Ramos M, Dowd JB, Huttenhower C, Morgan M, Segata N, Waldron L. Accessible, curated metagenomic data through ExperimentHub. *Nat methods*. 2017;14(11):1023-1024. doi:10.1038/nmeth.4468

35. Geier RR, Rehberger TG, Smith AH. Comparative Genomics of Clostridium perfringens Reveals Patterns of Host-Associated Phylogenetic Clades and Virulence Factors. *Front Microbiol*. 2021;12:649953. doi:10.3389/fmicb.2021.649953

36. Ohtani K, Yuan Y, Hassan S, Wang R, Wang Y, Shimizu T. Virulence gene regulation by the agr system in Clostridium perfringens. *J Bacteriol*. 2009;191(12):3919-3927. doi:10.1128/jb.01455-08

37. Nagpal R, Ogata K, Tsuji H, Matsuda K, Takahashi T, Nomoto K, Suzuki Y, Kawashima K, Nagata S, Yamashiro Y. Sensitive quantification of Clostridium perfringens in human feces by quantitative real-time PCR targeting alpha-toxin and enterotoxin genes. *Bmc Microbiol*. 2015;15(1):219. doi:10.1186/s12866-015-0561-y

38. Asnicar F, Weingart G, Tickle TL, Huttenhower C, Segata N. Compact graphical representation of phylogenetic data and metadata with GraPhlAn. *PeerJ*. 2015;3:e1029. doi:10.7717/peerj.1029

39. Zimmermann M, Zimmermann-Kogadeeva M, Wegmann R, Goodman AL. Mapping human microbiome drug metabolism by gut bacteria and their genes. *Nature*. 2019;570(7762):462-467. doi:10.1038/s41586-019-1291-3
